# High glucose couples DJ-1 with PTEN to activate PDGFRβ for renal proximal tubular cell injury

**DOI:** 10.1371/journal.pone.0311828

**Published:** 2025-01-06

**Authors:** Falguni Das, Nandini Ghosh-Choudhury, Balakuntalam S. Kasinath, Kumar Sharma, Goutam Ghosh Choudhury

**Affiliations:** 1 VA Research, Education and Clinical Center, South Texas Veterans Health Care System, San Antonio, Texas, United States of America; 2 Departments of Medicine, UT Health San Antonio, San Antonio, Texas, United States of America; 3 Departments of Pathology, UT Health San Antonio, San Antonio, Texas, United States of America; 4 Geriatric Research, Education and Clinical Center, South Texas Veterans Health Care System, San Antonio, Texas, United States of America; King Faisal Specialist Hospital and Research Center, SAUDI ARABIA

## Abstract

High glucose milieu in diabetes induces proximal tubular epithelial cells in the kidney to undergo hypertrophy and matrix protein expansion via Akt/mTORC1 signaling, leading to renal fibrosis. The familial Parkinson’s disease protein DJ-1 acts as a driver of Ras-dependent tumorigenesis and protects dopaminergic neurons from apoptosis. But its function and mechanistic basis to regulate renal fibrosis is not clear. Here, we identify DJ-1 as a high glucose-promoted protein in renal proximal tubular epithelial cells. Mechanistic interrogation revealed that DJ-1 formed complex with the lipid phosphatase PTEN in high glucose-stimulated cells, resulting in phosphorylation/activation of Akt and mTORC1. siRNAs against DJ-1 decreased high glucose-stimulated Akt/mTORC1 activation. In contrast, overexpression of DJ-1 mimicked all effects of high glucose. Interestingly, inhibition of DJ-1 blocked high glucose-induced hypertrophy of proximal tubular epithelial cells and, matrix proteins fibronectin and collagen I (α2) expression while overexpression of DJ-1 mimicked the high glucose effects on these phenomena. Previously, we reported a role of PDGFRβ in proximal tubular cell injury. In exploring the mechanism of DJ-1 function, we found that siDJ-1 inhibited high glucose-induced activating and PI 3 kinase docking site tyrosine phosphorylation of PDGF receptor-β (PDGFRβ) to block phosphorylation of PI 3 kinase. Interestingly, overexpression of PTEN mimicked these effects of siDJ-1. Together, our results reveal an important role of DJ-1-PTEN nodal point for PDGFRβ activation during high glucose-induced proximal tubular epithelial cell injury.

## Introduction

More than 537 million people worldwide have diabetes [1]. Diabetic kidney disease is a leading cause of chronic kidney disease characterized by sustained increase in urinary albumin levels and loss of clearance function. Approximately, 30 and 40% of patients with type 1 and type 2 diabetes respectively develop chronic kidney disease [2]. Between 1990 and 2013, the death of patients with diabetic kidney disease increased significantly mainly due to cardiovascular complications [3]. In fact, diabetes contributes to the 50% of patients with end stage renal disease [4]. Microvascular complications of diabetes includes microalbuminuria followed by macroalbuminuria leading to loss of kidney function [5]. Although glomerular cells contribute significantly, the proximal tubular cells also play important role in the pathologies of diabetic kidney disease via tubuloglomerular feedback including initial renal hypertrophy followed by thickening of the tubular basement membrane and interstitial fibrosis [6, 7]. Furthermore, hyperglycemia-induced autocrine and paracrine growth factors and cytokines including transforming growth factor-β (TGFβ) and platelet-derived growth factor (PDGF) promote partial epithelial to mesenchymal trans-differentiation leading to accumulation of extracellular matrix proteins causing fibrosis of the kidney [8, 9].

Five different homo- and hetero-dimeric isoforms of PDGF containing AA-, BB, AB-, CC- and DD-chains have been identified [10]. These ligands bind to two receptors, PDGFRα and PDGFRβ, with variable affinity and specificity [11]. In the kidney, PDGF isoforms are predominantly expressed in the glomerular mesangial cells. In mice, deletion of PDGF A- or C- or D-chains showed no obvious phenotype [12, 13]. In contrast, deletion of either PDGF B-chain or PDGFRβ shows defects in the development of glomerular mesangial cells in the kidney demonstrating a critical role of this ligand receptor complex in nephrogenesis [14, 15]. Furthermore, in the glomerulonephritis patients and in rodent models of mesangioproliferative glomerulonephritis both B-chain and PDGFRβ are increased [16–18]. Similarly, in a rat model of diabetic kidney disease, glomerular increase in both PDGF B and PDGFRβ is found [19]. Interestingly, injured tubular epithelial cells in patients with diabetic nephropathy express heightened levels of both A- and B- chains [20]. The mechanism how PDGFRβ may be activated in the proximal tubular epithelial cells in hyperglycemia is not clear.

DJ-1 was discovered as an oncogene, which when transfected could transform NIH 3T3 fibroblasts [21]. However, when co-transfected with Ras, it increased the transforming potential 3-fold compared to Ras/Myc combination [21]. However, missense and deletion mutations found in DJ-1 lead to early onset autosomal Parkinson’s disease due to aggregation of the protein [22–24]. DJ-1 acts as an antioxidant protein although it lacks any ROS detoxifying enzyme activity [25]. Importantly, Cys-106 residue, which acts as a nucleophilic elbow to undergo oxidation, has been shown to be essential for the protective effects in neurons from oxidative stress-mediated apoptosis in patients with Parkinson’s disease [26–28]. DJ-1 also cooperates with androgen receptor during fertilization and spermatogenesis [29]. Although it is ubiquitously expressed and has significant structural similarity with the bacterial protease Pfp1/PJ1704, the catalytic site of DJ-1 is occluded and distorted. As a result, it does not have any protease activity [28]. However, DJ-1 has been shown to have glyoxalase II and deglycase activities to detoxify carbonyl species and repair glycation damage in proteins and nucleic acids [30–32]. DJ-1 also acts as an oncogene and many tyrosine kinases such as PDGFRβ have been shown to be activated during oncogenesis to drive mTOR activity, which also plays important role in renal fibrosis [11, 33]. Although PDGFRβ is activated in the kidneys in patients with diabetic kidney disease, the mechanism of its activation is not known. We hypothesized that DJ-1 may cooperate with PDGFRβ to activate mTORC1 in the accumulation of matrix proteins during the progression of renal fibrosis. In this study, using proximal tubular epithelial cells, we found that high glucose-induced increase in DJ-1 activates PDGFRβ. We observed that PTEN (phosphatase and tensin homolog deleted in chromosome 10) acts as a PDGFRβ phosphatase and interacts with DJ-1 to activate PDGFRβ for Akt/mTORC1 activation. Therefore, we identify an important role of DJ-1/PTEN/PDGFRβ/mTORC1 axis in driving renal fibrosis.

## Materials and methods

### Reagents

Reagents for culturing cells and OPTIMEM transfection medium were obtained from Thermo Fisher. *D*-glucose, *D*-mannitol, NP-40, Na_3_VO_4_, PMSF, protease inhibitor cocktail and antibodies against fibronectin and FLAG were purchased from Sigma. DJ-1, actin, collagen I (α2) and PTEN antibodies were acquired from Santa Cruz. Following antibodies were obtained from Cell Signaling: p-Akt (Ser-473), Akt, p-GSK3β (Ser-9), GSK3β, p-Tuberin (Thr-1462), tuberin, p-PRAS40 (Thr-246), p-4EBP-1 (Thr-37/46), 4EBP-1, p-S6 kinase (Thr-389), S6 kinase, p-mTOR (Ser-2448), mTOR, raptor, p-p85 PI 3 kinase (Tyr- 458), p85, p-PDGFRβ (Tyr-857), p-PDGFRβ (Tyr- 740), p-PDGFRβ (Tyr-751), PDGFRβ. Anti-HA antibody was obtained from Covance. ^35^S-Methionine and PVDF membrane were purchased from Perkin Elmer. Pooled silent interfering RNAs (siRNAs) against DJ-1 and PTEN and scrambled RNAs were acquired from Santa Cruz. FLAG-tagged DJ-1 and HA-tagged PTEN plasmid expression vectors were kind gifts from Dr. H. Ariga (Hokkaido University, Japan) and William Seller (Dana-Farber Cancer Institute), respectively. shRaptor and dominant negative Akt K179M plasmids have been described previously [34, 35].

### Cell culture

We purchased HK2 human proximal tubular epithelial cells from ATCC. The cells were grown in T75 flasks in DMEM/F12 culture medium in the presence of 10% fetal bovine serum [36]. For performing experiments, the cells were grown to confluency and starved in serum-free medium for 24 hours. The cells were then incubated with 25 mM glucose (HG) for indicated periods of time. Diabetic patients often attain this concentration of glucose in their serum. Also, rodent models such as Akita mice, which are used in diabetes studies, exhibit average of 450 mg/dL (25 mM) blood glucose level [37]. As osmotic control, the cells were incubated with 5 mM glucose plus 20 mM mannitol.

### Lysis of cells and immunoblotting

At the end of incubation with high glucose, the cells were lysed in RIPA buffer followed by centrifugation to collect the supernatant essentially as described previously [36, 38]. 25 μg protein from cell lysates were used for electrophoresis and immunoblotting as described before [36, 38].

### Immunoprecipitation

PBS-washed cell monolayer was incubated with immunoprecipitation (IP) buffer (40 mM HEPES, pH 7.5, 1 mM EDTA, 120 mM NaCl, 0.3% CHAPs, 50 mM NaF, 10 mM pyrophosphate, 1.5 mM Na_3_VO_4_ and 0.1% protease cocktail) at 4°C for 30 minutes. 100 μg cell extracts were centrifuged as described above. The protein concentration was determined in the supernatant. Equal amounts of protein were incubated with specific antibody at 4°C for 30 minutes prior to addition of protein-G Sepharose beads. The mixture was then incubated overnight on a rotating device at 4°C. The mixture was centrifuged for couple of seconds to separate the beads. The supernatant was discarded, and the beads were washed three times by brief centrifugation with IP buffer at 4°C. Finally, the beads were resuspended in SDS sample buffer, separated by gel electrophoresis and transferred to PVDF membrane and immunoblotted with specific antibody as described above.

### Protein synthesis and hypertrophy

To measure protein synthesis induced by high glucose, the cells were incubated with 1 μCi/ml ^35^S-Methionine. Radioactive incorporation was determined as TCA precipitable ^35^S-Methionine incorporation as described previously [36, 39]. Proximal tubular epithelial cell hypertrophy was determined as a ratio of total protein to cell number essentially as described previously [40, 41].

### Transfection

Transfection of proximal tubular epithelial cells was carried out using FuGENE HD as described previously [36, 38, 40].

### Statistics

The data were expressed as mean ± SD and the significance was determined using GraphPad Prism software by paired t-test or ANOVA. A *p*-value of less than 0.05 was considered as significant.

## Results

### High glucose enhances expression of DJ-1

**Fig 1:**
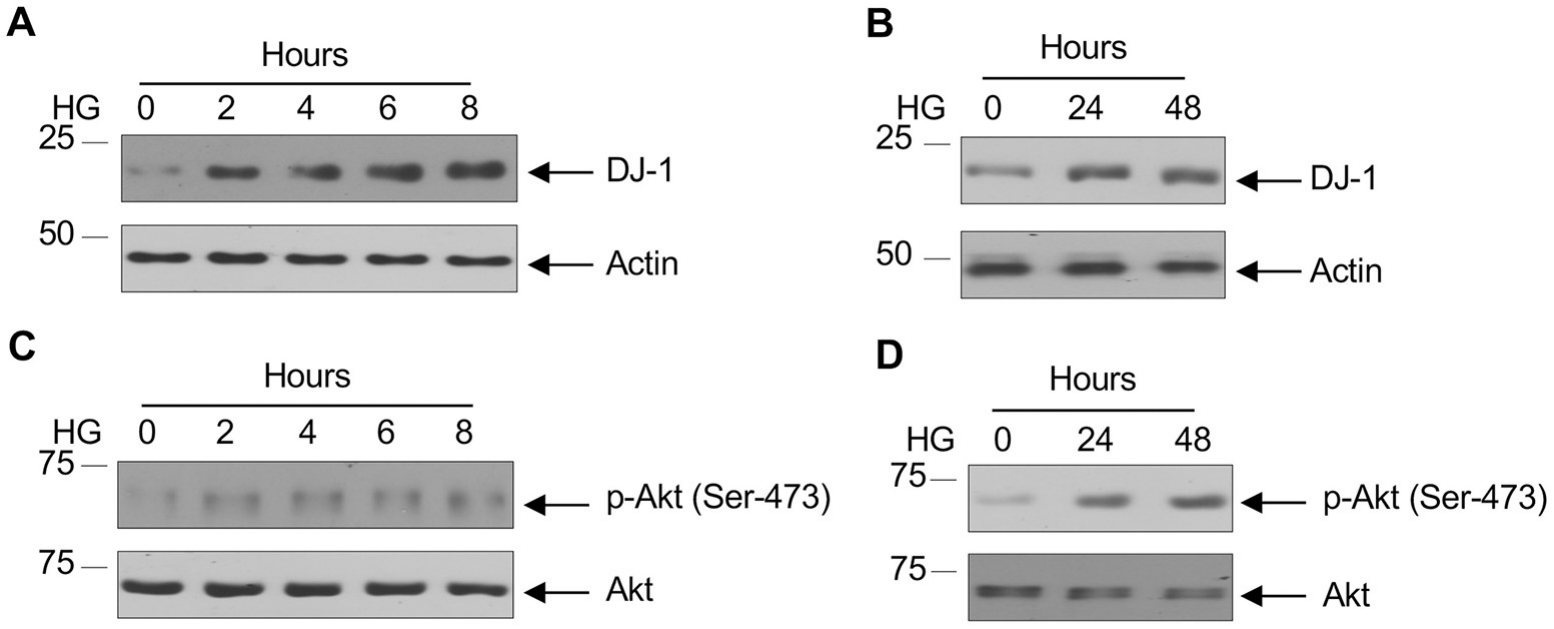
High glucose increases expression of DJ-1 concomitant with Akt phosphorylation. Serum-starved human proximal tubular epithelial cells were incubated with 5 mM glucose plus 20 mM mannitol (0 hr) or 25 mM glucose for the indicated periods of time. Cell lysates were immunoblotted with DJ-1 and actin antibodies (panels A and B). In panels C and D, the cell lysates were immunoblotted with phosphor-Akt (Ser-473) and Akt antibodies as indicated. Representative of 3–5 experiments is shown.

Hyperglycemia induces proximal tubular epithelial cells to initiate signaling programs to promote renal fibrosis [42]. Contribution of DJ-1 has been extensively studied in the context of Parkinson’s disease in dopaminergic neurons and in tumorigenesis [22–24, 43, 44]. Its role in driving pathologies of diabetic kidney disease has not been investigated. To mimic diabetic milieu, we exposed proximal tubular epithelial cells to 25 mM glucose (high glucose). High glucose increased expression of DJ-1 in a sustained manner (Fig 1A and 1B and S1A and S1B Fig). In cancer, DJ-1 interacts with Akt signaling pathway to drive proliferation of tumor cells [43]. We have previously shown a significant role of Akt kinase in the pathogenesis of diabetic kidney disease [39, 41, 45, 46]. We examined phosphorylation of Akt in proximal tubular epithelial cells. Similar to increased expression of DJ-1, high glucose significantly enhanced the phosphorylation of Akt in a sustained manner (Fig 1C and 1D and S1C and S1D Fig).

### High glucose increases association of DJ-1 with PTEN

**Fig 2:**
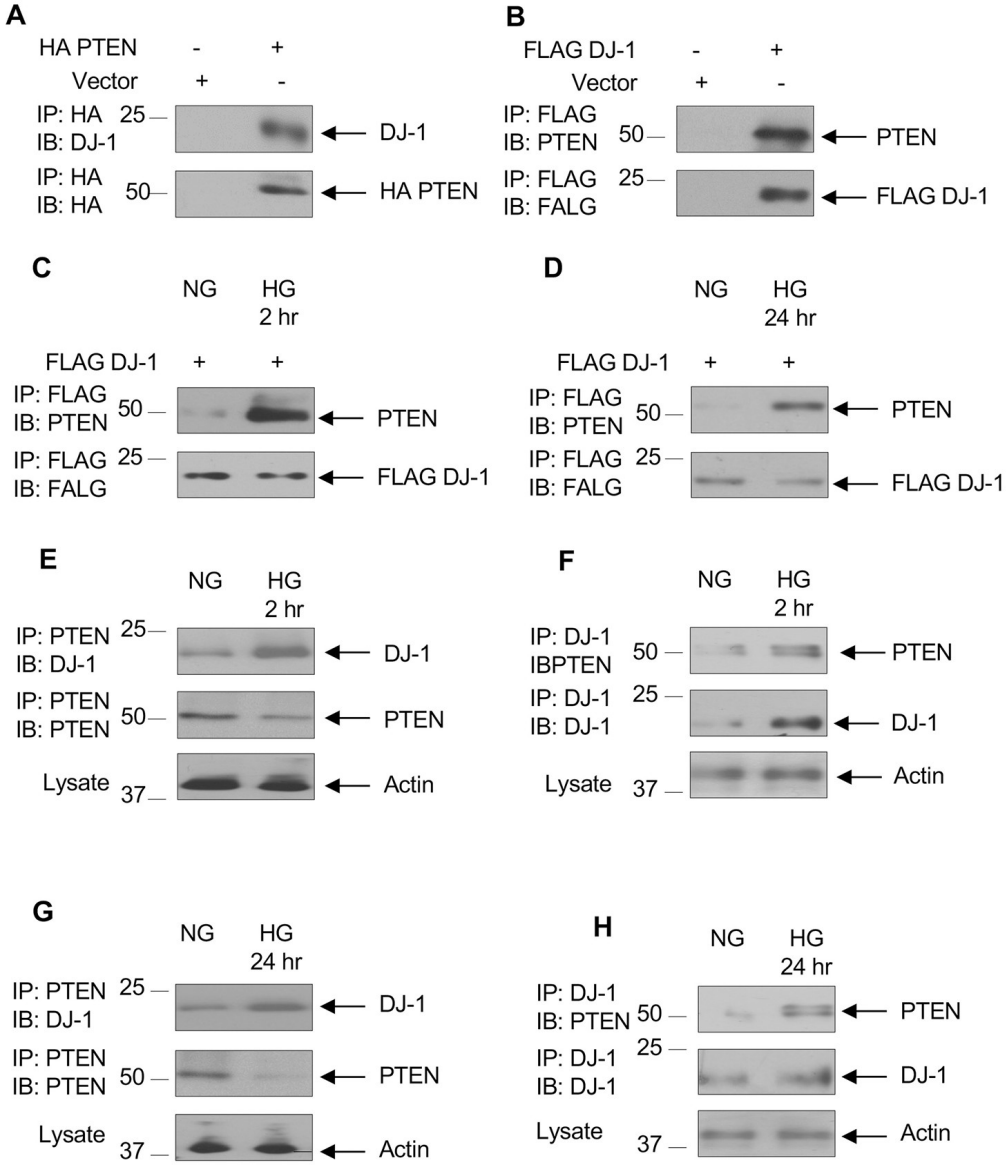
High glucose induces association of DJ-1 with PTEN. (A and B) HA-tagged PTEN (panel A) or FLAG-tagged DJ-1 (panel B) was transfected into human proximal tubular epithelial cells. The cell lysates were immunoprecipitated with antibodies against HA (panel A) or FLAG (panel B). The immunoprecipitates (IP) were immunoblotted (IB) with DJ-1, HA (panel A) and PTEN, FLAG (panel B) antibodies. (C and D) Proximal tubular epithelial cells were transfected with FLAG-tagged DJ-1 prior to incubation with 5 mM glucose plus 20 mM mannitol (normal glucose, NG) or 25 mM glucose (high glucose, HG) for 2 hrs (panel C) and 24 hours (panel D). The cell lysates were immunoprecipitated with FLAG antibody followed by immunoblotted with antibodies against PTEN and FLAG. (E–H) Serum-starved proximal tubular epithelial cells were incubated with high glucose for 2 hr (panels E and F) or 24 hr (panels G and H). The cell lysates were immunoprecipitated with antibodies against PTEN (panels E and G) or DJ-1 (panels F and H) followed by immunoblotting with the indicated antibodies. The lower panels show actin blots in the cell lysates. Representative of 3 experiments is shown.

One mechanism of activation of Akt is the production of phosphatidylinositol 3,4,5 tris-phosphate (PIP_3_) by PI 3 kinase [47]. The level of PIP_3_ in cell is regulated by the activity of PTEN, a PIP_3_ phosphatase [48]. Thus reduced PTEN increases Akt activity. We have reported that high glucose decreases the expression of PTEN in renal cells and in diabetic rodent kidneys to activate Akt [39, 46, 49]. Furthermore, multiple proteins have been shown to interact with PTEN to inhibit its activity [50, 51]. We tested the hypothesis that PTEN may interact with DJ-1 in proximal tubular epithelial cells. First, we overexpressed HA-tagged PTEN to examine its association with endogenous DJ-1. Immunoprecipitation of HA showed complex formation between PTEN and DJ-1 (Fig 2A). Similarly, FLAG-tagged DJ-1 when transfected into proximal tubular epithelial cells showed association with PTEN (Fig 2B). Next, we examined the effect of high glucose on this association. FLAG-tagged DJ-1-transfected proximal tubular epithelial cells were exposed to high glucose for 2 or 24 hours. In both conditions, FLAG immunoprecipitates showed increased association of PTEN with DJ-1 in response to high glucose (Fig 2C and 2D and S2A and S2B Fig). To identify whether endogenous DJ-1 associates with PTEN, we incubated proximal tubular epithelial cells with high glucose for 2 hours. Immunoprecipitation of PTEN followed by immunoblotting with DJ-1 showed increased association of these proteins by high glucose (Fig 2E and S2C Fig). Reciprocal immunoprecipitation/immunoblotting confirmed these results (Fig 2F and S2D Fig). Similarly, high glucose for 24 hours increased the association of PTEN and DJ-1 (Fig 2G and 2H and S2E and S2F Fig). Importantly, complete glucose starvation had similar effect on association of DJ-1 with PTEN as with incubation with 5 mM glucose (S3A–S3D Fig). It is predicted that increased association between DJ-1 and PTEN may result from increased expression of DJ-1 by high glucose (Figs 1A and 1B and 2F and 2H). However, high glucose decreased the expression of PTEN (Fig 2E and 2G, middle panels). These results are in agreement with our previous observation [39, 46, 49]. Thus, these data indicate increased association of PTEN with DJ-1 in the presence of decreased PTEN levels in high glucose-treated proximal tubular epithelial cells.

### DJ-1 regulates activation of Akt

**Fig 3:**
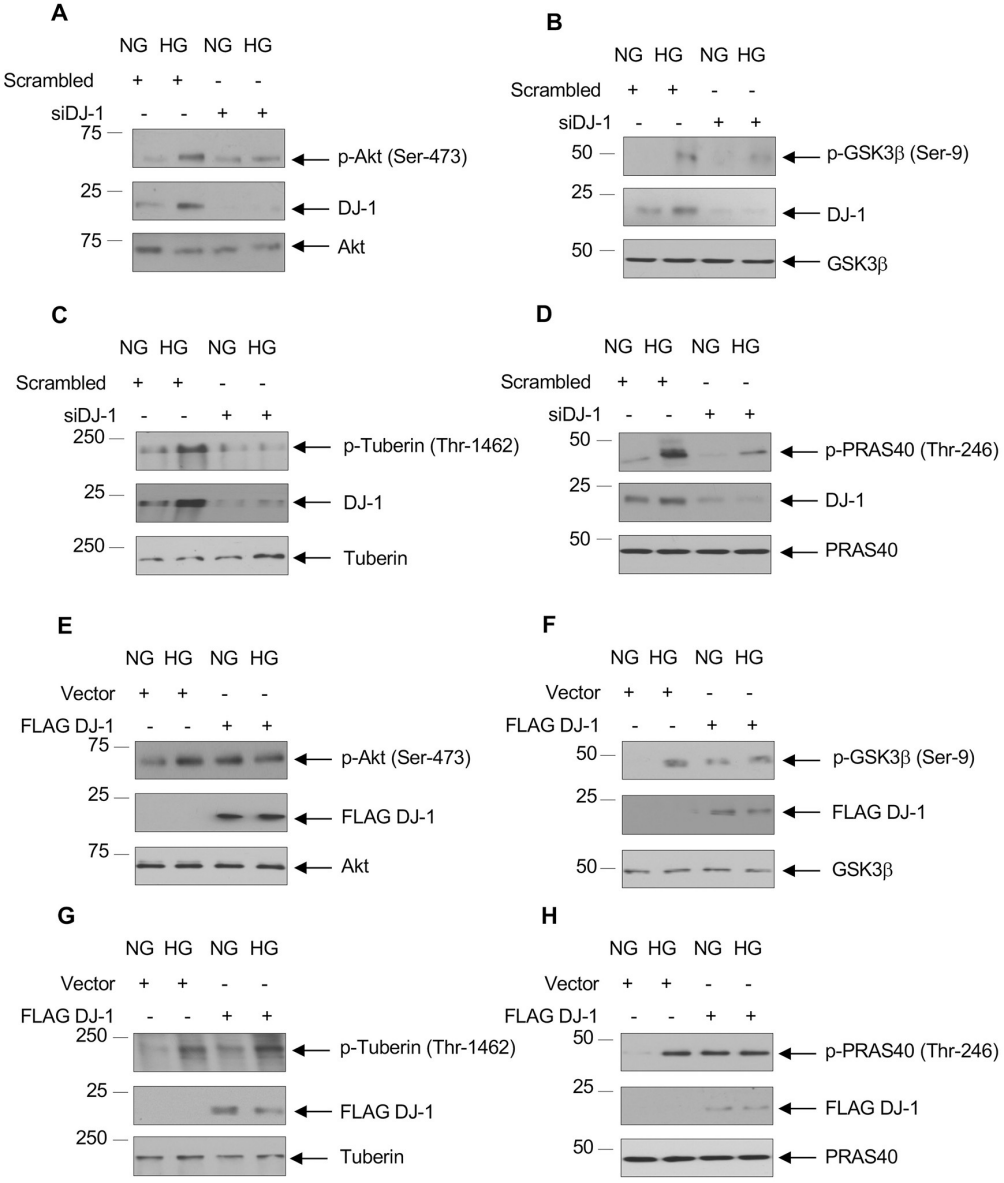
DJ-1 regulates high glucose-stimulated Akt kinase activation and phosphorylation of its substrates. Human proximal tubular epithelial cells were transfected with siRNAs against DJ-1 (panels A–D) or FLAG-tagged DJ-1 (panels E–H) prior to incubation with HG for 24 hours. The cell lysates were immunoblotted with phospho-Akt (Ser-473) and Akt antibodies (panels A and E), phospho-GSK3β (Ser-9) and GSK3β antibodies (panels B and F), phospho-tuberin (Thr-1462) and tuberin antibodies (panels C and G), phospho-PRAS40 (Thr-246) and PRAS40 antibodies (panels D and H). Lysates were also immunoblotted with DJ-1 and FLAG antibodies as indicated. Representative of 3 experiments is shown.

Our results above showed concomitant increase in Akt phosphorylation with enhanced expression of DJ-1 by high glucose (Fig 1). We examined whether DJ-1 is involved in activation of Akt. We transfected siRNAs against DJ-1 into proximal tubular epithelial cells. Knockdown of DJ-1 inhibited high glucose-stimulated phosphorylation of Akt (Fig 3A and S4A Fig). Phosphorylation of Akt increases its activity. Therefore, we tested the phosphorylation of its endogenous substrate GSK3β. High glucose increased the phosphorylation of GSK3β. This phosphorylation was blocked by siRNAs against DJ-1 (Fig 3B and S4B Fig). Two other substrates of Akt are the GTPase activating protein tuberin and the mTORC1 component PRAS40. siDJ-1 also inhibited the high glucose-stimulated phosphorylation of these proteins (Fig 3C and 3D and S4C and S4D Fig). To confirm these observations, we used FLAG-tagged DJ-1. Expression of DJ-1 increased Akt phosphorylation and resulting phosphorylation of GSK3β, tuberin and PRAS40 (Fig 3E–3H and S4E–S4H Fig).

### DJ-1 increases mTORC1 activity

**Fig 4:**
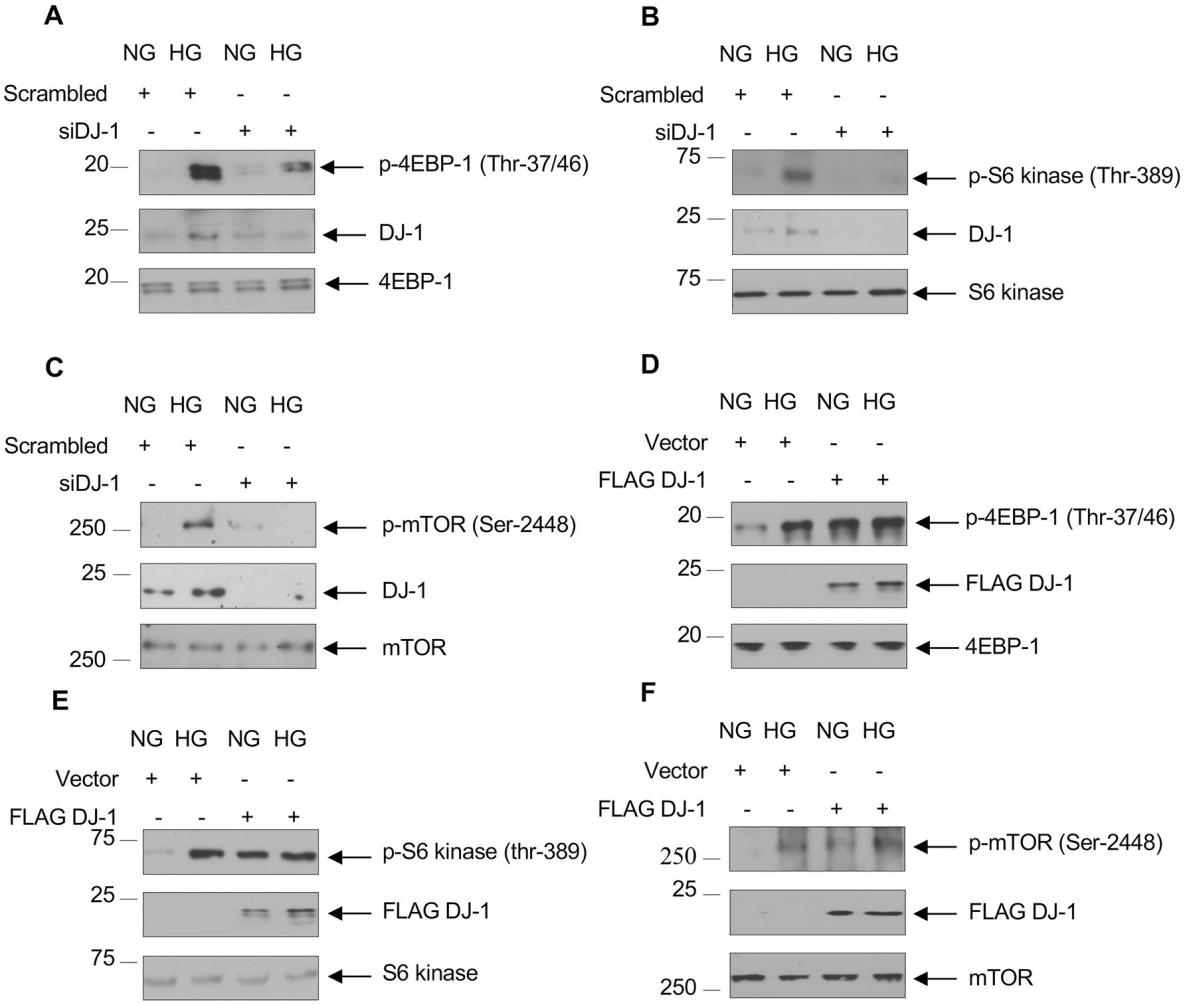
DJ-1 regulates high glucose-stimulated mTORC1 activation. Human proximal tubular epithelial cells were transfected with siRNAs against DJ-1 (panels A–C) or FLAG-tagged DJ-1 (panels D–F) prior to incubation with HG for 24 hours. The cell lysates were immunoblotted with phospho-4EBP-1 (Thr-37/46), 4EBP-1 (panels A and D), phsopho-S6 kinase (Thr-389) and S6 kinase (panels B and E), phospho-mTOR (Ser-2448) and mTOR antibodies (panels C and E). Lysates were also immunoblotted with DJ-1 and FLAG antibodies as indicated. Representative of 3 experiments is shown.

We have shown previously that high glucose promotes mTORC1 activity via Akt [45, 49, 52, 53]. Unphosphorylated tuberin and PRAS40 constitutively inhibit mTORC1 activity. Phosphorylation of these proteins by Akt inactivates their function, resulting in activation of mTORC1 [52, 54, 55]. We tested the role of DJ-1 in activation of mTORC1. siRNAs against DJ-1 inhibited high glucose-stimulated phosphorylation of 4EBP-1 an endogenous substrate of mTORC1 (Fig 4A and S5A Fig). Similarly, siDJ-1 abrogated the high glucose-induced phosphorylation of S6 kinase, another substrate of mTORC1 (Fig 4B and S5B Fig). Phosphorylation of S6 kinase increases its activity, resulting in phosphorylation of its substrate mTOR [56]. siDJ-1 inhibited mTOR phosphorylation in response to high glucose (Fig 4C and S5C Fig). In confirming these observations, we found overexpression of DJ-1 in normal glucose-treated cells increased the phosphorylation of 4EBP-1, S6 kinase and mTOR similar to treatment with high glucose (Fig 4D–4F and S5D–S5F Fig). Together, our results demonstrate regulation of mTORC1 by DJ-1 via Akt.

### DJ-1 regulates high glucose-induced proximal tubular cell hypertrophy and matrix protein expression

**Fig 5:**
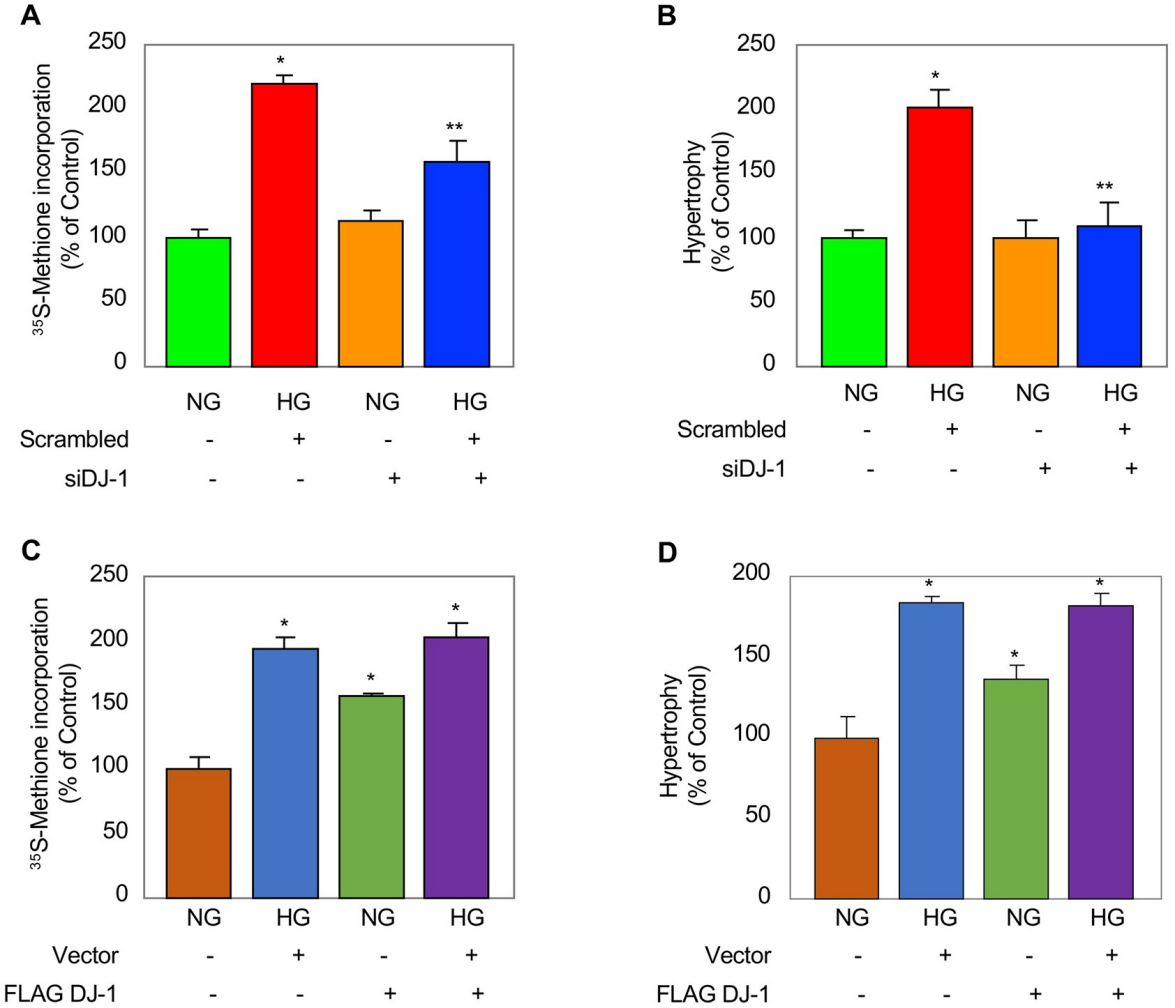
DJ-1 regulates proximal tubular epithelial cell protein synthesis and hypertrophy. Human proximal tubular epithelial cells were transfected with siRNAs against DJ-1 (panels A and B) or FLAG-tagged DJ-1 (panels C and D) prior to incubation with HG for 24 hours. In panels A and C, protein synthesis and in panels B and D, hypertrophy were determined as described in the Materials and Methods. Error bars with standard deviation of triplicate measurements is shown. In panels A and B, *p < 0.001 vs NG; **p < 0.001 vs HG. In panels C and D, *p < 0.001–0.05 vs NG alone.

We have previously provided evidence that mTORC1 regulates renal cell hypertrophy [41, 45, 49, 52, 57]. Since we have shown above that DJ-1 controls mTORC1 activity, we examined its effect on proximal tubular cell hypertrophy. siRNAs against DJ-1 significantly inhibited high glucose-induced protein synthesis, a measure of cell hypertrophy (Fig 5A). Similarly, siDJ-1 attenuated high glucose-induced proximal tubular cell hypertrophy as determined by the ratio of cell protein to cell number (Fig 5B). In contrast to these results, when we overexpressed DJ-1, it induced protein synthesis and hypertrophy similar to treatment with high glucose (Fig 5C and 5D).

**Fig 6:**
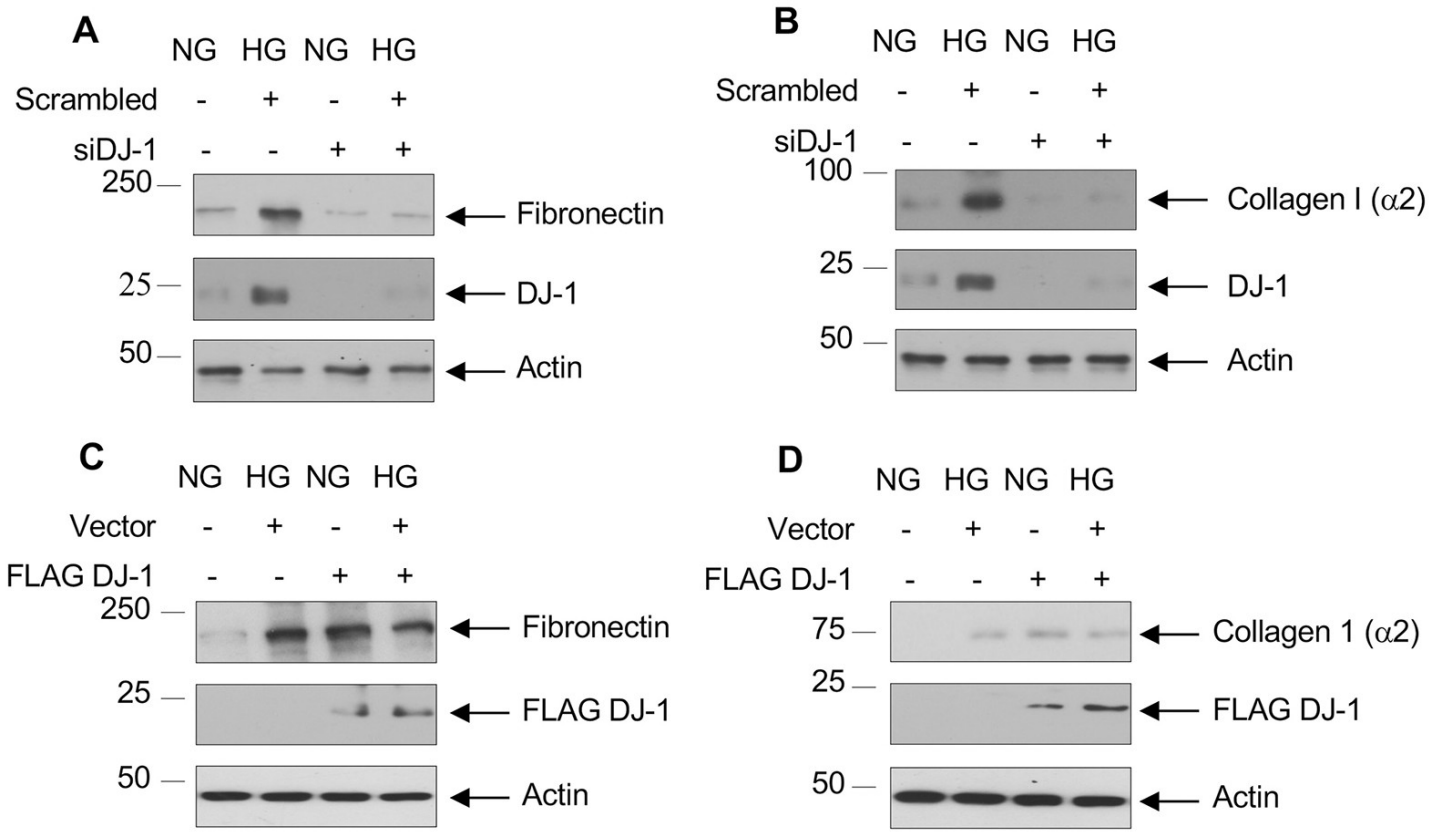
DJ-1 controls high glucose-stimulated expression of fibronectin and collagen I (α2). Human proximal tubular epithelial cells were transfected with siRNAs against DJ-1 (panels A and B) or FLAG-tagged DJ-1 (panels C and D) prior to incubation with HG for 24 hours. The cell lysates were immunoblotted with fibronectin (panels A and C) and collagen I (α2) (panels B and D) antibodies. Cell lysates were also immunoblotted with DJ-1 and FLAG antibodies as indicated. Representative of 3 experiments is shown.

We and others have demonstrated a role of mTORC1 in diabetic renal fibrosis [58–64]. Since we showed above that DJ-1 regulates mTORC1 activity, we determined the involvement of DJ-1 in the expression of two fibrotic proteins, fibronectin and collagen I (α2). Exposure of proximal tubular epithelial cells to high glucose promoted the expression of fibronectin and collagen I (α2) (Fig 6). siRNAs against DJ-1 inhibited high glucose-stimulated expression of both these matrix proteins (Fig 6A and 6B and S6A and S6B Fig). In contrast, expression of DJ-1 increased both fibronectin and collagen I (α2) levels similar to treatment with high glucose (Fig 6C and 6D and S6C and S6D Fig).

### DJ-1 regulates hypertrophy and matrix protein expression via Akt/mTORC1

**Fig 7:**
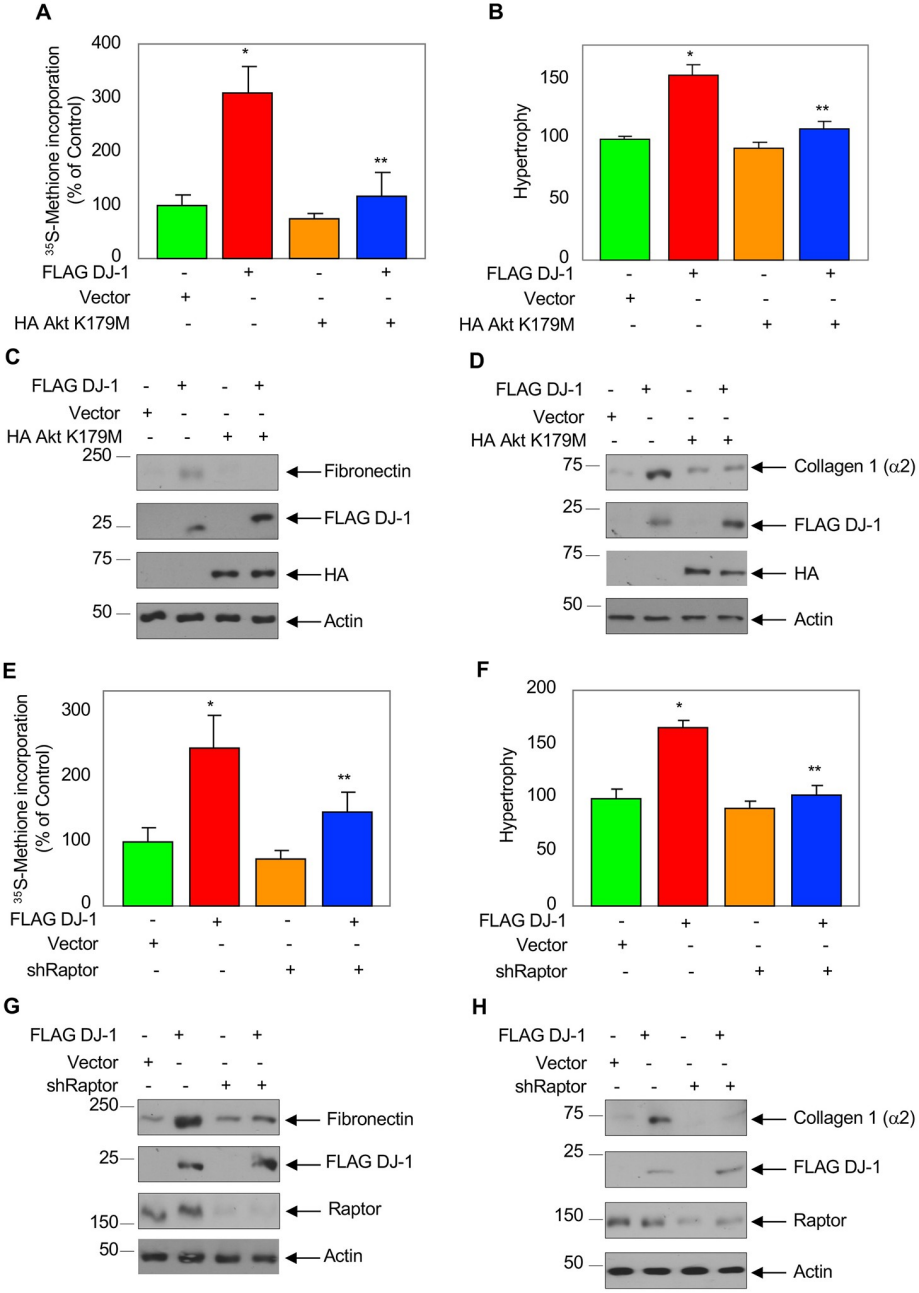
Akt/mTORC1 regulates DJ-1-induced proximal tubular epithelial cell hypertrophy and matrix protein expression. Proximal tubular epithelial cells were co-transfected with FLAG-DJ-1 and HA Akt K179M (A–D) or shRaptor (E–H). (A, B, E and F) Protein synthesis and hypertrophy were measured as described in the materials and methods. Mean ± 3–6 measurements is shown. *p < 0.001–0.01 vs control; **p < 0.001–0.01 vs HG. (C, D, G and H) Expression of fibronectin and collagen I (a2) were determined in the cell lysates using their antibodies. Antibodies against FLAG and HA were used to detect expression of DJ-1 and Akt K179M.

We have shown above that DJ-1 affects high glucose-stimulated proximal tubular cell hypertrophy and matrix protein expression. However, is DJ-1-mediated activation of Akt/mTORC1 needed for these phenomena? To address this, we transfected cells with FLAG-tagged DJ-1 and dominant negative Akt (K179M). As expected, expression of DJ-1 increased protein synthesis and induced hypertrophy. Both events were inhibited by dominant negative Akt (Fig 7A and 7B). Similarly, expression of dominant negative Akt significantly blocked DJ-1-stimulated expression of fibronectin and collagen I (α2) (Fig 7C and 7D and S7A and S7B Fig). Furthermore, expression of shRaptor, which inhibits DJ-1-stimulated mTORC1 activity (S7C and S7D Fig), blocked DJ-1-induced protein synthesis, hypertrophy, and fibronectin/collagen I (α2) expression (Fig 7E–7H and S7E and S7F Fig). Thus, our results demonstrate requirement of Akt/mTORC1 for DJ-1-induced proximal tubular cell pathologies.

### DJ-1 controls PDGFRβ activating phosphorylation

**Fig 8:**
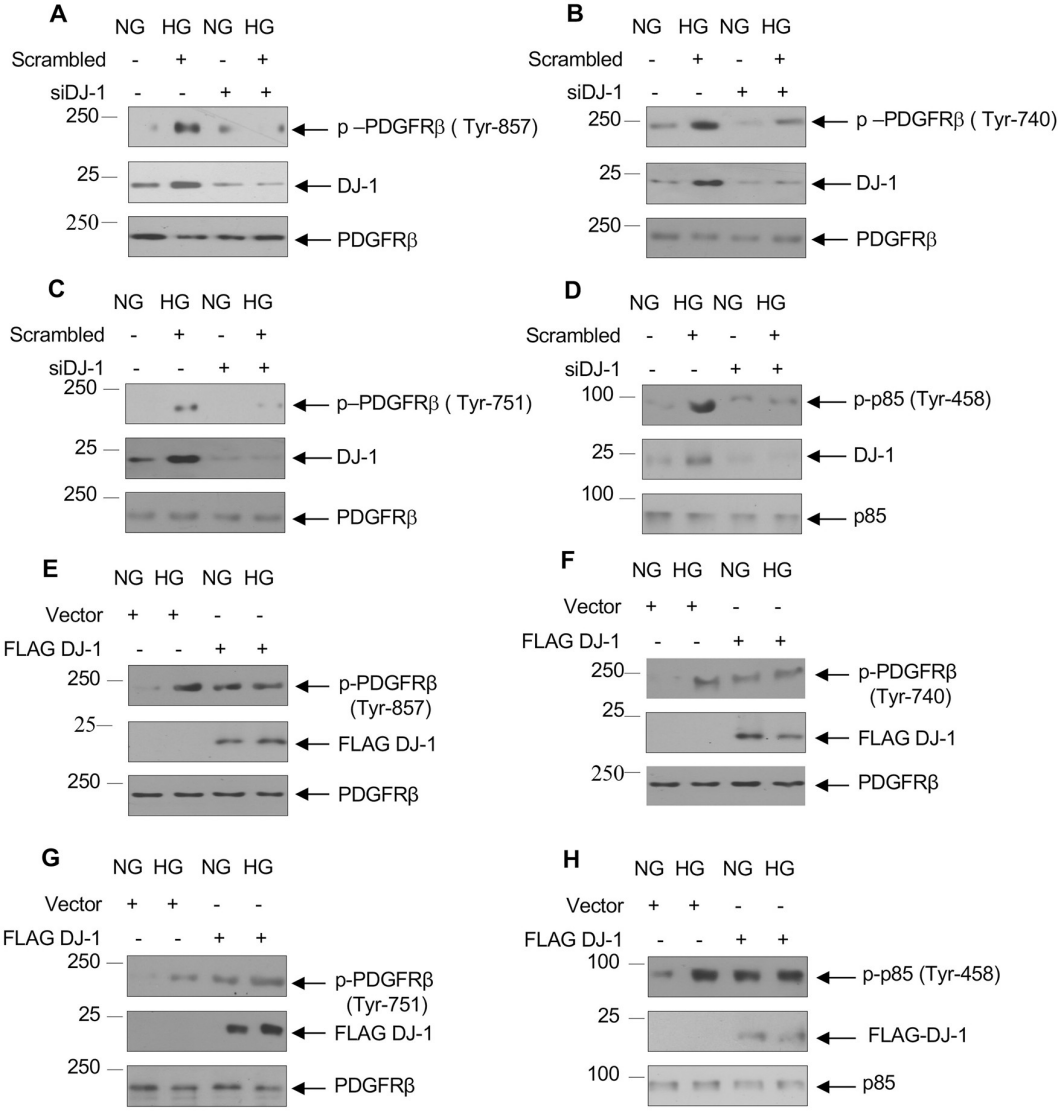
DJ-1 regulates high glucose-stimulated tyrosine phosphorylation of PDGFRβ. Human proximal tubular epithelial cells were transfected with siRNAs against DJ-1 (panels A—D) or FLAG-tagged DJ-1 (panels E—H) prior to incubation with HG for 24 hours. The cell lysates were immunoblotted with phospho-PDGFRβ (Tyr-857) (panels A and E), phospho-PDGFRβ (Tyr-740) (panels B and F), phospho-PDGFRβ (Tyr-751) (panels C and G), PDGFRβ, DJ-1 and FLAG antibodies as indicated. In panels D and H, the cell lysates were immunoblotted with phospho-p85 PI 3 kinase (Tyr-458), p85, DJ-1 and FLAG antibodies as indicated. Representative of 3 experiments is shown.

Role of PDGF BB and PDGFRβ in renal pathologies is well documented [17, 20, 65, 66]. Increased renal expression of PDGFRβ in human diabetic kidneys is reported [67]. Recently, we have demonstrated a role of PDGFRβ in high glucose-induced renal glomerular cell pathology [68]. We have shown above a role of DJ-1 in proximal tubular epithelial cell hypertrophy and matrix protein expression. But is DJ-1 required for PDGFRβ activation? Phosphorylation of Tyr-857 in the activation loop indicates activation of PDGFRβ [69]. Incubation of proximal tubular epithelial cells with high glucose increased time-dependent phosphorylation of Tyr-857 residue in PDGFRβ (S8A–S8D Fig) [70]. siRNAs against DJ-1 inhibited high glucose-stimulated activating phosphorylation of PDGFRβ (Fig 8A and S9A Fig). Activated PDGFRβ undergoes autophosphorylation at multiple sites to generate docking sites for signaling enzymes. Two such sites in PDGFRβ are Tyr-740 and Tyr-751, which when phosphorylated provide binding sites for PI 3 kinase [71]. We previously reported that high glucose enhanced phosphorylation of these PI 3 kinase binding sites in time-dependent and sustained manner [70]. This phosphorylation of PDGFRβ resulted in increased phosphorylation of PI 3 kinase, indicating its association with the PDGFRβ [69, 70]. To examine the role of DJ-1 in these phosphorylations of PDGFRβ and PI 3 kinase, we used siRNAs against DJ-1. siDJ-1 inhibited high glucose-stimulated tyrosine-740/751 phosphorylation and PI 3 kinase tyrosine phosphorylation (Fig 8B–8D and S9B–S9D Fig). To confirm these observations, we transfected DJ-1. Overexpression of DJ-1 increased the phosphorylation of PDGFRβ at Tyr-857, Tyr-740, Tyr-751 and PI 3 kinase at Tyr-458 similar to treatment with high glucose (Fig 8E–8H and S9E–S9H Fig). These results conclusively show that DJ-1 regulates PDGFRβ activation.

### PDGFRβ is a target of PTEN

**Fig 9:**
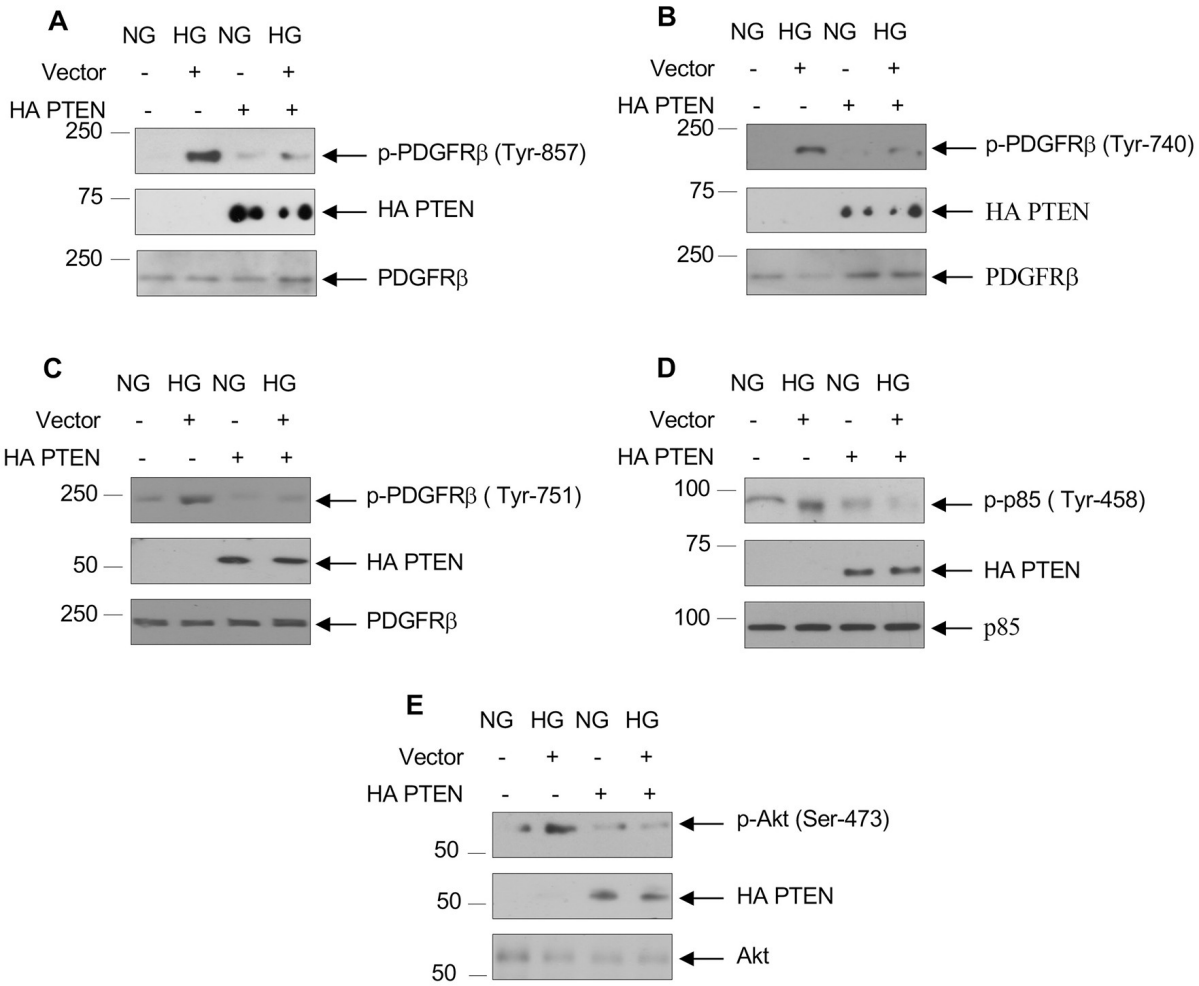
PTEN regulates high glucose-induced PDGFRβ tyrosine phosphorylation. Human proximal tubular epithelial cells were transfected with HA-tagged PTEN prior to incubation with HG. The cell lysates were immunoblotted with phospho-PDGFRβ, HA and PDGFRβ antibodies as indicated (panels A–C). In panel D, the cell lysates were immunoblotted with phospho-p85 PI 3 kinase, HA and p85 antibodies. In panel E, the cell lysates were immunoblotted with phospho-Akt (Ser-473), HA and Akt antibodies. Representative of 3 experiments is shown.

PTEN was originally identified as a dual-specificity phospho-tyrosine/serine/threonine phosphatase [72, 73]. Since PDGFRβ is a tyrosine kinase and undergoes autophosphorylation at tyrosine residues, we hypothesized that PDGFRβ may be a substrate for PTEN. To test this hypothesis, we transfected PTEN into proximal tubular epithelial cells and tested phosphorylation of PDGFRβ. Overexpression of PTEN inhibited high glucose-promoted activating phosphorylation of PDGFRβ at Tyr-857 (Fig 9A and S10A Fig). Consequently, PTEN blocked the PI 3 kinase docking site tyrosine phosphorylation of PDGFRβ (Fig 9B and 9C and S10B and S10C Fig). This deficiency in PDGFRβ phosphorylation by PTEN resulted in inhibition of PI 3 kinase tyrosine phosphorylation and activation of Akt (Fig 9D and 9E and S10D and S10E Fig). Together, our results demonstrate an important role of DJ-1-PTEN axis in activation of PDGFRβ signal transduction to induce proximal tubular epithelial cell hypertrophy and expression of matrix proteins, which contribute to diabetic renal fibrosis.

## Discussion

Although no specific cause of diabetic nephropathy has been identified, poor glycemic control, hypertension, increased oxidative stress and inflammation play significant roles among many other factors [74, 75]. Patients with diabetes show overall growth of the kidney due to tubular hypertrophy [4]. Thus, tubules play a dominant role in the pathogenesis of diabetic kidney disease in which tubular growth contributes to the accumulation of matrix proteins for fibrosis to correlate well with the progression of kidney injury [76–78]. A main feature of fibrosis is the action of high glucose milieu on the proximal tubular cells which set multiple signal transduction pathways leading to tubulointerstitial fibrosis. Although current pharmacological interventions of hypertension and glucose reabsorption in the tubules provide the best therapies for progression of diabetic kidney disease, however, they show no benefit in reversing the injury already occurred [79–81]. Thus, the available therapeutic options are of inadequate efficacy. Therefore, it is necessary to address this unmet need and to understand the complexities of multifaceted signal transduction pathways by high glucose in proximal tubular cells for the development of new therapies for diabetic kidney disease.

**Fig 10:**
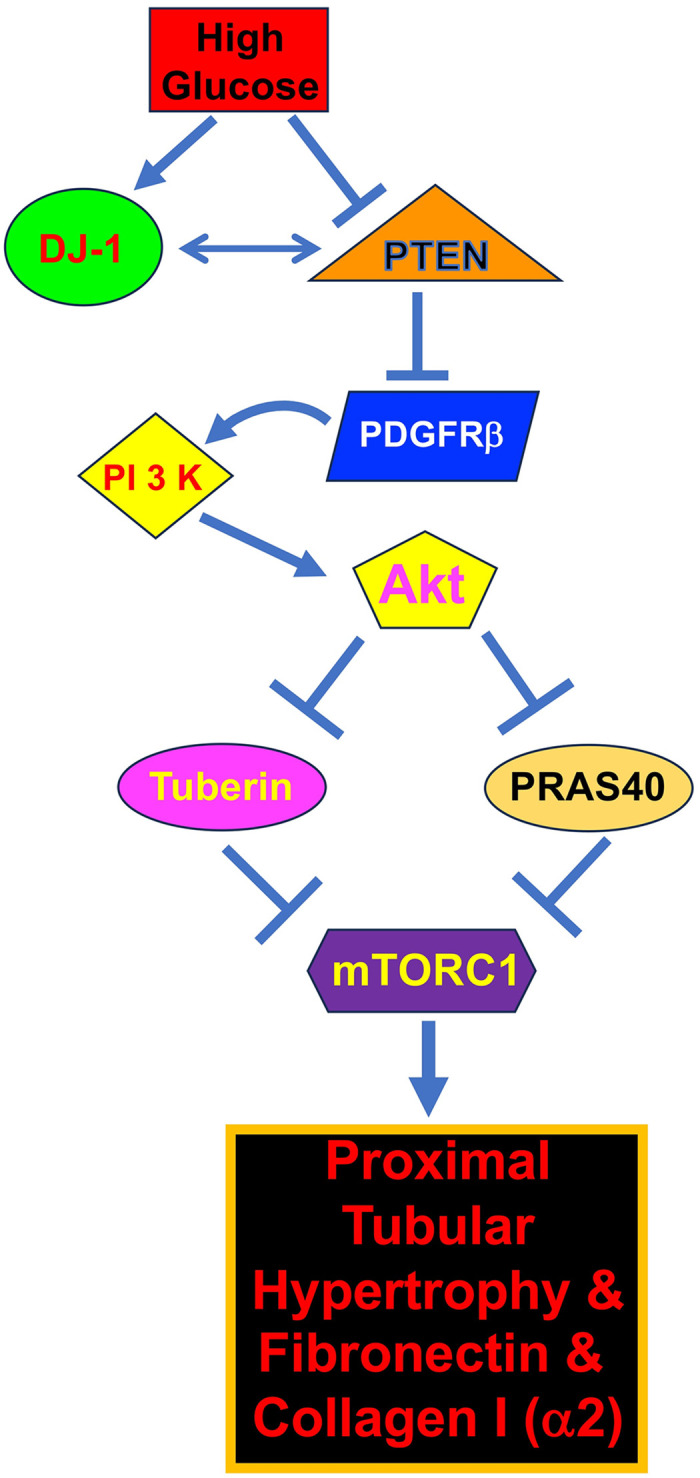
Schematic summarizing the results of the study demonstrating that high glucose-stimulated DJ-1 associates with PTEN resulting in activation of PDGFRβ to activate PI 3 kinase/Akt/mTORC1 signaling for tubular cell hypertrophy and matrix protein expression.

DJ-1 null mice show no abnormalities in the number of dopaminergic neurons although they show hypersensitivity to the dopaminergic toxin MPTP (1-methyl-4-phenyl-1,2,3,6-tetrahydropyridine) that results in dopaminergic dysfunction and motor deficits. These results demonstrate a role of DJ-1 in quenching reactive oxygen species (ROS) [82, 83]. Similar to its function in neurological disorders, ROS play significant role in many other pathologies including cancer, cardiovascular diseases, aging and diabetic kidney disease. The changes in the kidney parenchyma by hyperglycemia results in matrix protein accumulation, leading to albuminuria and renal fibrosis primarily due to increased ROS during the progression of the disease [45, 84, 85]. In fact, we and others have shown that increased Nox4-derived ROS in glomerular and tubular compartments were sufficient for renal hypertrophy and albuminuria in rodent models of diabetic kidney disease [64, 86–90]. However, our observation in this article demonstrates that high glucose increases expression of DJ-1 along with augmented Akt phosphorylation (Fig 10). These results indicate that the ROS quenching activity of DJ-1 found in neuronal cells to protect them from apoptosis may not be functional in proximal tubular epithelial cells.

Since DJ-1 was originally identified as an oncoprotein, its role in multiple cancers has been established [43]. However, in cancer and neurodegenerative diseases, DJ-1 functions in a reciprocal manner [91]. For example, in Parkinson’s disease, neurons undergo apoptosis due to downregulated PI 3 kinase/Akt signaling; but these enzymes act as drivers for tumorigenesis and metastasis [47, 92, 93]. Also, in *Drosophila*, expression of PI 3 kinase catalytic subunit or Akt kinase increased cell size and organ growth [94–96]. Similarly, cardiac-specific expression of constitutively active PI 3 kinase and Akt increased heart size [97, 98]. In support of this observation, PTEN, which inhibits the function of PI 3 kinase, plays an important role in determining cell size in *Drosophila* and in mouse heart [99–102]. Interestingly, *Drosophila* DJ-1A, an ortholog of mammalian DJ-1, antagonizes the suppressive effect of PTEN on eye size [103]. Another study in *Drosophila* where eye size was reduced due to RNAi-mediated knockdown of DJ-1, expression of Akt reversed this phenotype, suggesting that DJ-1 acts upstream of Akt kinase We and others have established a role for PTEN in renal cell hypertrophy and matrix expansion in rodent models of diabetic nephropathy [39, 49, 53, 104]. Also, we previously showed that high glucose-induced activation of Akt kinase and subsequently mTORC1 is necessary for renal cell hypertrophy and matrix protein expression [45, 49, 52, 53]. In the present article, we demonstrate that DJ-1 is necessary for activation of Akt/mTORC1 signaling to drive proximal tubular cell hypertrophy and matrix protein expression by high glucose.

Previous studies have shown increased renal expression of PDGFRβ in patients with diabetes and in rodent kidneys with diabetic kidney disease [19, 67]. Although PDGFRβ in the glomerular mesangial cells plays a significant role in renal development and pathologies, its activation in proximal tubular epithelial cells significantly contribute to the progression of diabetic kidney disease [105]. PDGFRβ contains an extracellular segment followed by a transmembrane and two intracellular tyrosine kinase domains, which are separated by an interkinase domain [106]. Initial trans-autophosphorylation of Tyr-857 in the second kinase domain increases its tyrosine kinase activity towards many tyrosine residues present in the interkinase domain [69]. Two of these residues are Tyr-740 and Tyr-751, which act as docking sites for PI 3 kinase for its own activation and subsequently activating Akt kinase [47, 71]. Thus, phosphorylation of these critical tyrosine residues is necessary for PDGFRβ signal transduction and confers its biological activities. A corollary is that dephosphorylation of PDGFRβ would keep its action in check.

PTEN was originally identified as a dual specificity phosphatase with the ability of dephosphorylating serine/threonine and tyrosine residues [72, 73]. Although PTEN primarily serves as a lipid phosphatase for PIP_3_, the product of PI 3 kinase, PTEN’s crystal structure shows that its catalytic pocket is highly basic [107]. This suggests that PTEN may accommodate acidic substrates such as tyrosine phosphorylated proteins even with weak phosphatase activity [73, 108–110]. Thus, PTEN was shown to dephosphorylate tyrosine phosphorylated focal adhesion kinase (FAK) and Shc to inhibit cell spreading [110, 111]. We have described above that PTEN and DJ-1 plays an antagonistic role in determining cells size in different organs of *Drosophila* [99, 101–103, 112]. However, mice with PTEN deletion in neuronal cells show increase in neuronal stem cells [113]. Similarly, deletion of PTEN in glial cells displays increase in soma size, which resembles the rare autosomal dominant Lhermitte-Duclos syndrome in human in which germ line mutation of PTEN gene is found [114, 115]. Interestingly, in these mice, glial cells showed increased Akt activity without any cell proliferation and the mice died within 29 weeks while glioblastoma was ruled out as cause of death [114]. These results suggest that tumor suppressive function of PTEN observed in various glioblastomas may include targets different from PIP_3_ that regulates Akt activity [72].

Non-covalent interactions of PTEN with multiple proteins regulate its activity. For example, both PREX-2a (PIP_3_-dependent RAC exchanger factor 2a) and SILP1 (shank-interacting protein-like-1) bind to PTEN to inhibit its activity [50, 116]. Similarly, DJ-1 also can form complex with multiple signaling proteins including Raf1, Erb3, SIRT1, androgen receptor and more [43, 117–120]. In the present study, we show that in proximal tubular epithelial cells high glucose-increased DJ-1 forms a complex with PTEN (Fig 10), which may result in inactivation of PTEN phosphatase activity. This notion is supported by our observation that inhibition of DJ-1 blocked high glucose-stimulated tyrosine phosphorylation of PDGFRβ in conjunction with attenuation of mTORC1 activity and proximal tubular cell hypertrophy and, matrix protein expression. Furthermore, our results demonstrate that PTEN dephosphorylates high glucose-induced tyrosine phosphorylation of PDGFRβ. Together, we provide the evidence that PTEN acts as a PDGFRβ phosphatase in proximal tubular epithelial cells and takes part in signaling which contributes to cell injury (Fig 10).

Appropriate growth stimuli that activate receptor tyrosine kinases and nutrients increase mTORC1 activity to enhance macromolecular synthesis. Activation of mTORC1 in mice produce thickening of glomerular basement membrane, podocyte effacement and glomerulosclerosis, features of diabetic kidney disease [121]. Also, overexpression of mTORC1 phosphorylation-deficient mutant of 4EBP-1 in rat induced glomerulosclerosis where the extent of pathology was transgene dose-dependent [122]. However, mTOR also plays an important role in normal physiology and disease resistance [123, 124]. The role of mTOR in organ size came from the studies in *Drosophila* where mutation in the *dTOR* reduced the eye size [125]. Tyrosine kinase-activated mTORC1 requires activation of PI 3 kinase/Akt and inactivation of two negative regulators of mTORC1, tuberin and PRAS40 by Akt [126, 127]. Mutation in *Drosophila* tuberin homolog *gigas* results in increased eye size indicating inactivation of tuberin, which activates mTOR, plays important role in organ size [128]. Similarly, in mouse heart PRAS40 regulates cardiac function [129]. Furthermore, inhibition of mTORC1 by expressing PRAS40 *in vivo* in cardiomyocytes ameliorated hypertrophic growth and preserved cardiac function in mice with diabetic cardiomyopathy [130]. These results indicate that tuberin as well as PRAS40 play roles in inactivating mTORC1 to ameliorate cell hypertrophy. In the present study, we provide the first evidence that high glucose uses the oncoprotein DJ-1 to regulate phosphorylation and inactivation of tuberin and PRAS40 resulting in mTORC1 activity.

Using rapamycin, we and others have previously reported a significant role of mTORC1 in renal hypertrophy and matrix protein expansion during the progression of diabetic kidney disease [61, 63, 64, 121]. We also showed requirement of mTORC1 for renal cell hypertrophy and expression of different matrix proteins [40, 49, 52, 53, 57, 131, 132]. However, chronic rapamycin treatment produced insulin resistance in rat and glucose intolerance in mice [133–135]. Also, inhibition of mTORC1 by rapamycin can be detrimental as it produces proteinuria in a rat model of chronic kidney disease [136]. In fact, mTOR plays a significant role in normal physiology as the deletion of this kinase in mice is embryonically lethal [137]. More recently in a mouse model where mTORC1 was specifically inactivated in proximal tubular epithelial cells, the mouse showed progressive tubular fibrosis along with glucosuria, aminoaciduria, phosphaturia, low molecular weight proteinuria and albuminuria [138, 139]. Similar results occur in rapamycin therapy of patients with renal transplantation and allograft nephropathy [140, 141]. Also, rapamycin induced tubular damage and proteinuria in renal transplant patients [142]. Thus, although rapamycin-mediated inhibition of mTORC1 may demonstrate ameliorative effect towards pathologies of diabetic nephropathy, its efficacy may be compromised due to the adverse effects. Thus, direct targeting of mTORC1 may be deleterious. Therefore, it is important to identify alternative signaling molecules, which converge into the mTORC1 pathway as its inhibition is protective for diabetic kidney disease. In the present study, we identified a novel signaling pathway where the oncoprotein DJ-1 acts as a high glucose sensitive protein, which regulates phosphorylation/inactivation of tuberin and PRAS40 by Akt kinase through interaction with PTEN. Thus, we showed that DJ-1 and PTEN act on PDGFRβ to initiate a signaling cascade to regulate high glucose-induced proximal tubular cell hypertrophy and expression of the matrix proteins fibronectin and collagen I (α2) (Fig 10). Thus, we propose to consider targeting DJ-1-PTEN axis to block hyperactive mTORC1 found in renal pathologies of diabetic kidney disease.

## Supporting information

S1 Fig Quantification of Fig 1A–1D. (A and B) Ratio of DJ-1 to actin. Mean ± SD of 3–5 experiments is shown. *p < 0.001 vs 0 hour. (C and D) Ratio of p-Akt to Akt. Mean ± SD of 3–4 independent experiments is shown. *p < 0.001 vs 0 hour.
(PDF)

S2 Fig Quantification of Fig 2C–2H. (A and B) Ratio of PTEN to DJ-1 for Fig 2C and 2D. (C and E) Ratio of DJ-1 to actin for Fig 2E and 2G. (D and F) Ratio of PTEN to actin for Fig 2F and 2H (F). Mean ± SD of 3 independent experiments is shown. *p < 0.001–0.01 vs NG.
(PDF)

S3 Fig Effect of different concentration of glucose on association of DJ-1 with PTEN. Proximal tubular epithelial cells were incubated with indicated concentrations of glucose for 24 hours. (A) Cell lysates were immunoprecipitated with DJ-1 antibody followed by immunoblotting with PTEN and DJ-1 antibodies. (C) Cell lysates were immunoprecipitated with PTEN antibody followed by immunoblotting with DJ-1 and PTEN antibodies. The bottom panels show actin immunoblotting of cell lysates. (B and D) Ratio of PTEN to actin (B) and ratio of DJ-1 to actin (D) are shown. Mean ± SD of three independent experiments is shown; *p < 0.0005–0.001 vs 0 mM or 5 mM glucose.
(PDF)

S4 Fig Quantification of Fig 3. (A and E) Ratio of p-Akt to Akt. (B and F) Ratio of p-GSK3β to GSK3β. (C and G) Ratio of p-Tuberin to Tuberin. (D and H) Ratio of p-PRAS40 to PRAS40. Mean ± SD of three independent experiments is shown. *p < 0.00–0.05 vs NG; **p < 0.001–0.01 bs HG.
(PDF)

S5 Fig Quantification of Fig 4. (A and D) Ratio of p-4EBP-1 to 4EBP-1 for Fig 4A and 4D, respectively. (B and E) Ratio of p-S6 kinase to S6 kinase for Fig 4B and 4E, respectively. (C and F) Ratio of p-mTOR to mTOR for Fig 4C and 4F, respectively. Mean ± SD of three independent experiments. *p < 0.001–0.05; **p < 0.001–0.05.
(PDF)

S6 Fig Quantification of Fig 6. (A and C) Ratio of fibronectin to actin. (B and D) Ratio of collagen I (α2) to actin. Mean ± SD of three experiments is shown. *p < 0.01–0.05 vs NG; **p < 0.01–0.05 vs HG.
(PDF)

S7 Fig (A and B) Quantification of Fig 7C and 7D. Ratio of fibronectin (A) and collagen I (α2) (B) to actin, respectively. Mean ± SD of four experiments is shown. *p < 0.001 vs NG; **p < 0.001 vs HG. (C) Downregulation of raptor inhibits DJ-1-induced mTORC1 activity as judged by phosphorylation of S6 kinase. Proximal tubular epithelial cells were transfected with shRNA plasmid for raptor or vector or FLAG DJ-1. The cell lysates were immunoblotted with indicated antibodies. (D) Quantification of data in C. Mean ± SD of three independent experiments is shown. *p < 0.01 vs control; **p < 0. 01vs DJ-1. (E and F) Quantification of Fig 7G and 7H. Ratio of fibronectin (E) and collagen I (α2) (F) to actin, respectively. Mean ± SD of four experiments is shown. *p < 0.001 vs control; **p < 0.001 vs FLAG DJ-1.
(PDF)

S8 Fig High glucose stimulates activating phosphorylation of PDGFRβ in a sustained manner. (A and C) Proximal tubular epithelial cells were incubated with 25 mM glucose for indicated periods of time. The cell lysates were immunoblotted with indicated antibodies. (B and D) Quantification of A and C, respectively. Mean ± SD of four experiments is shown. *p < 0.05 vs 0 hour.
(PDF)

S9 Fig Quantification of Fig 8. (A and E) Ratio of p-PDGFRβ (Tyr-857) to PDGFRβ. (B and F) Ratio of p-PDGFRβ (Tyr-740) to PDGFRβ. (C and G) Ratio of p-PDGFRβ (Tyr-751) to PDGFRβ. (D and H) Ratio of p-p85 (Tyr-458) to p85. Mean ± SD of three experiments is shown. *p < 0.00-.01 vs NG; **p < 0.001 vs HG.
(PDF)

S10 Fig Quantification of Fig 9. (A–C) Ratio of p-PDGFRβ (Tyr-857) (A), p-PDGFRβ (Tyr-740) (B) and p-PDGFRβ (Tyr-751) (C) to PDGFRβ. (D) Ratio of p-p85 to p85. (E) Ratio of p-Akt to Akt. Mean ± SD of three experiments is shown. *p < 0.001–0.01 vs NG; **p < 0.001–0.01 vs HG.
(PDF)

S1 Raw images (PDF)
