# Supplementary figures and images for: High glucose couples DJ-1 with PTEN to activate PDGFRβ for renal proximal tubular cell injury
Source: PLoS One. 2025 Jan 6;20(1):e0311828. doi: 10.1371/journal.pone.0311828 (PMC11703087; doi:10.1371/journal.pone.0311828)

**A**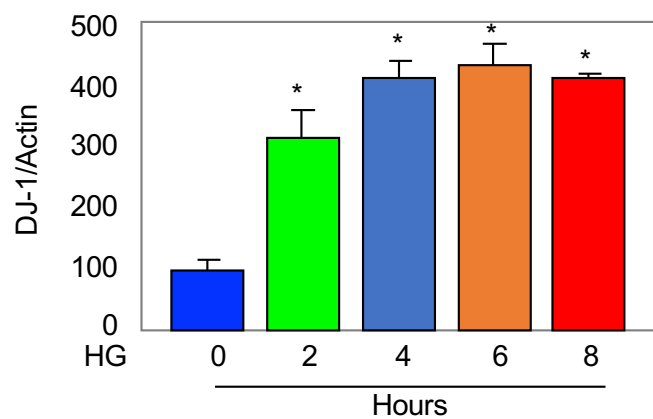**B**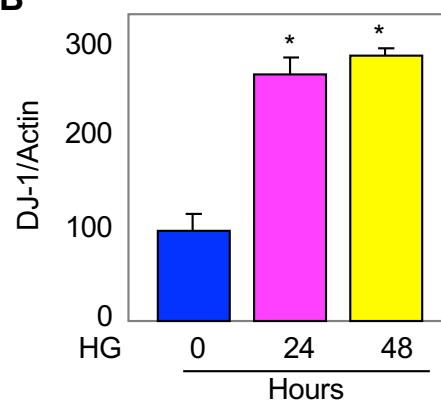**C**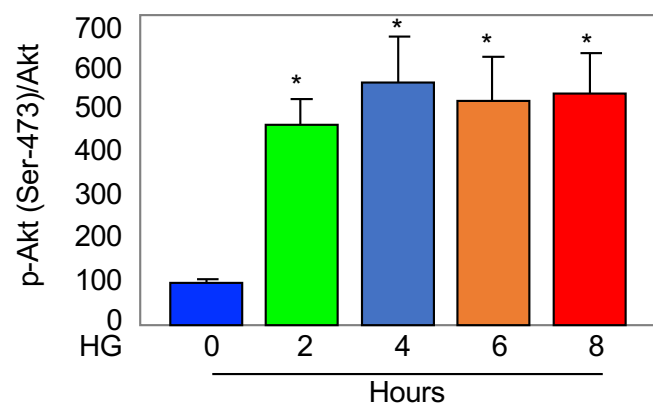**D**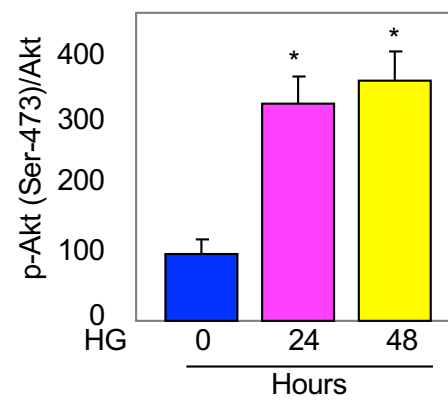

S1 Fig.

Supplement: S1 Fig — (A and B) Ratio of DJ-1 to actin. Mean ± SD of 3–5 experiments is shown. *p < 0.001 vs 0 hour. (C and D) Ratio of p-Akt to Akt. Mean ± SD of 3–4 independent experiments is shown. *p < 0.001 vs 0 hour. (PDF) [file pone.0311828.s001.pdf]

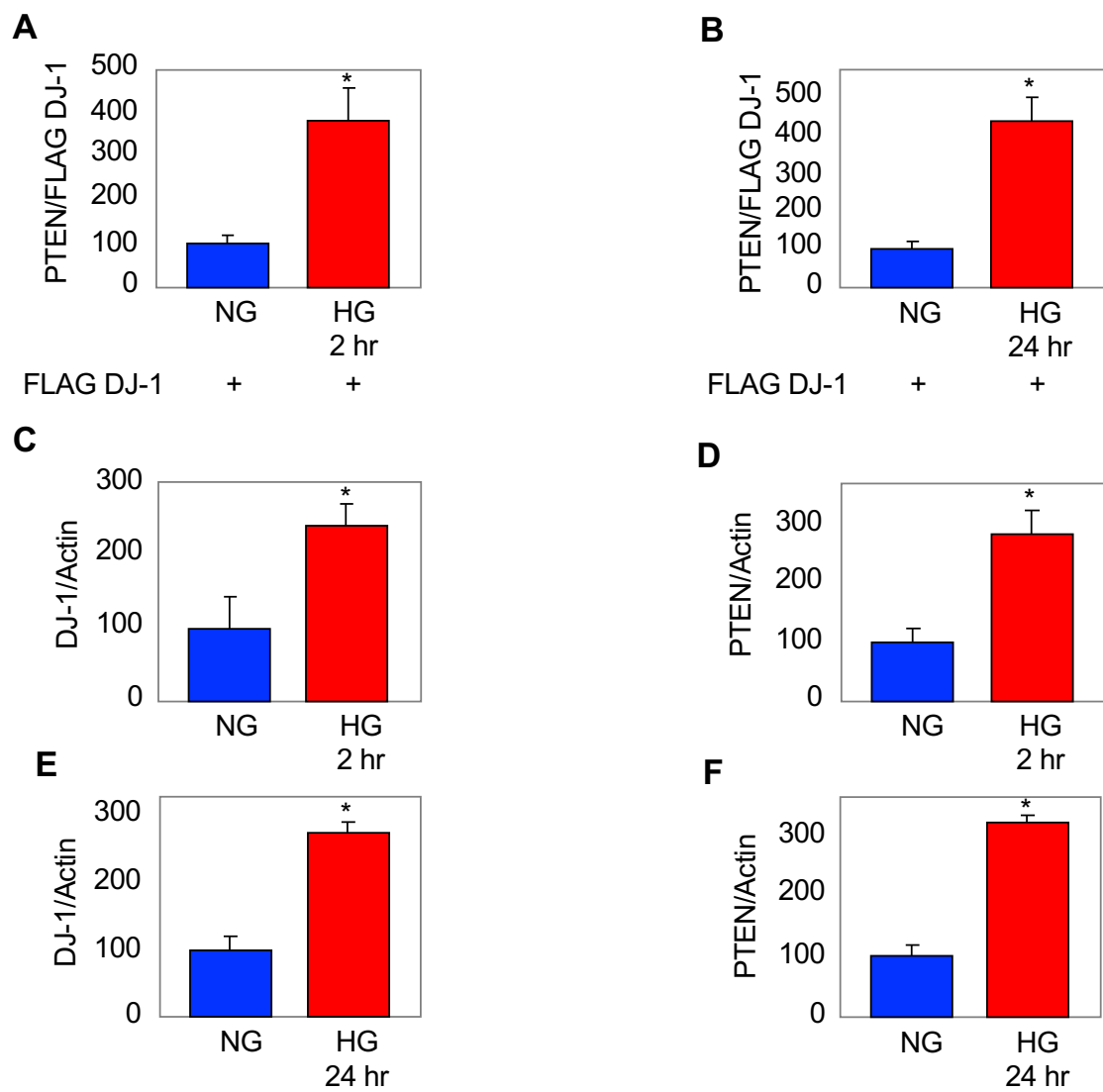

S2 Fig.

Supplement: S2 Fig — (A and B) Ratio of PTEN to DJ-1 for Fig 2C and 2D. (C and E) Ratio of DJ-1 to actin for Fig 2E and 2G. (D and F) Ratio of PTEN to actin for Fig 2F and 2H (F). Mean ± SD of 3 independent experiments is shown. *p < 0.001–0.01 vs NG. (PDF) [file pone.0311828.s002.pdf]

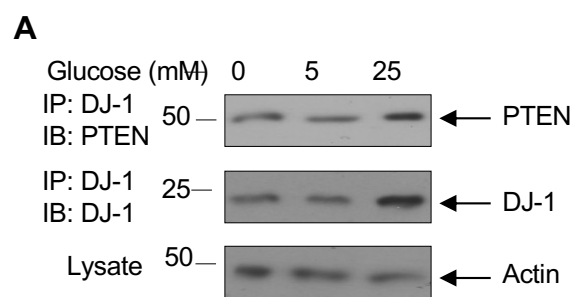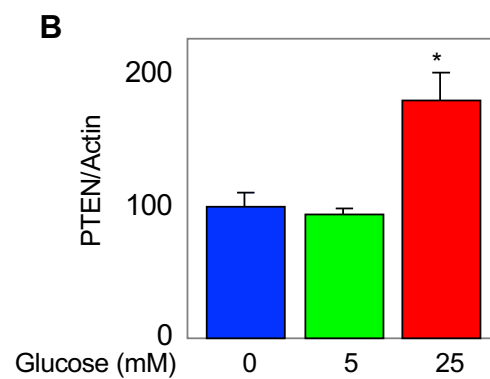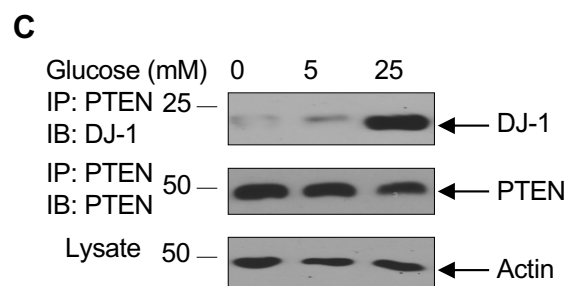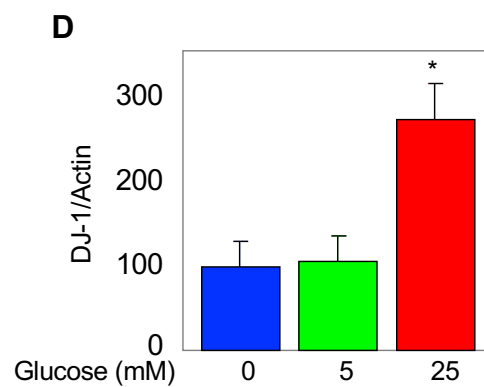

Supplement: S3 Fig — Proximal tubular epithelial cells were incubated with indicated concentrations of glucose for 24 hours. (A) Cell lysates were immunoprecipitated with DJ-1 antibody followed by immunoblotting with PTEN and DJ-1 antibodies. (C) Cell lysates were immunoprecipitated with PTEN antibody followed by immunoblotting with DJ-1 and PTEN antibodies. The bottom panels show actin immunoblotting of cell lysates. (B and D) Ratio of PTEN to actin (B) and ratio of DJ-1 to actin (D) are shown. Mean ± SD of three independent experiments is shown; *p < 0.0005–0.001 vs 0 mM or 5 mM glucose. (PDF) [file pone.0311828.s003.pdf]

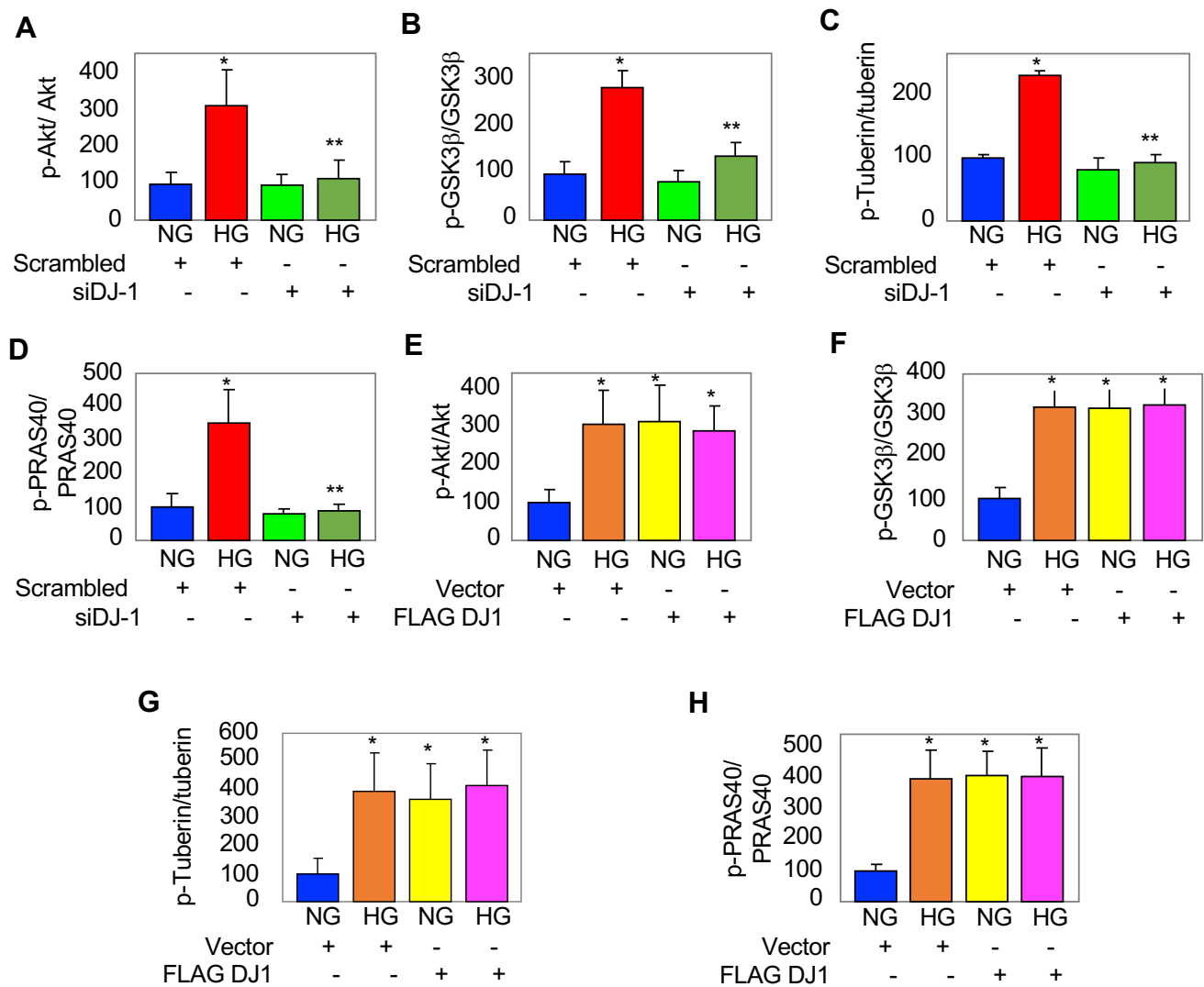

S4 Fig.

Supplement: S4 Fig — (A and E) Ratio of p-Akt to Akt. (B and F) Ratio of p-GSK3β to GSK3β. (C and G) Ratio of p-Tuberin to Tuberin. (D and H) Ratio of p-PRAS40 to PRAS40. Mean ± SD of three independent experiments is shown. *p < 0.00–0.05 vs NG; **p < 0.001–0.01 bs HG. (PDF) [file pone.0311828.s004.pdf]

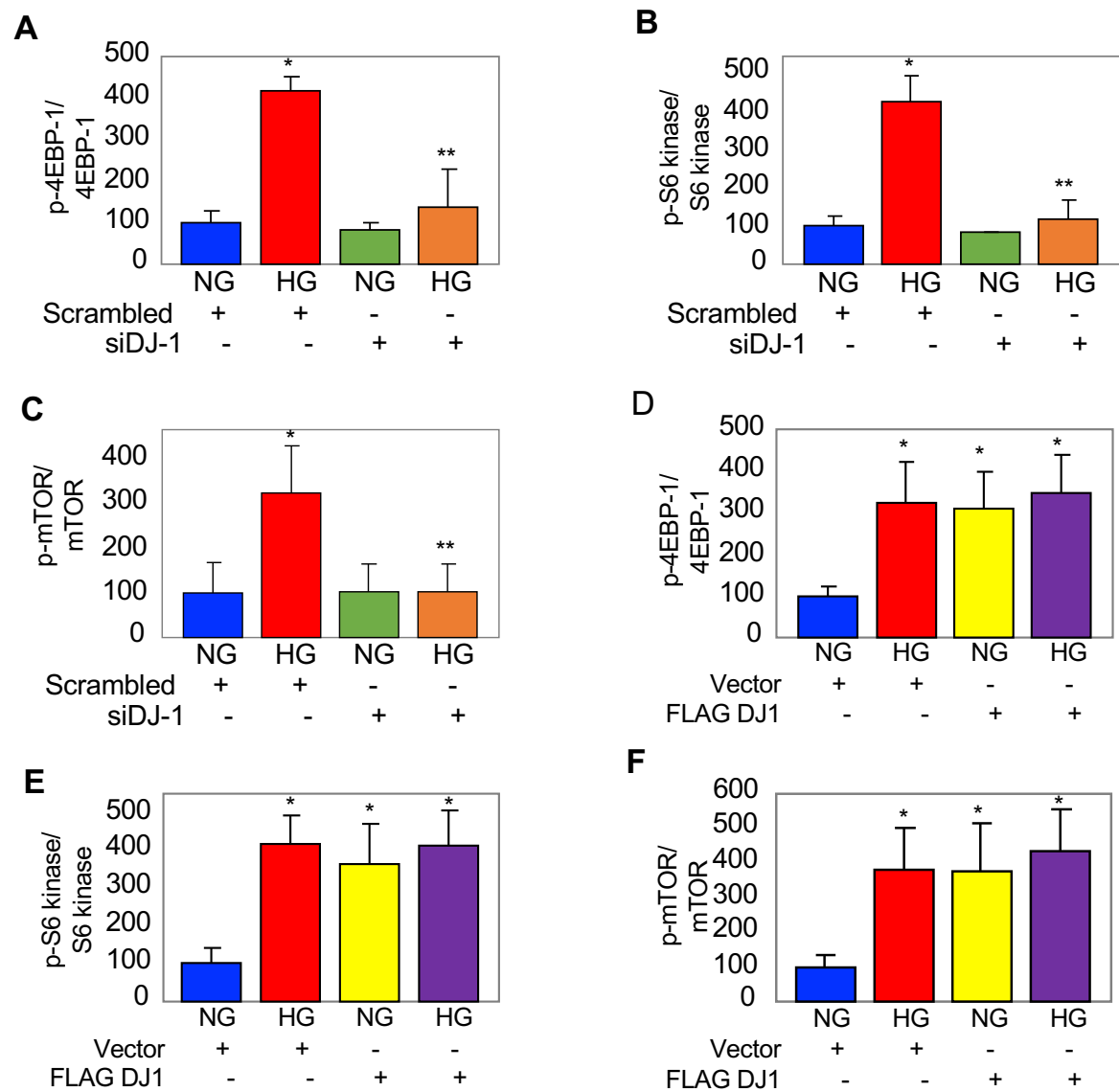

S5 Fig.

Supplement: S5 Fig — (A and D) Ratio of p-4EBP-1 to 4EBP-1 for Fig 4A and 4D, respectively. (B and E) Ratio of p-S6 kinase to S6 kinase for Fig 4B and 4E, respectively. (C and F) Ratio of p-mTOR to mTOR for Fig 4C and 4F, respectively. Mean ± SD of three independent experiments. *p < 0.001–0.05; **p < 0.001–0.05. (PDF) [file pone.0311828.s005.pdf]

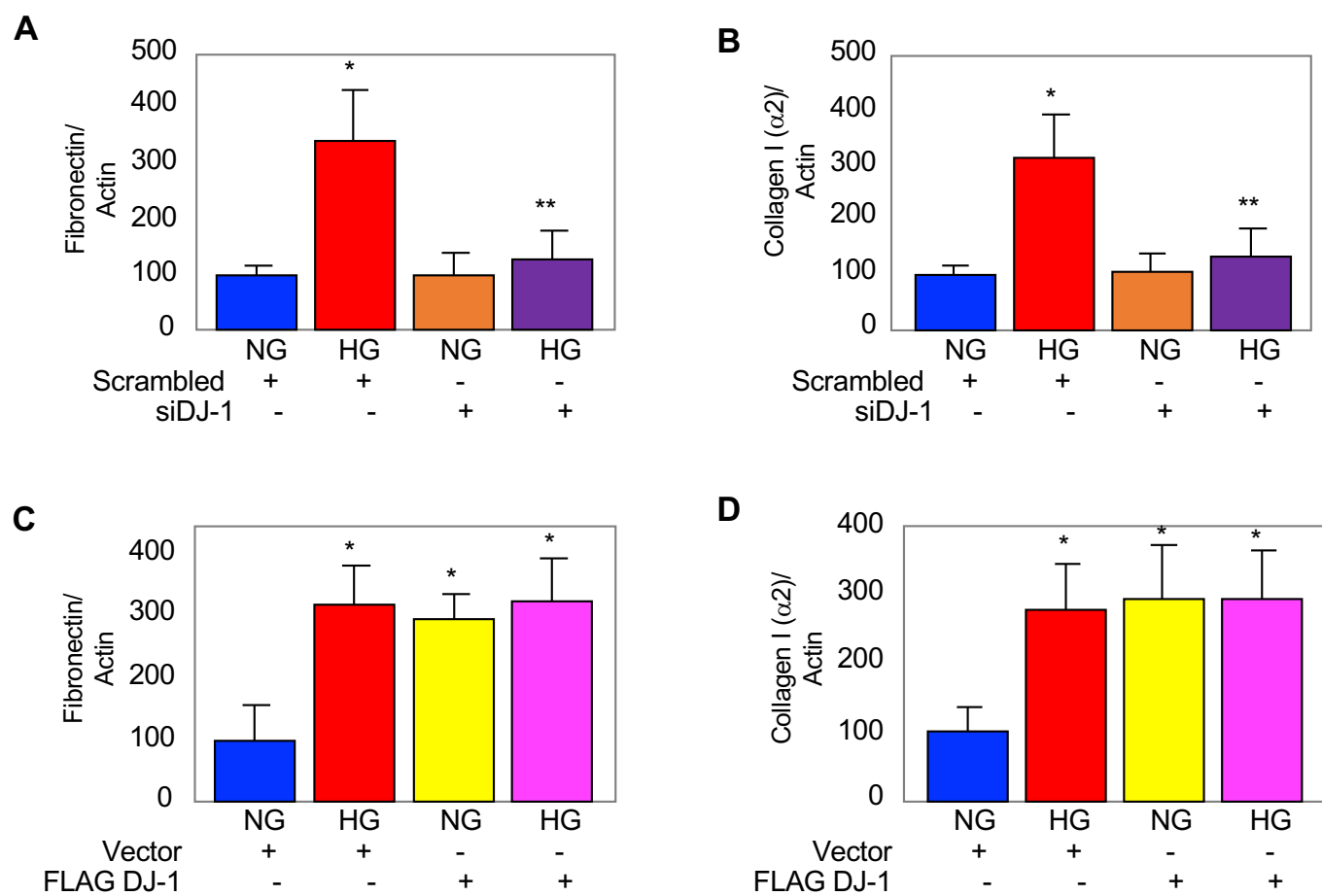

S6 Fig.

Supplement: S6 Fig — (A and C) Ratio of fibronectin to actin. (B and D) Ratio of collagen I (α2) to actin. Mean ± SD of three experiments is shown. *p < 0.01–0.05 vs NG; **p < 0.01–0.05 vs HG. (PDF) [file pone.0311828.s006.pdf]

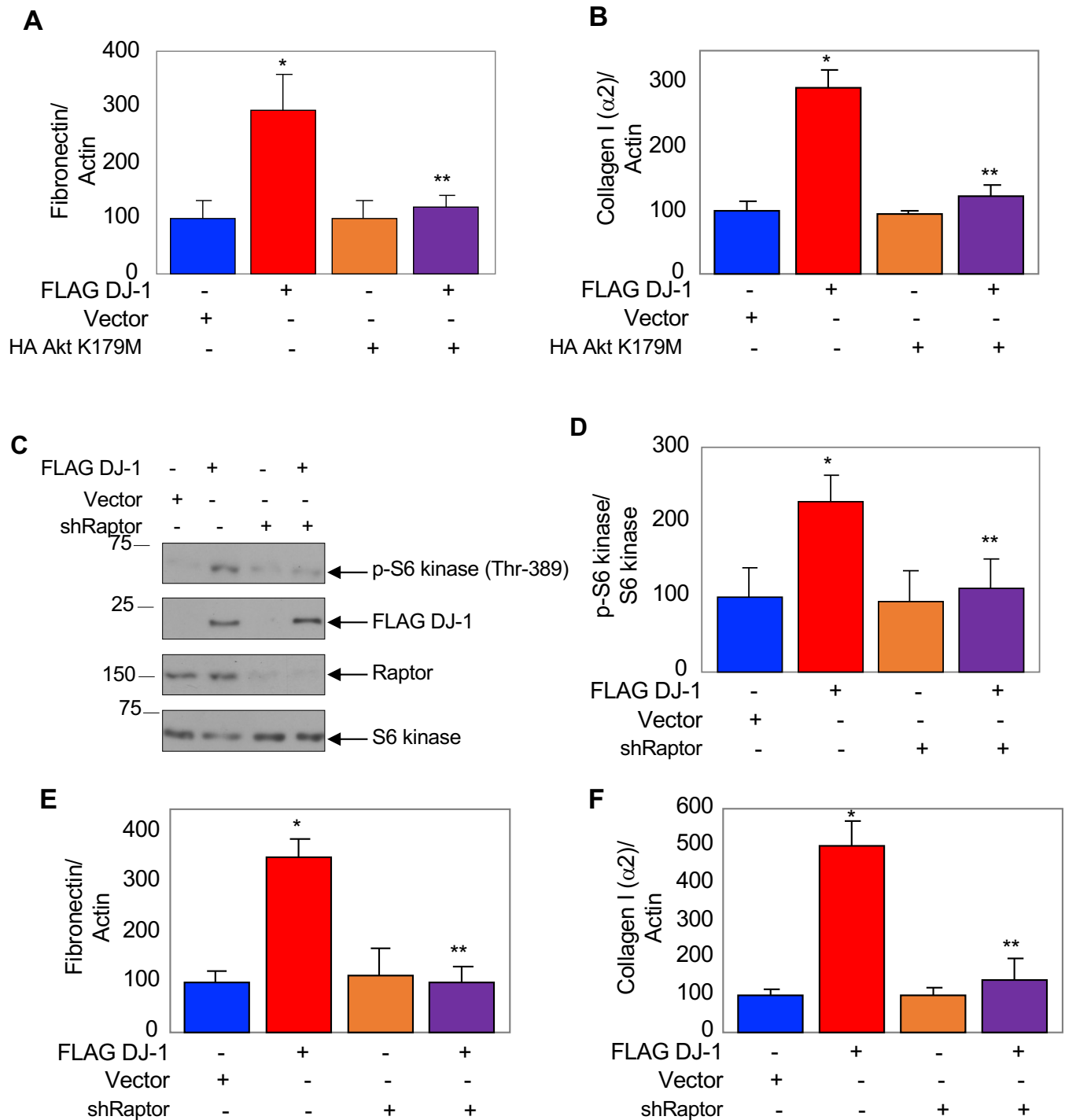

Supplement: S7 Fig — (A and B) Quantification of Fig 7C and 7D. Ratio of fibronectin (A) and collagen I (α2) (B) to actin, respectively. Mean ± SD of four experiments is shown. *p < 0.001 vs NG; **p < 0.001 vs HG. (C) Downregulation of raptor inhibits DJ-1-induced mTORC1 activity as judged by phosphorylation of S6 kinase. Proximal tubular epithelial cells were transfected with shRNA plasmid for raptor or vector or FLAG DJ-1. The cell lysates were immunoblotted with indicated antibodies. (D) Quantification of data in C. Mean ± SD of three independent experiments is shown. *p < 0.01 vs control; **p < 0. 01vs DJ-1. (E and F) Quantification of Fig 7G and 7H. Ratio of fibronectin (E) and collagen I (α2) (F) to actin, respectively. Mean ± SD of four experiments is shown. *p < 0.001 vs control; **p < 0.001 vs FLAG DJ-1. (PDF) [file pone.0311828.s007.pdf]

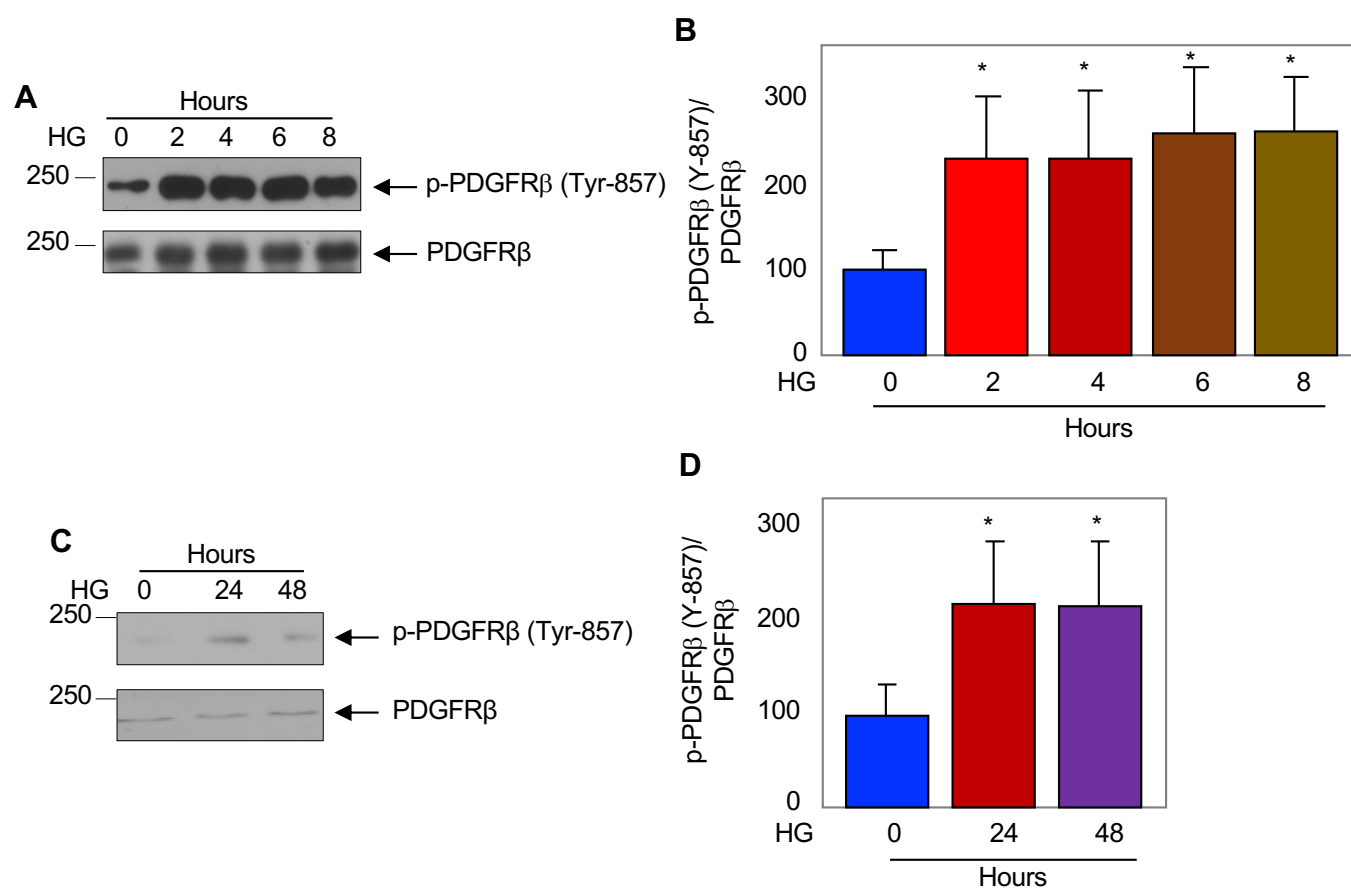

S8 Fig.

Supplement: S8 Fig — (A and C) Proximal tubular epithelial cells were incubated with 25 mM glucose for indicated periods of time. The cell lysates were immunoblotted with indicated antibodies. (B and D) Quantification of A and C, respectively. Mean ± SD of four experiments is shown. *p < 0.05 vs 0 hour. (PDF) [file pone.0311828.s008.pdf]

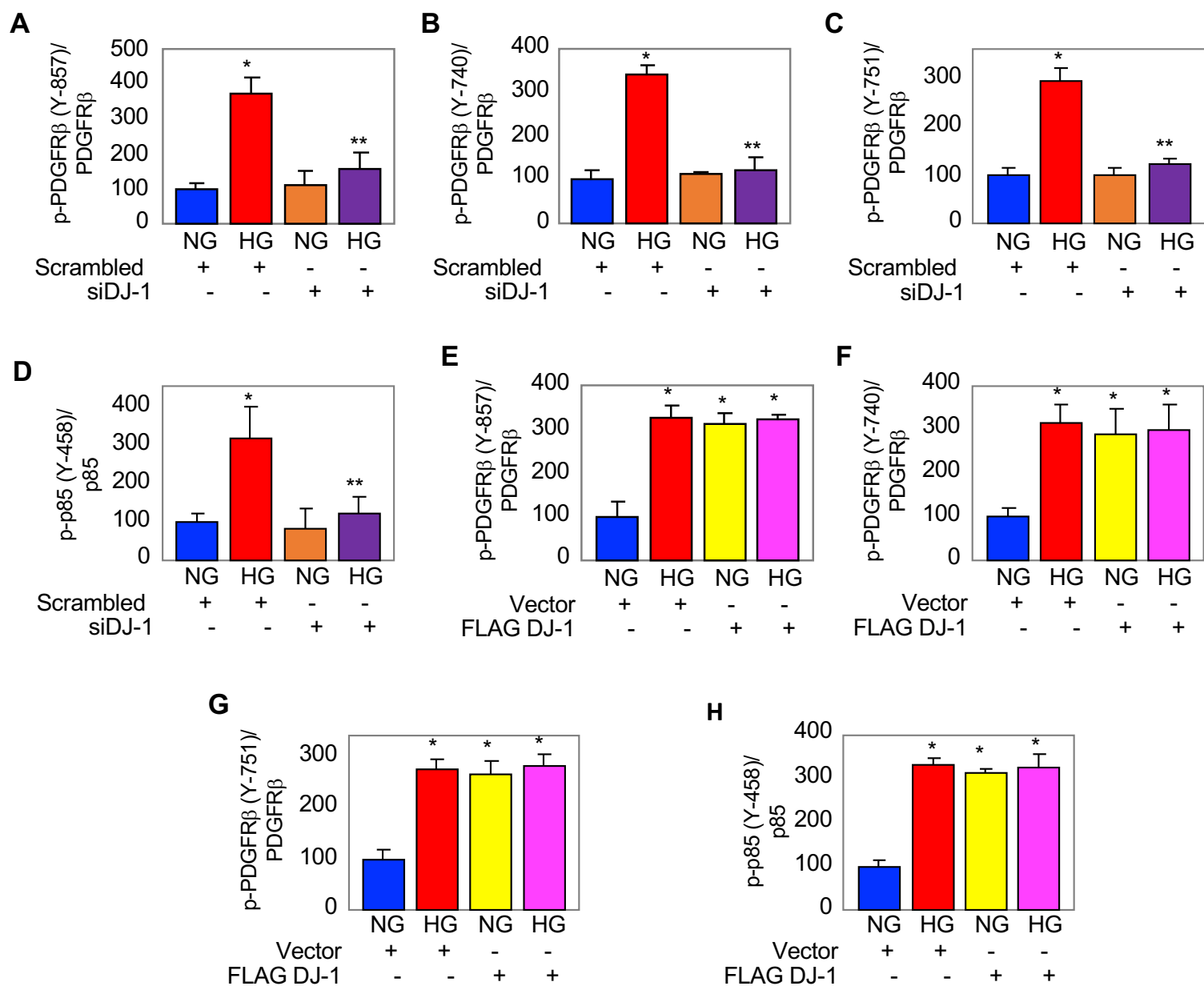

S9 Fig.

Supplement: S9 Fig — (A and E) Ratio of p-PDGFRβ (Tyr-857) to PDGFRβ. (B and F) Ratio of p-PDGFRβ (Tyr-740) to PDGFRβ. (C and G) Ratio of p-PDGFRβ (Tyr-751) to PDGFRβ. (D and H) Ratio of p-p85 (Tyr-458) to p85. Mean ± SD of three experiments is shown. *p < 0.00-.01 vs NG; **p < 0.001 vs HG. (PDF) [file pone.0311828.s009.pdf]

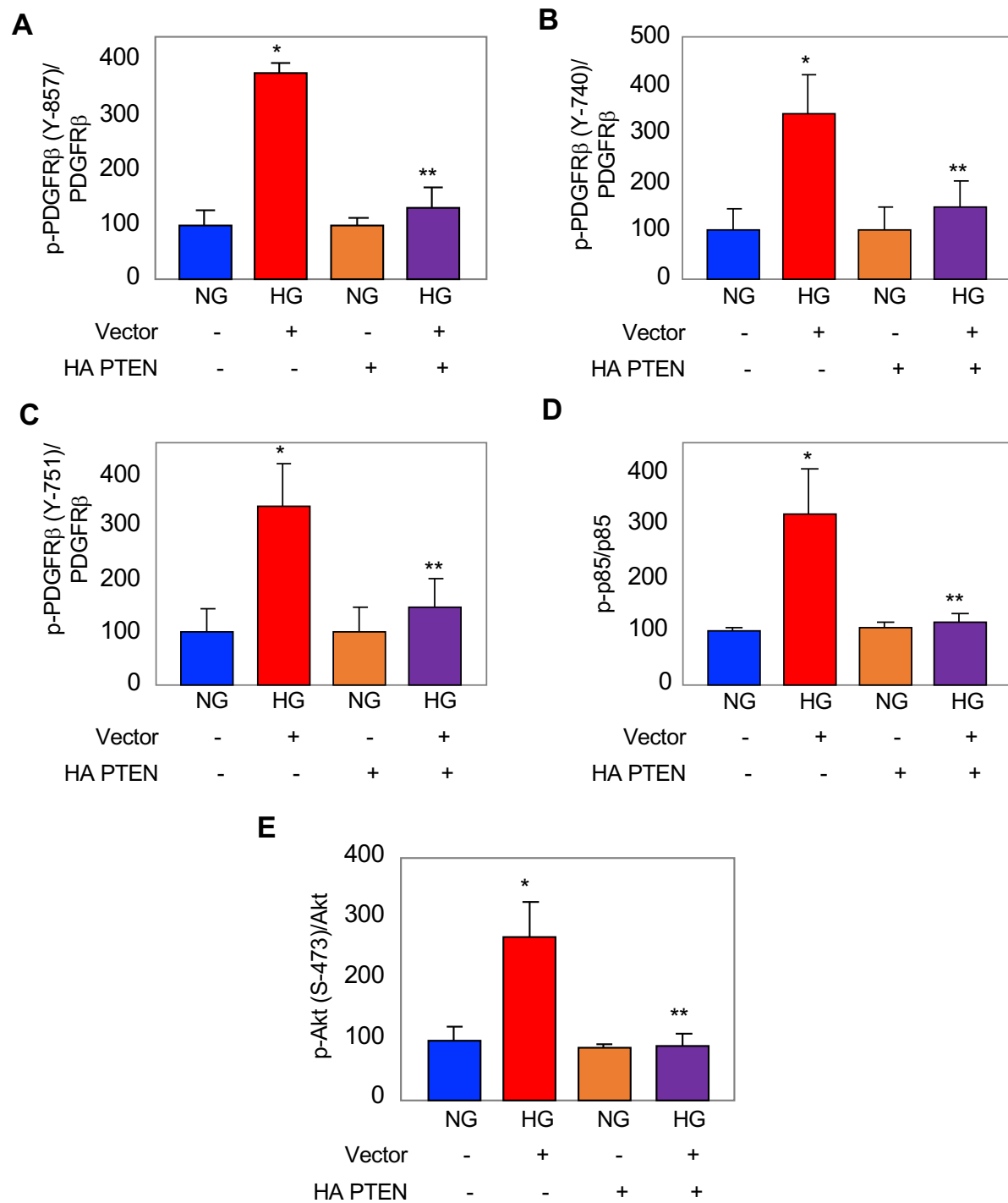

S10 Fig.

Supplement: S10 Fig — (A–C) Ratio of p-PDGFRβ (Tyr-857) (A), p-PDGFRβ (Tyr-740) (B) and p-PDGFRβ (Tyr-751) (C) to PDGFRβ. (D) Ratio of p-p85 to p85. (E) Ratio of p-Akt to Akt. Mean ± SD of three experiments is shown. *p < 0.001–0.01 vs NG; **p < 0.001–0.01 vs HG. (PDF) [file pone.0311828.s010.pdf]
