# Supplementary material for: High glucose couples DJ-1 with PTEN to activate PDGFRβ for renal proximal tubular cell injury
Source: PLoS One. 2025 Jan 6;20(1):e0311828. doi: 10.1371/journal.pone.0311828 (PMC11703087; doi:10.1371/journal.pone.0311828)
Supplement: S1 Raw images — (PDF) [file pone.0311828.s011.pdf]

Fig. 1A

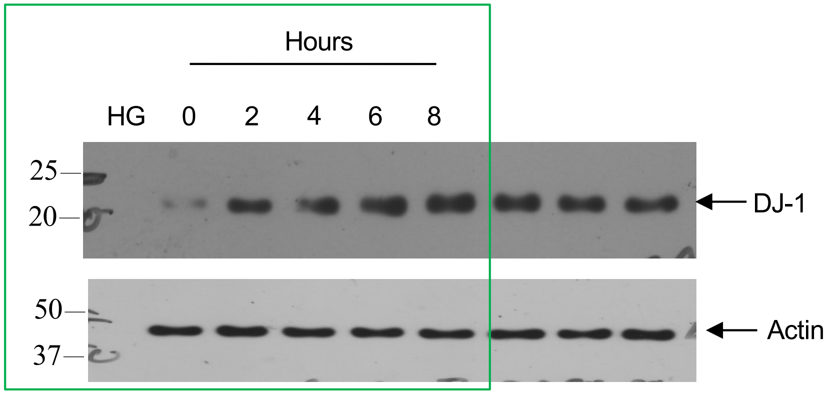

Fig. 1C

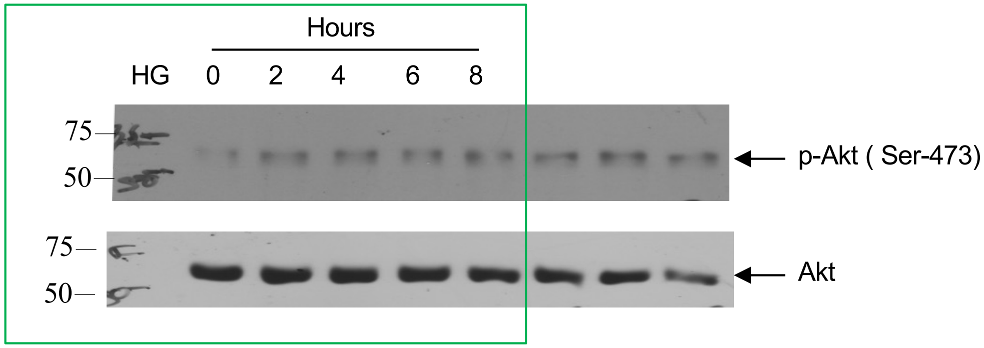

Fig. 1B

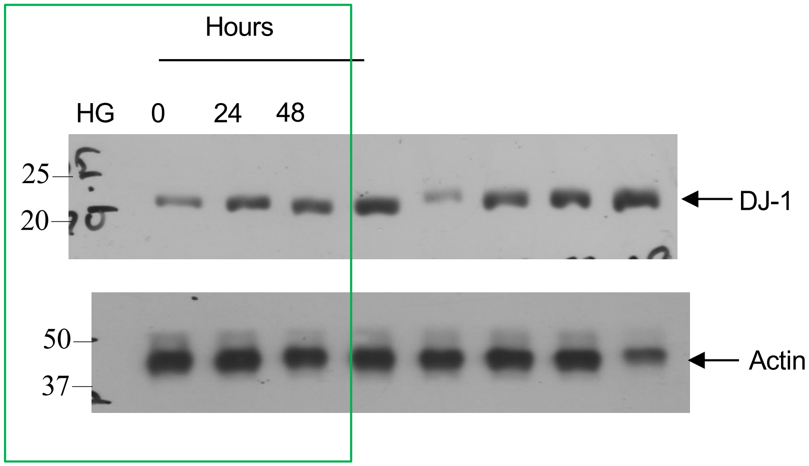

Fig. 1D

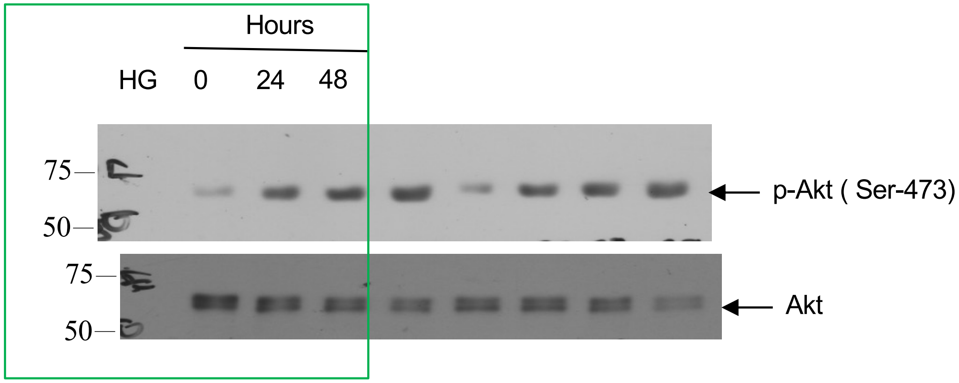

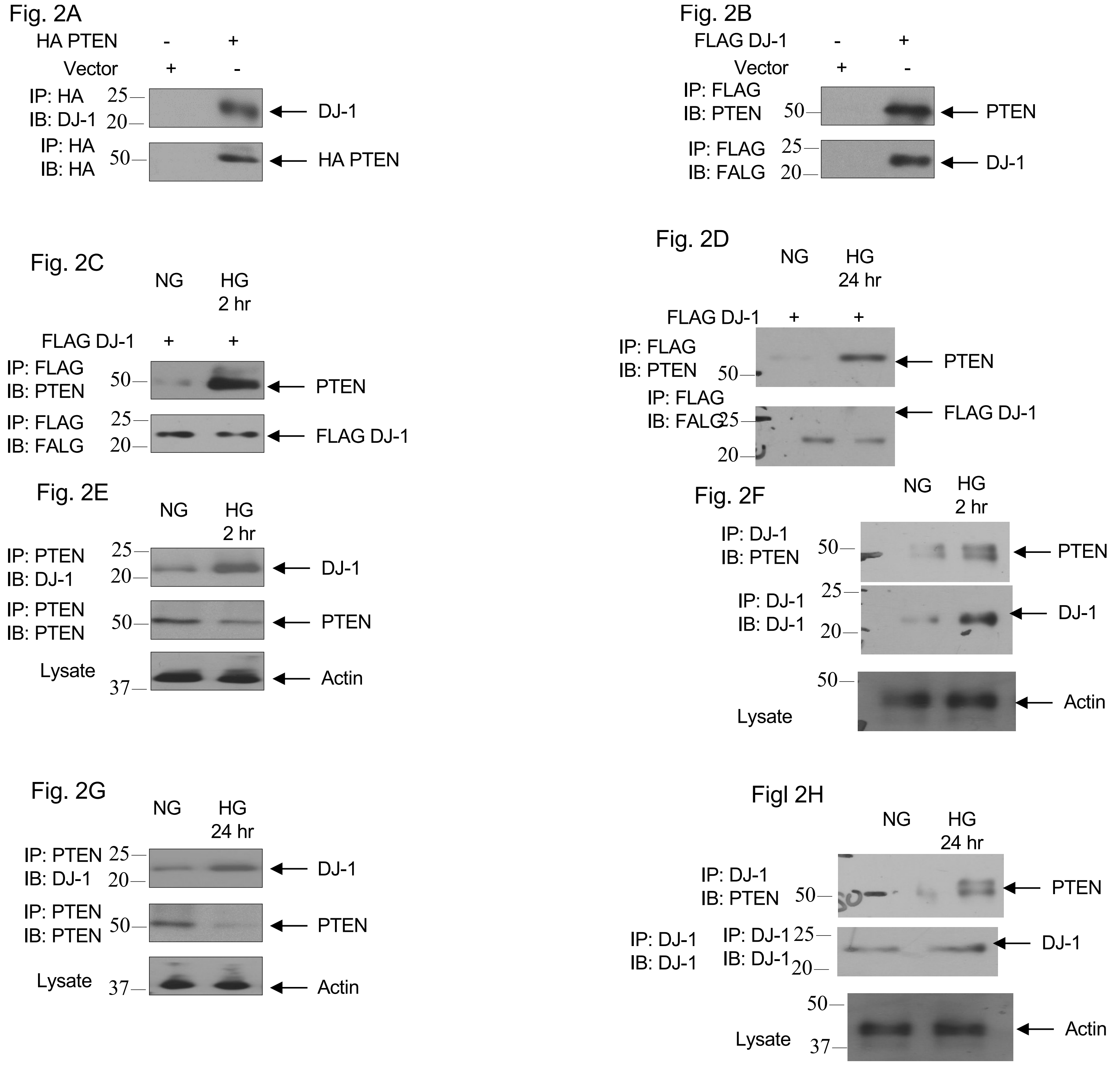
Fig. 2A
HA PTEN - +
Vector + -
IP: HA
IB: DJ-1
25
20
DJ-1
IP: HA
IB: HA
50
HA PTEN
Fig. 2B
FLAG DJ-1 - +
Vector + -
IP: FLAG
IB: PTEN
50
PTEN
IP: FLAG
IB: FALG
25
20
DJ-1
Fig. 2C
NG
HG
2 hr
FLAG DJ-1 + +
IP: FLAG
IB: PTEN
50
PTEN
IP: FLAG
IB: FALG
25
20
FLAG DJ-1
Fig. 2D
NG
HG
24 hr
FLAG DJ-1 + +
IP: FLAG
IB: PTEN
50
PTEN
IP: FLAG
IB: FALG
25
20
FLAG DJ-1
Fig. 2E
NG
HG
2 hr
IP: PTEN
IB: DJ-1
25
20
DJ-1
IP: PTEN
IB: PTEN
50
PTEN
Lysate
37
Actin
Fig. 2F
NG
HG
2 hr
IP: DJ-1
IB: PTEN
50
PTEN
IP: DJ-1
IB: DJ-1
25
20
DJ-1
50
Lysate
Actin
Fig. 2G
NG
HG
24 hr
IP: PTEN
IB: DJ-1
25
20
DJ-1
IP: PTEN
IB: PTEN
50
PTEN
Lysate
37
Actin
Figl 2H
NG
HG
24 hr
IP: DJ-1
IB: PTEN
50
PTEN
IP: DJ-1
IB: DJ-1
IP: DJ-1
IB: DJ-1
25
20
DJ-1
50
Lysate
37
Actin

Fig. 3A

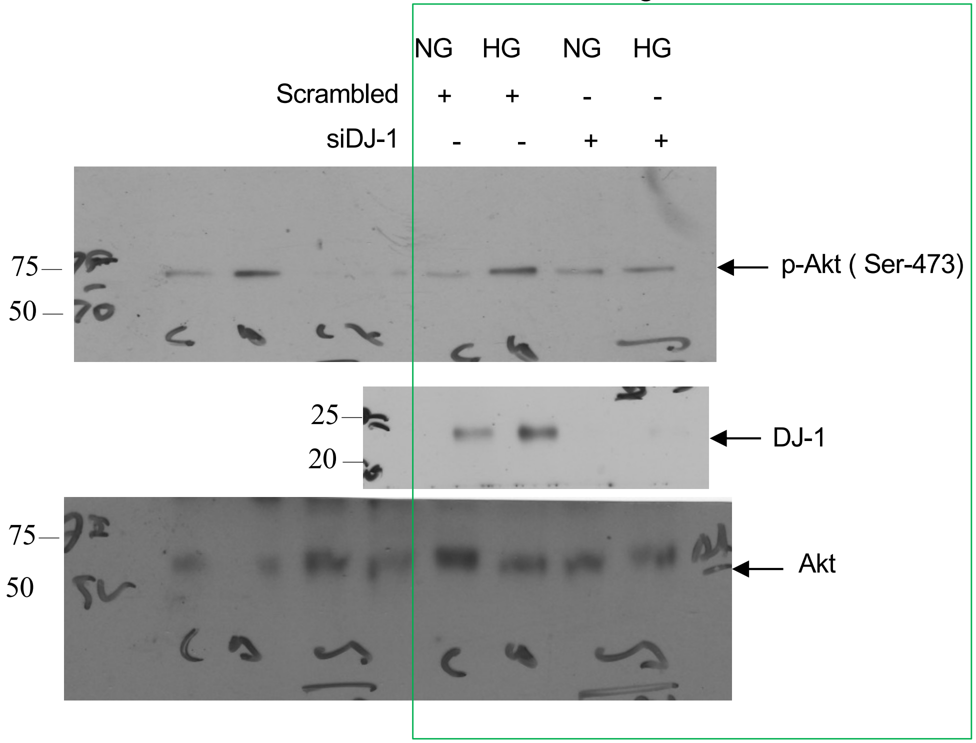

Fig. 3C

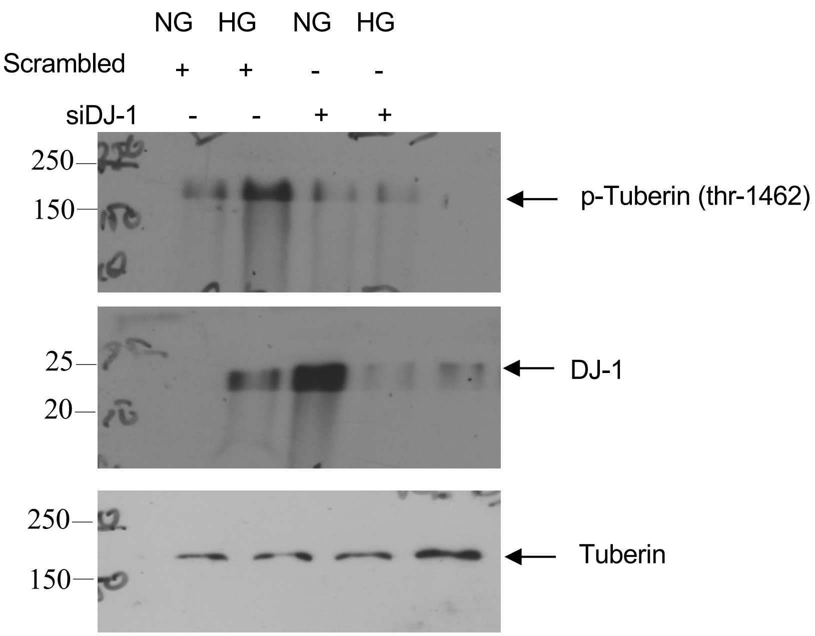

Fig. 3D

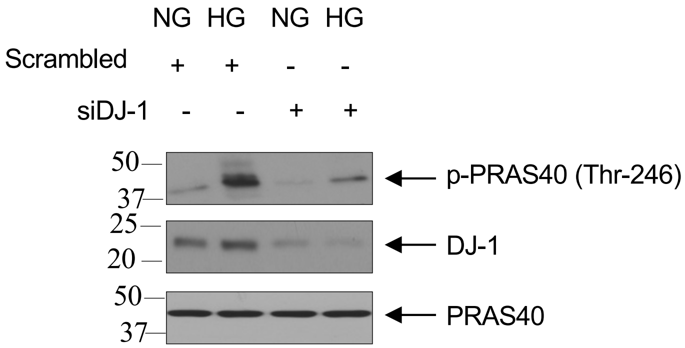

Fig. 3B

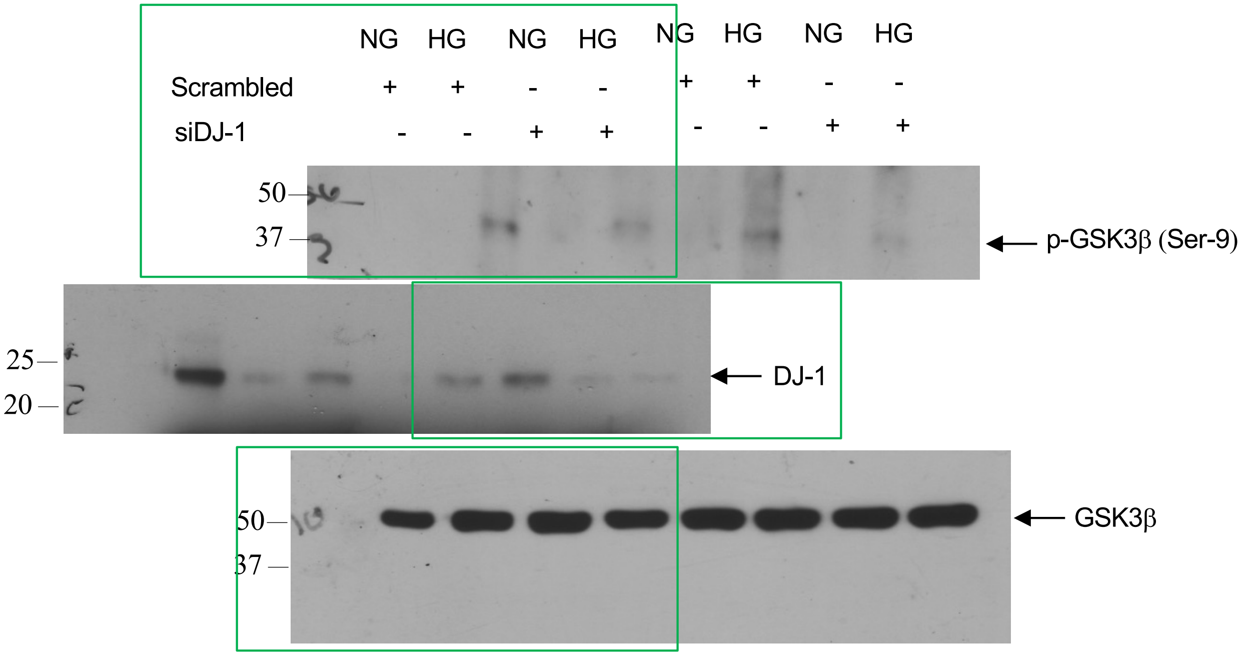

Fig. 3E

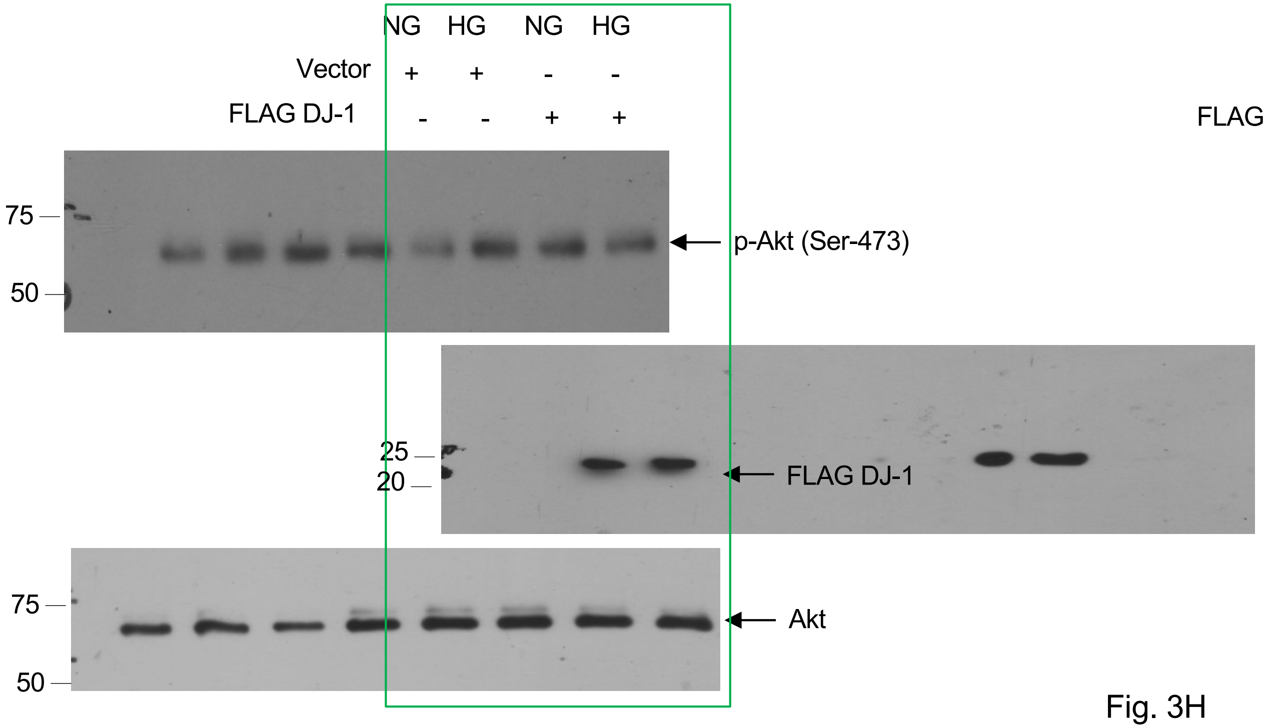

Fig. 3F

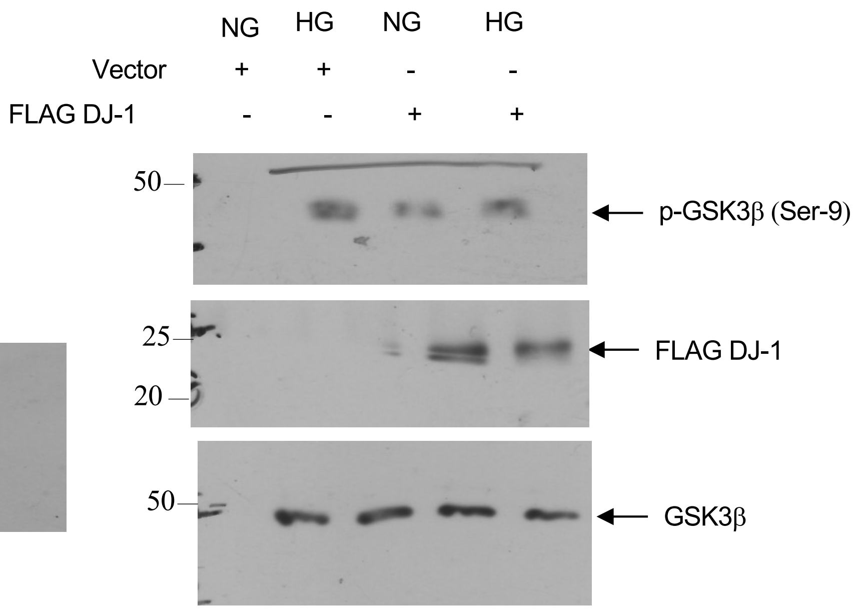

Fig. 3H

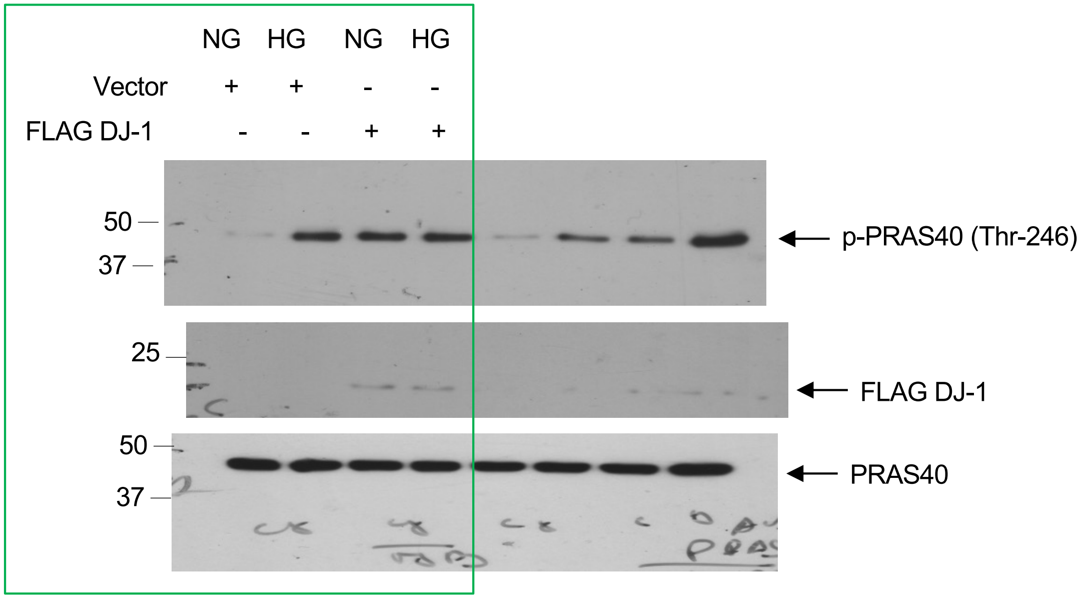

Fig. 3G

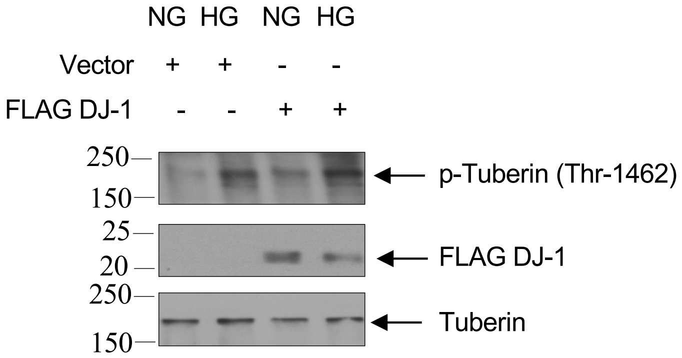

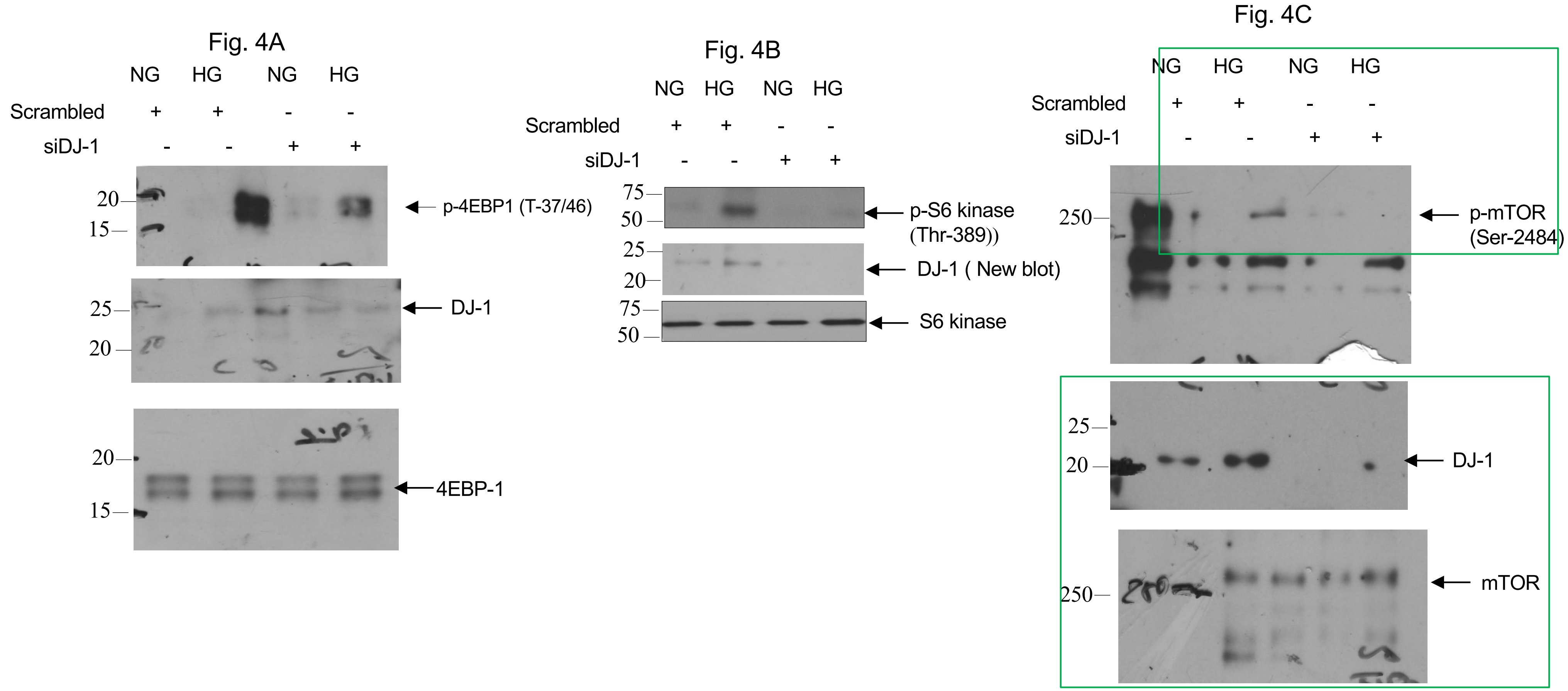

Fig. 4A
NG HG NG HG
Scrambled + + - -
siDJ-1 - - + +
20
15
p-4EBP1 (T-37/46)
25
20
DJ-1
20
15
4EBP-1
Fig. 4B
NG HG NG HG
Scrambled + + - -
siDJ-1 - - + +
75
50
p-S6 kinase (Thr-389))
25
20
DJ-1 (New blot)
75
50
S6 kinase
Fig. 4C
NG HG NG HG
Scrambled + + - -
siDJ-1 - - + +
250
p-mTOR (Ser-2484)
25
20
DJ-1
250
mTOR

Fig. 4D

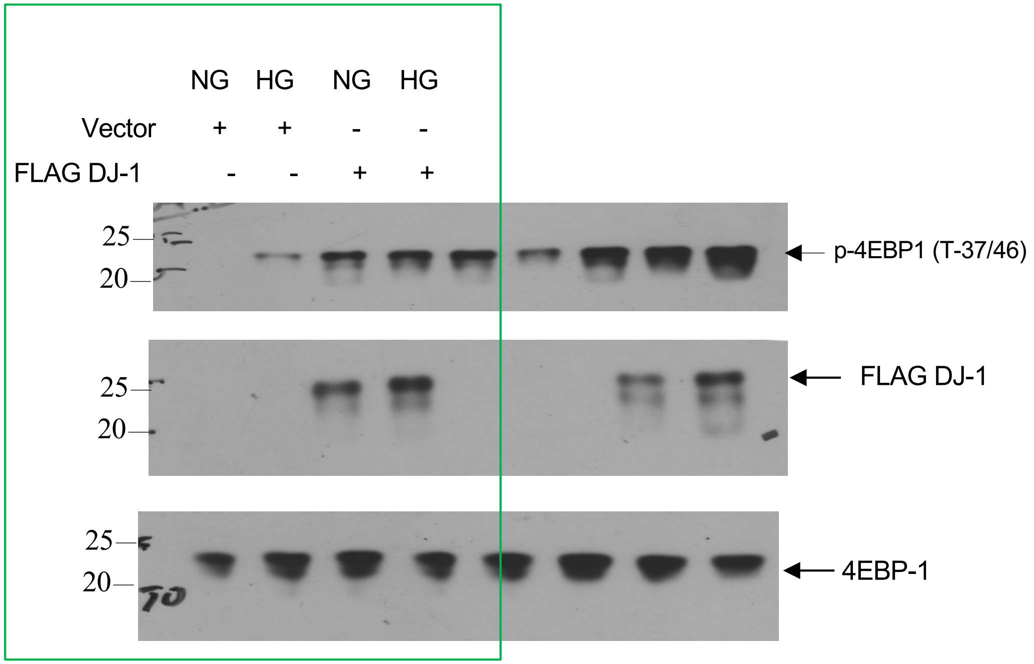

Fig. 4E

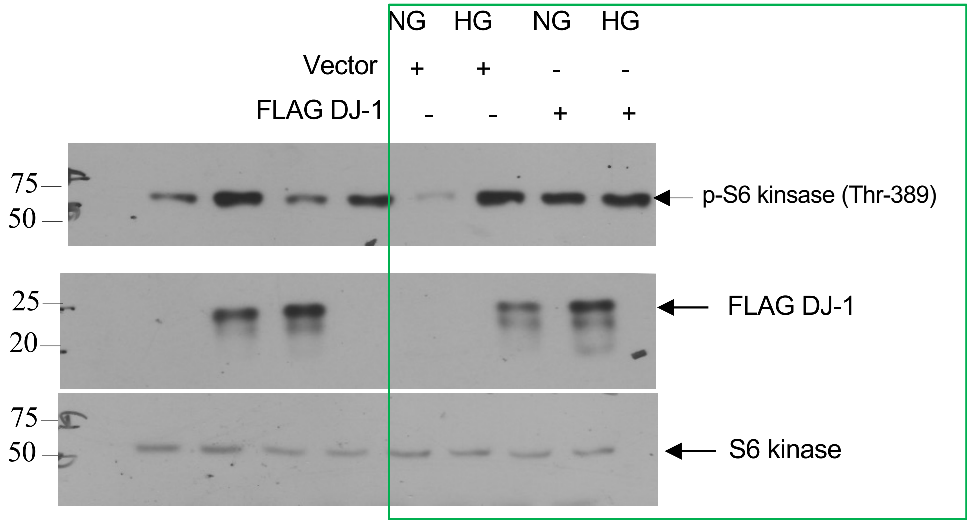

Fig. 4F

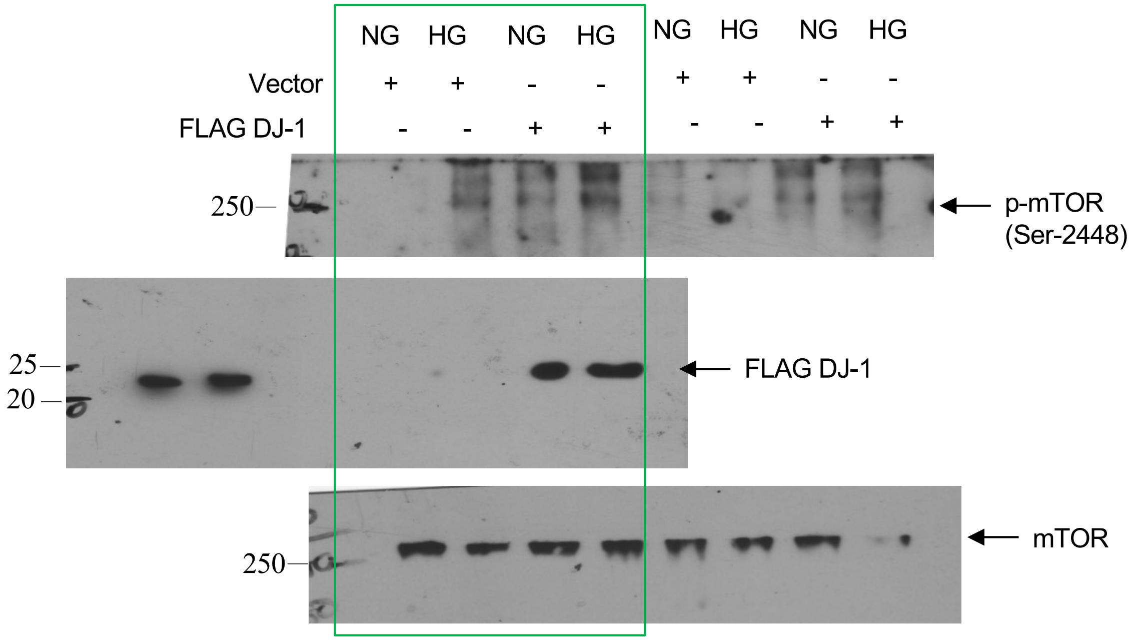

Fig. 5. Raw values and mean ± SD for Fig. 5A, 5B, 5C, 5D

A

79.53724	285.611	151.8438	209.6891
101.2292	271.1497	126.5365	187.9971
119.3059	253.073	133.7672	169.9205

Mean	100	269.9	137.4	189.2
SD	19.91	16.3	13.04	19.91

B

103.7245	185.1084	103.7245	108.5118
100.533	164.3634	102.1287	116.4906
100.533	180.3211	102.1287	111.7033
98.93723	199.4702	97.34146	108.5118
94.14994	180.3211	95.7457	111.7033
102.1287	173.938	95.7457	116.4906

Mean	100	180.6	99.47	112.2
SD	3.296	11.7	3.592	3.592

C

90.75848	180.0764	159.9078	205.287
118.4902	198.8043	154.8657	222.2142
90.75848	205.6472	158.4672	182.9576

Mean	100	194.8	157.7	203.5
SD	16.01	13.24	2.597	19.69

D

77.09924	175.5725	145.8015	192.3664
111.4504	177.8626	150.3817	163.3588
80.15267	188.5496	111.4504	170.9924
131.2977	192.3664	138.1679	198.4733

Mean	100	183.6	136.5	181.3
SD	26.01	8.139	17.41	16.79

Fig. 6A

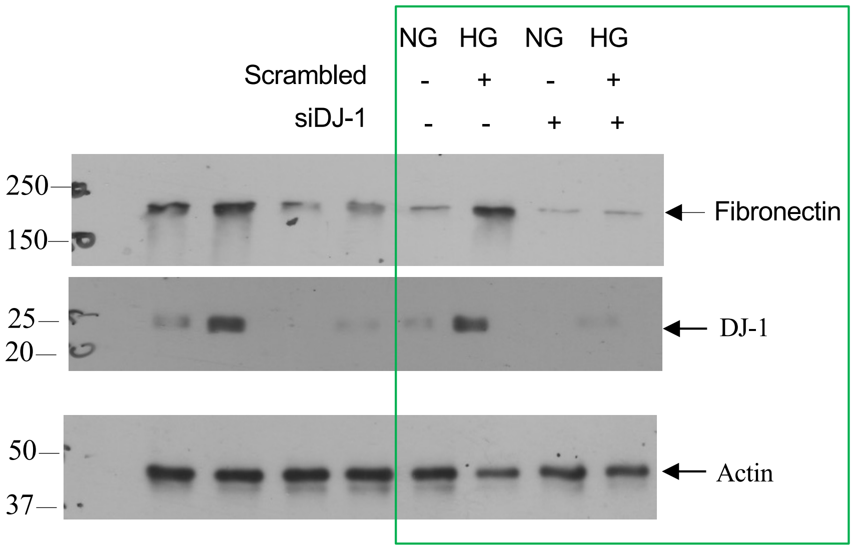

Fig. 6B

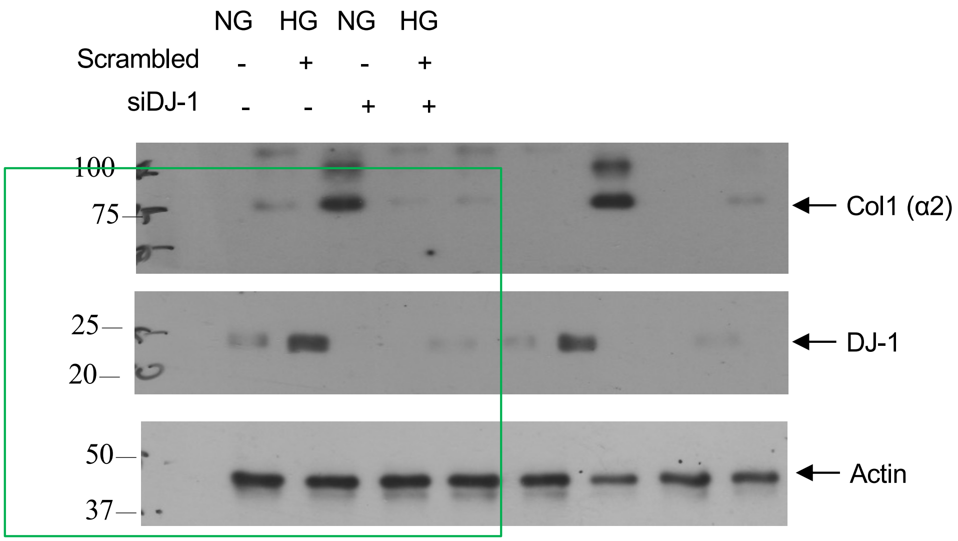

Fig. 6C

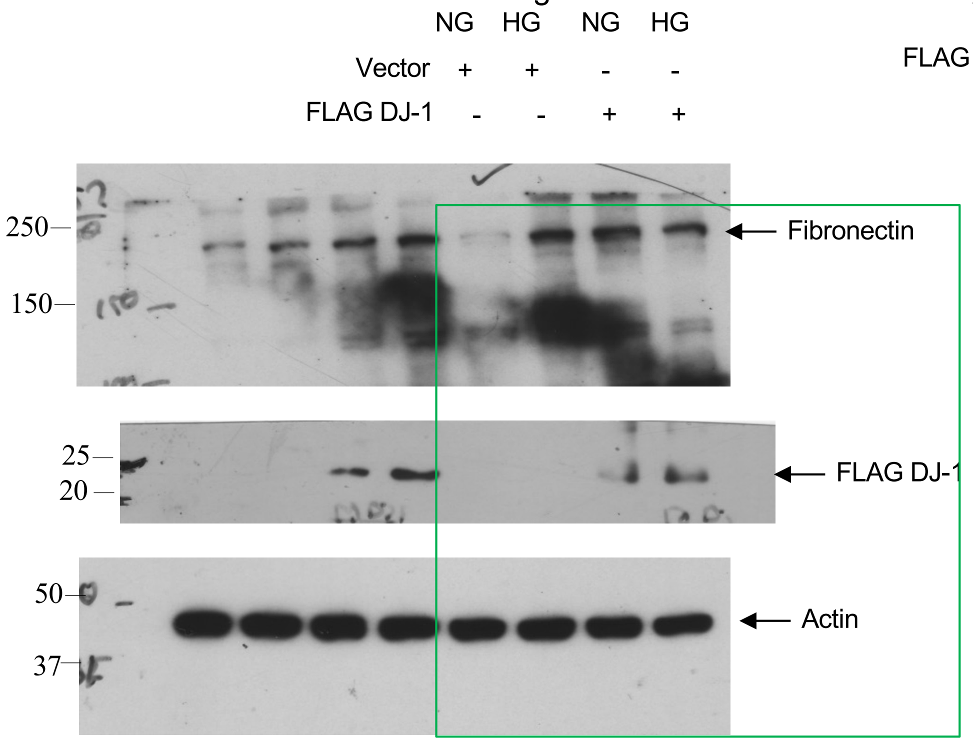

Fig. 6D

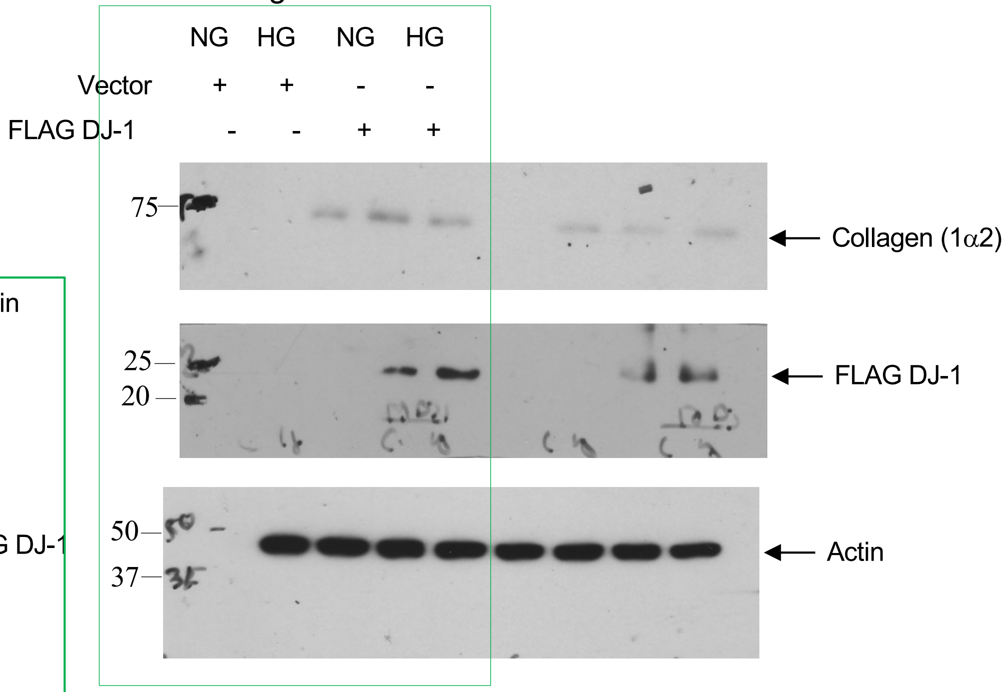

Fig. 7A Mean with SD and raw values

Mean	100	309.1	73.69	115.8
SD	17.31	49.94	9.558	45.61

81.82601	366.0637	68.90612	140.6833
116.2791	288.5444	67.47057	143.5544
101.9236	272.7534	84.6971	63.16394

Fig. 7B Mean with SD and raw values

Mean	100	153.7	93.18	108.6
SD	3.067	7.441	4.457	6.27

103.26	144.21	99.70	101.48
101.48	158.45	96.14	101.48
96.14	160.23	87.24	110.38
101.48	156.67	94.36	112.16
96.14	158.45	90.80	117.50
101.48	144.21	90.80	108.60

Fig. 7C

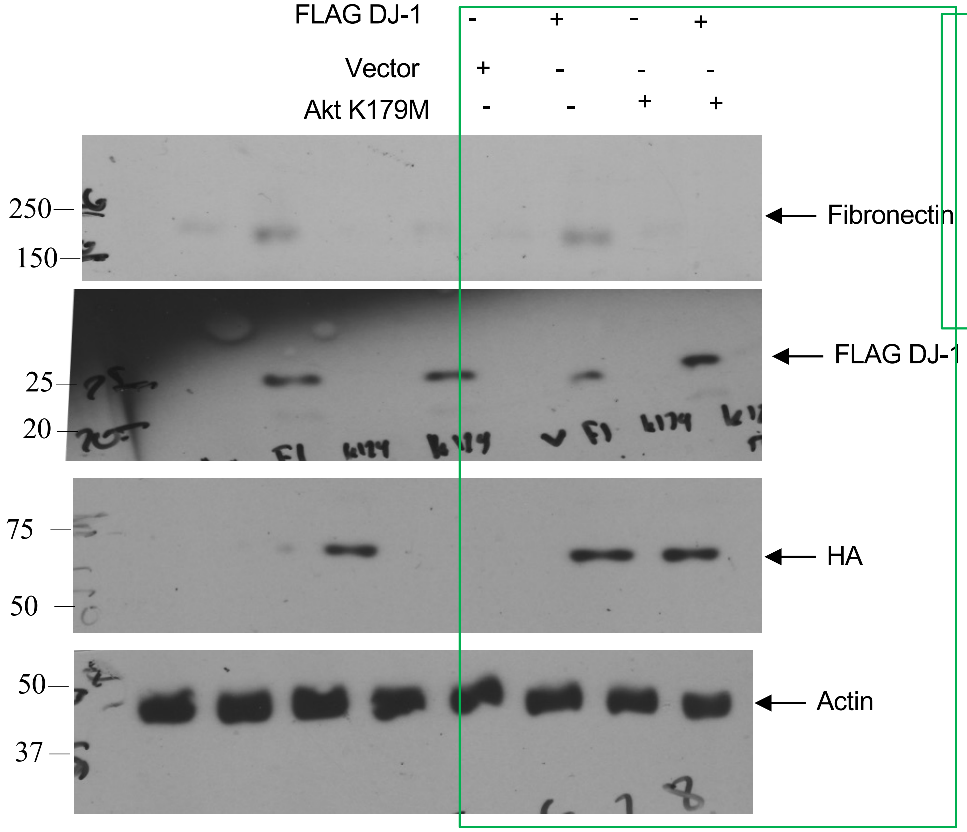

Fig. 7D

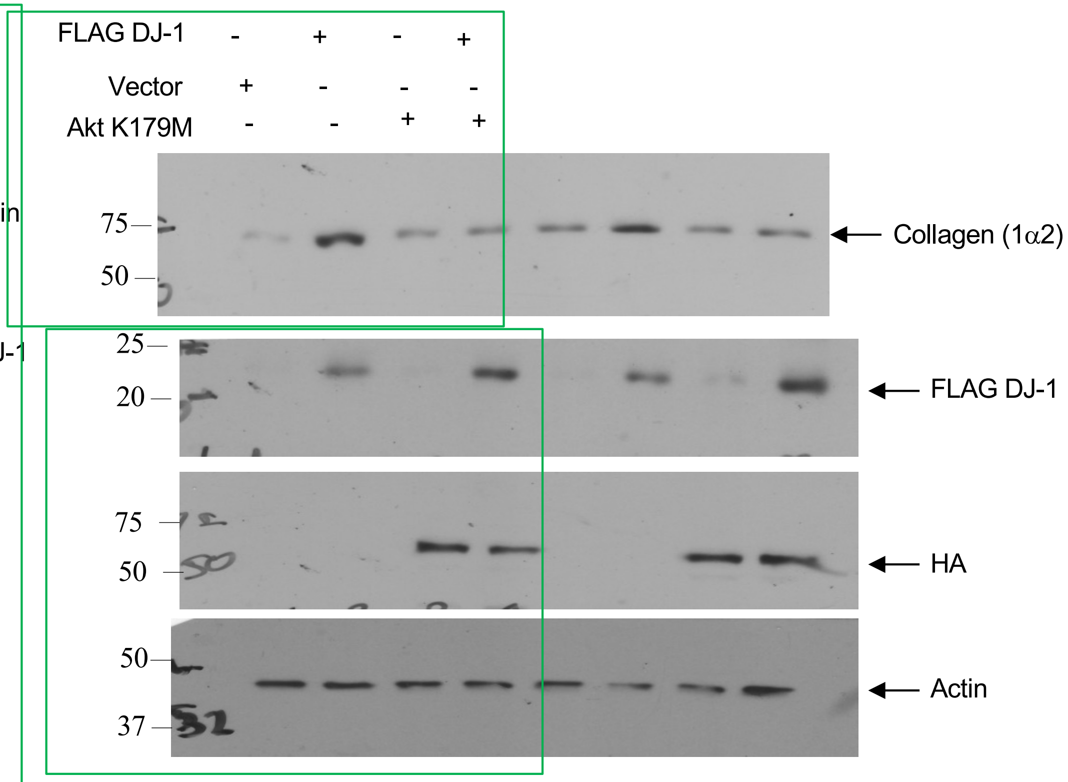

S7C

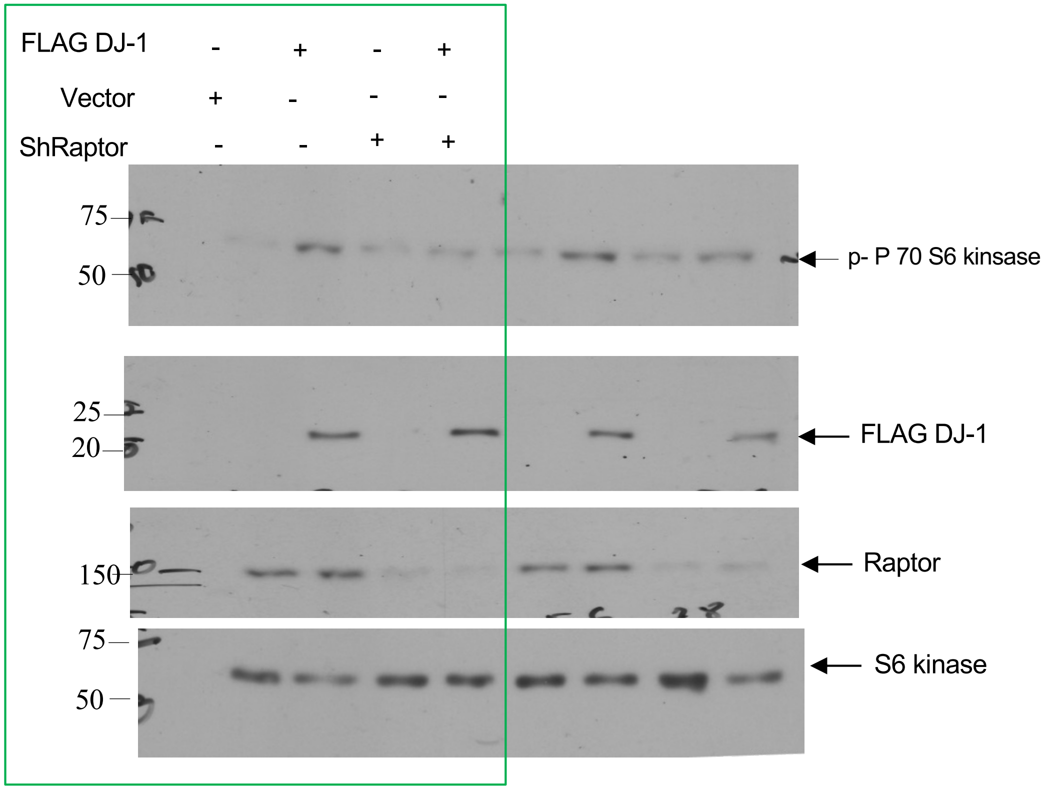

Fig. S7C Mean with SD and raw values

Mean	100.	228	94.44	112.3
Std. Deviation	38.34	34.28	40.07	37.87

91	216	78.66	100.66
142.33	266.66	140	154.66
67.33	201.33	64.66	81.66

Fig. 7E Mean with SD and raw values

Mean	100	245.8	71.92	145
SD	22.56	51.36	14.88	31.61

78.46	281.54	55.38	173.07
98.07	186.92	76.15	110.77
123.46	268.84	84.23	151.16

Fig. 7F Mean with SD and raw values

Mean	100	165.2	90.07	102.8
SD	8.29	6.94	7.07	9.09

108.51	172.34	85.10	95.74
102.12	168.08	97.87	114.89
110.63	159.57	91.48	89.36
91.48	163.82	95.74	104.25
93.617	155.31	78.72	104.25
93.61	172.34	91.48	108.51

Fig. S7E Mean with SD and raw values

Mean	100	348	112.2	100.6
SD	21.86	36.59	57.42	32.52

103.46	378.07	90.38	129.23
79.61	371.15	104.23	96.15
129.23	345.38	193.84	120.38
87.69	297.30	60.38	56.53

Fig. S7F Mean with SD and raw values

Mean	100	500.6	100.6	139.8
SD	15.61	68.3	20.61	58.61

92.07	599.39	81.70	78.65
89.63	454.87	129.26	189.02
95.12	454.87	90.85	190.85
123.17	493.29	100.60	100.60

Fig. 7G

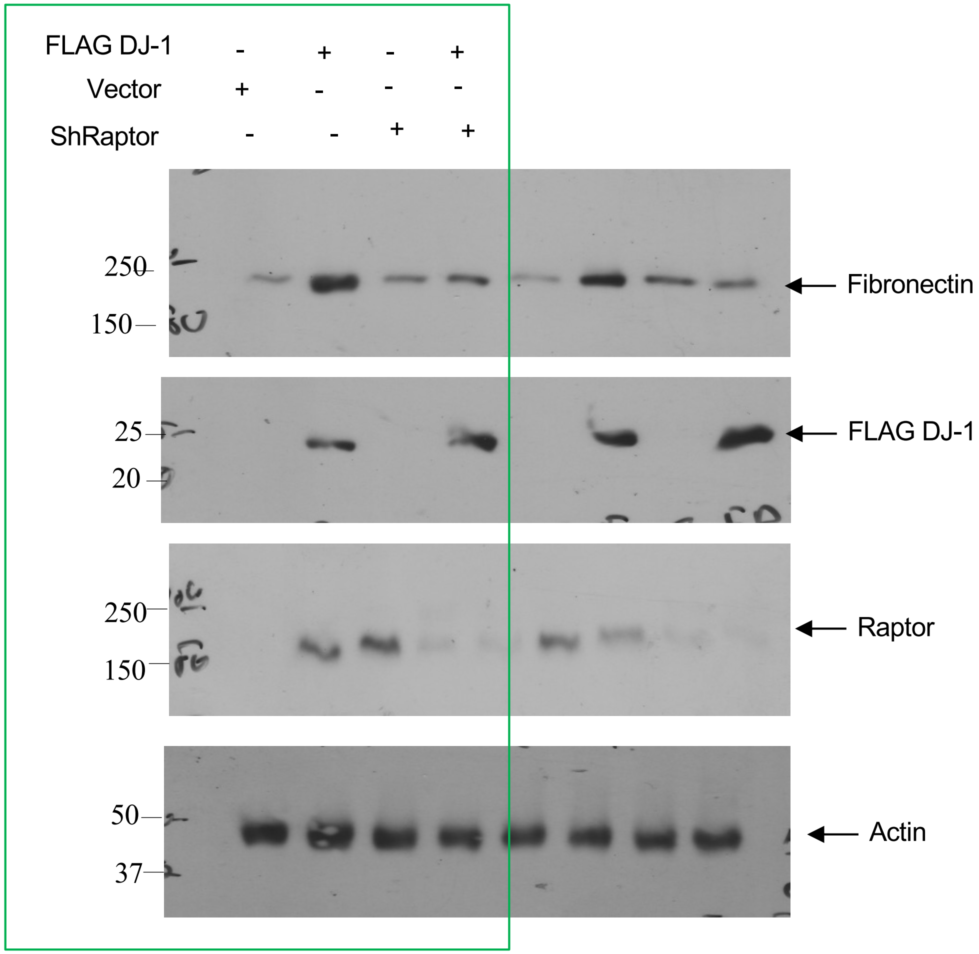

Fig. 7H

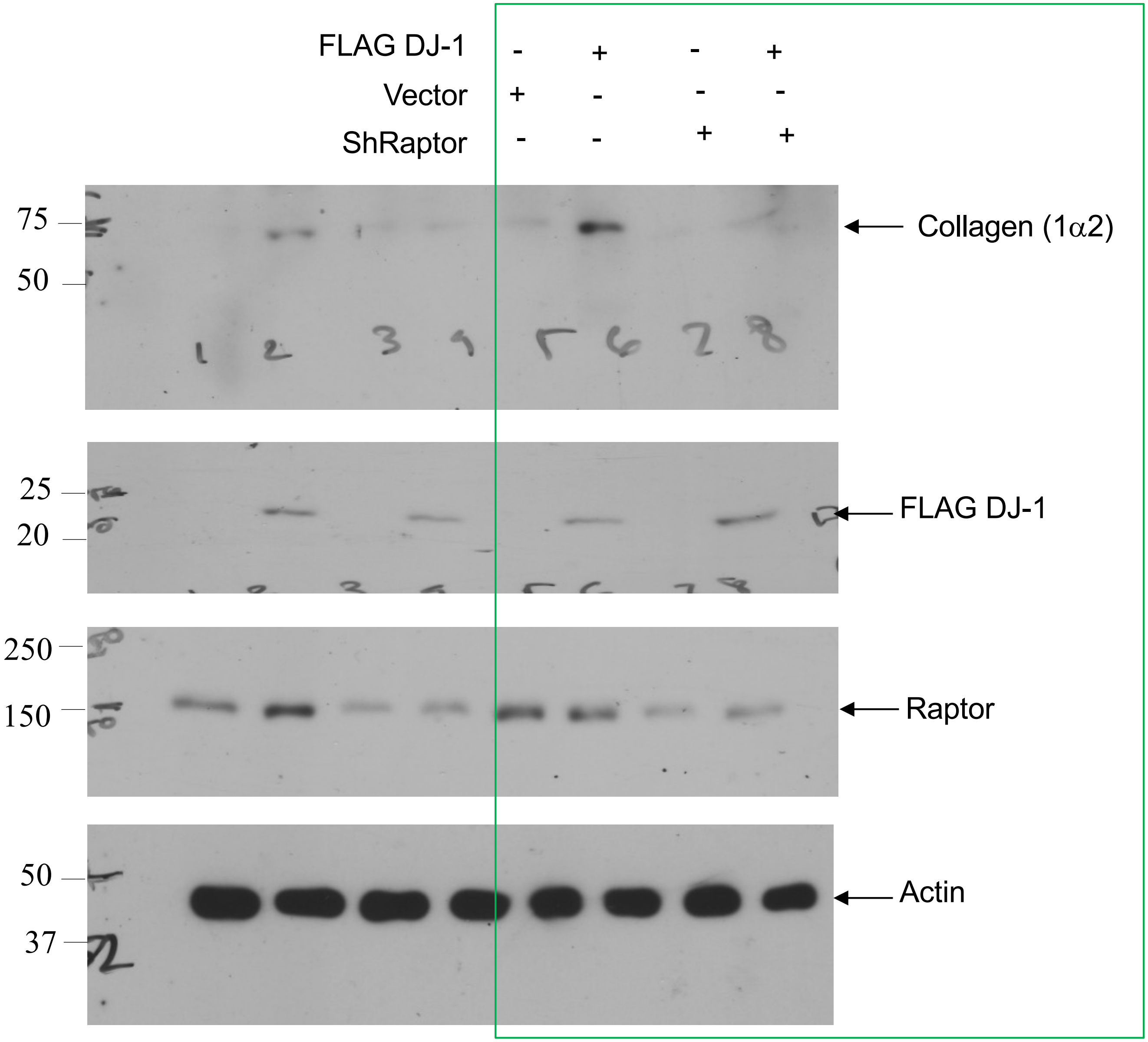

FLAG DJ-1 - + - +
Vector + - - -
ShRaptor - - + +
75
50
Collagen (1α2)
25
20
FLAG DJ-1
250
150
Raptor
50
37
Actin

Fig. 8A

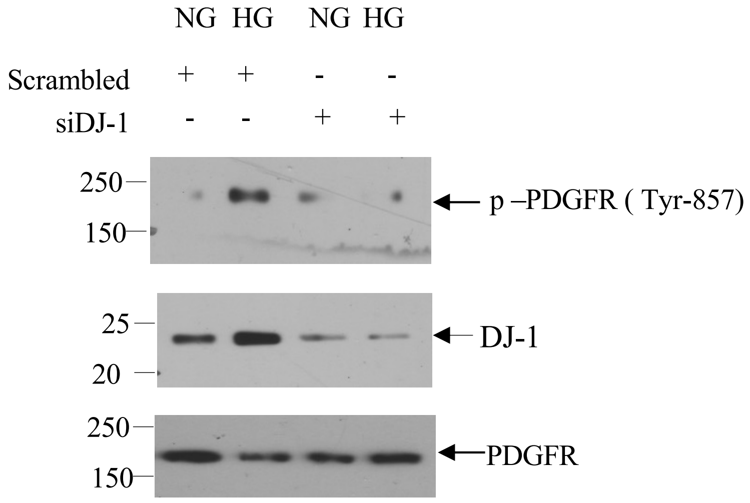

Fig. 8B

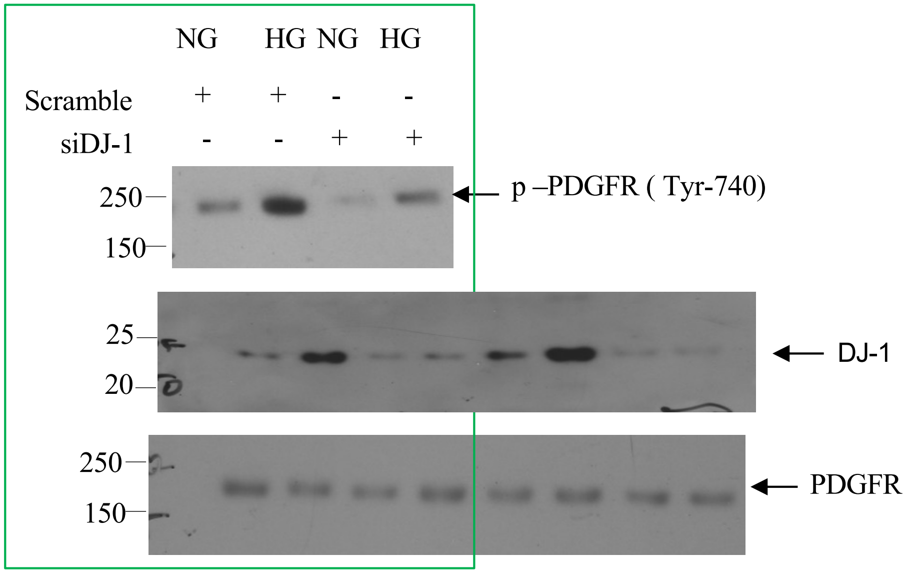

Fig. 8C

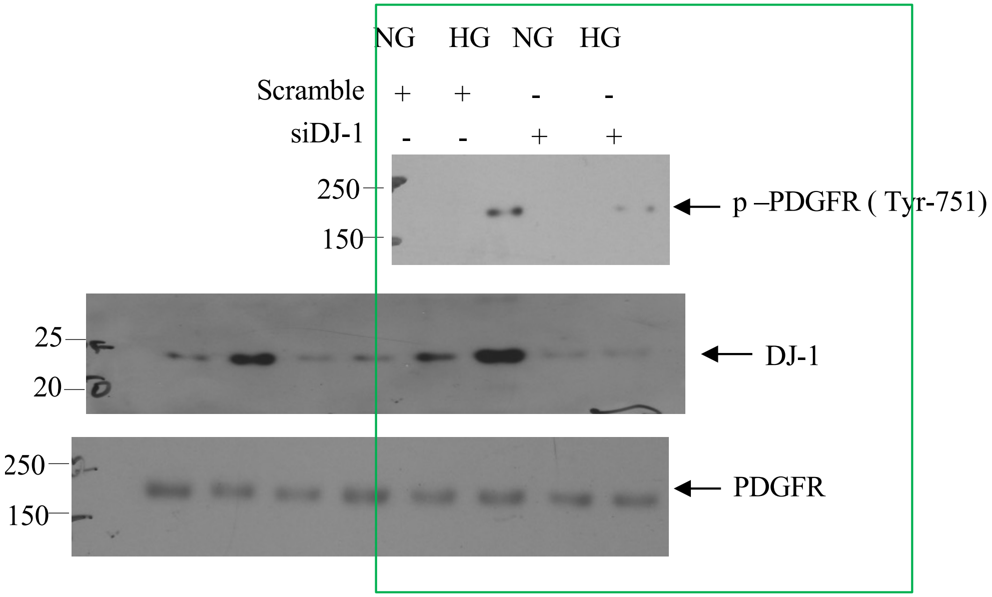

Fig 8D.

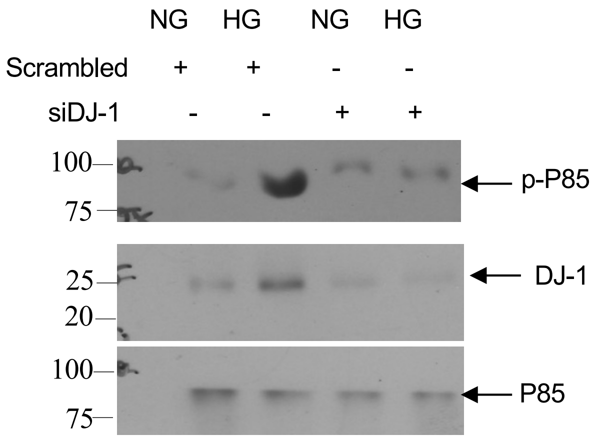

Fig 8E

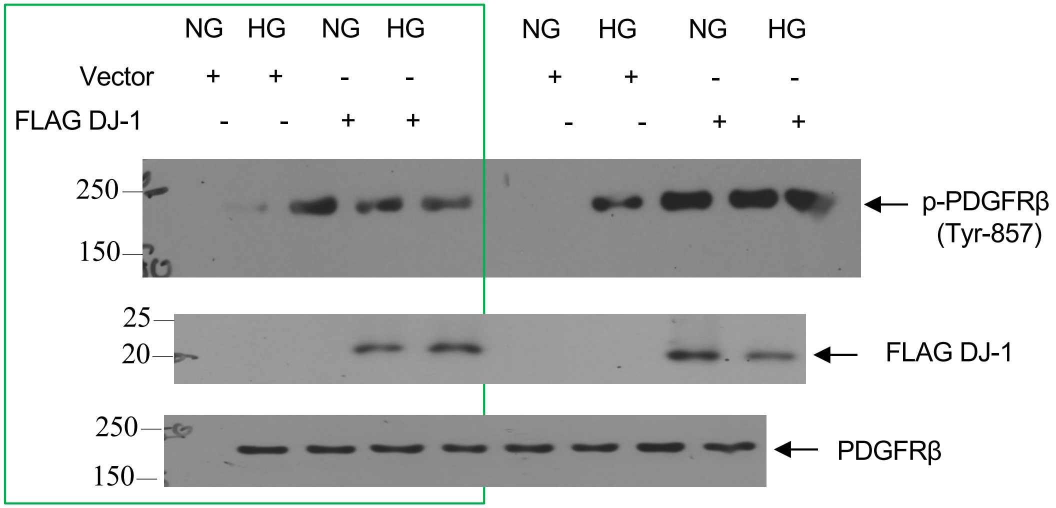
NG HG NG HG NG HG NG HG
Vector + + - - + + - -
FLAG DJ-1 - - + + - - + +
250
150
p-PDGFRβ (Tyr-857)
25
20
FLAG DJ-1
250
150
PDGFRβ

Fig 8F

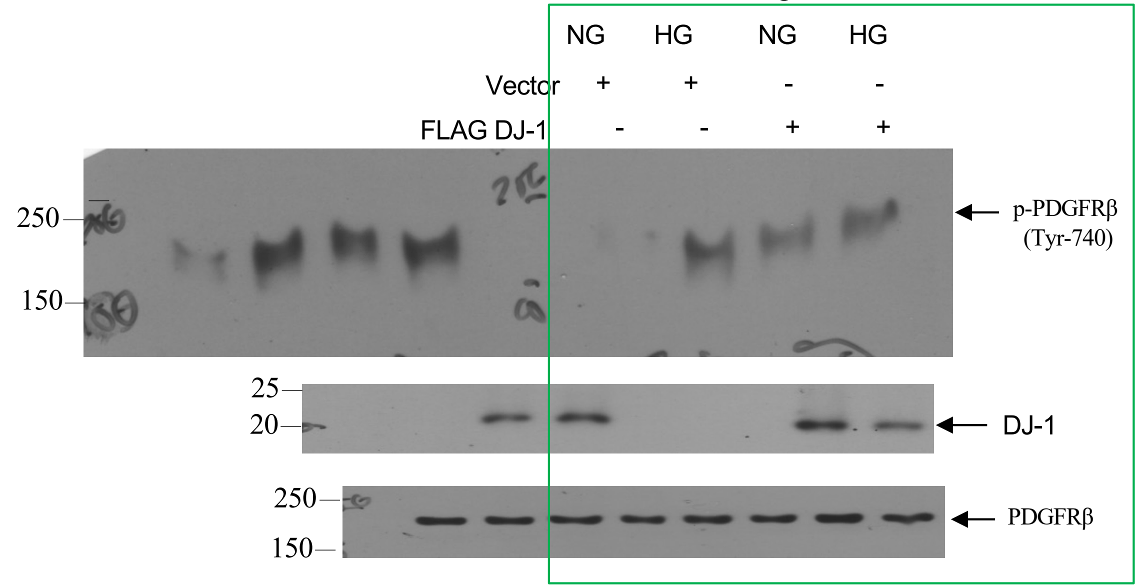
NG HG NG HG
Vector + + - -
FLAG DJ-1 - - + +
250
150
p-PDGFRβ (Tyr-740)
25
20
DJ-1
250
150
PDGFRβ

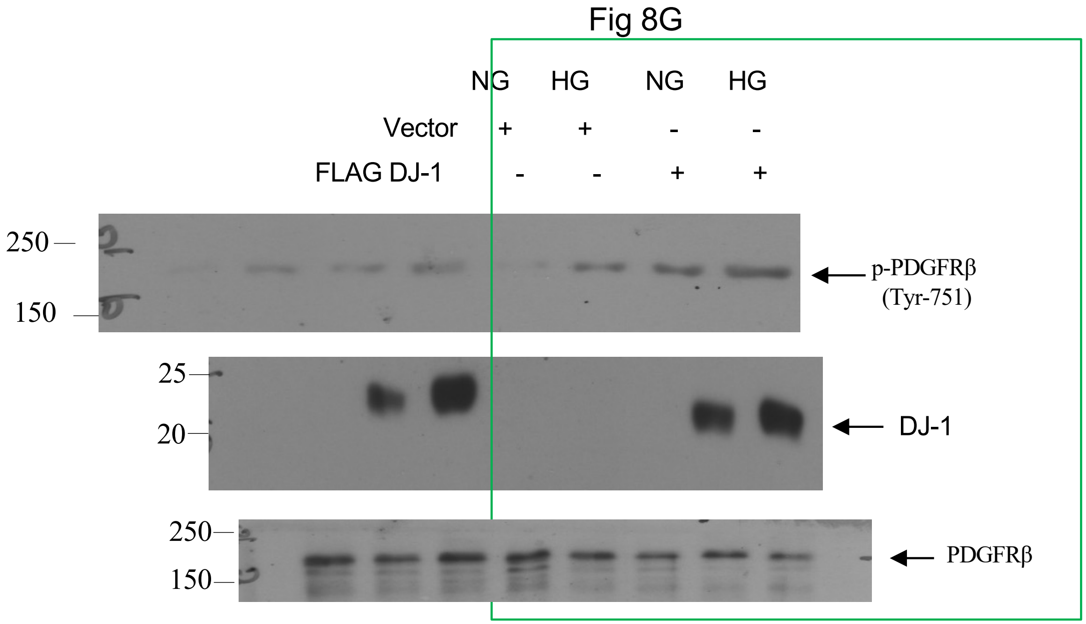
Fig 8G
NG HG NG HG
Vector + + - -
FLAG DJ-1 - - + +
250
150
p-PDGFRβ (Tyr-751)
25
20
DJ-1
250
150
PDGFRβ

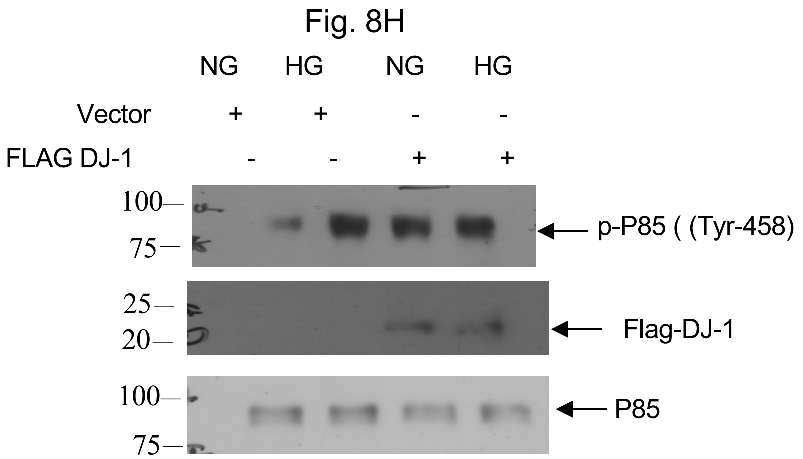
Fig. 8H
NG HG NG HG
Vector + + - -
FLAG DJ-1 - - + +
100
75
p-P85 ((Tyr-458)
25
20
Flag-DJ-1
100
75
P85

Fig. 9A

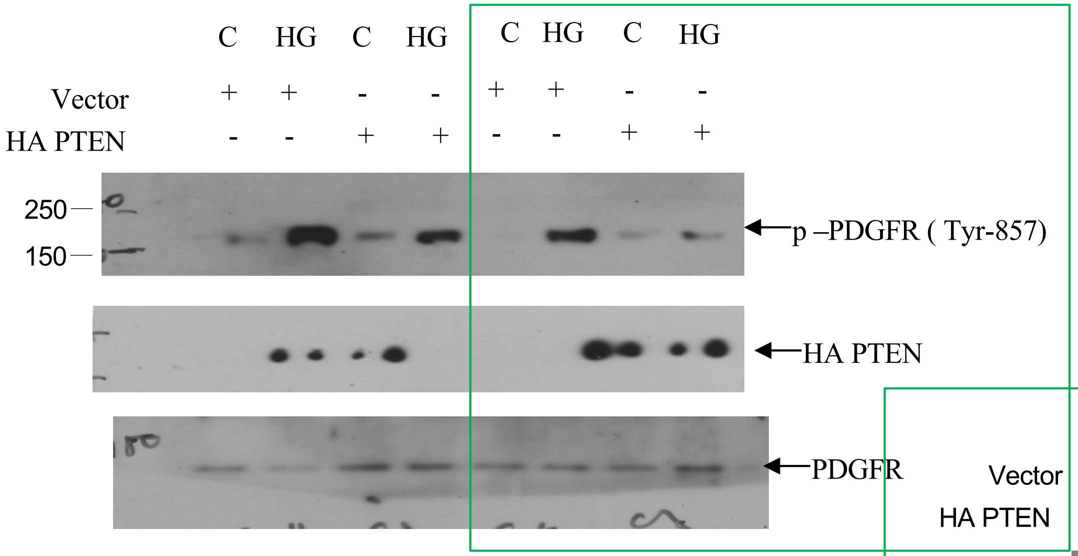

Fig. 9C

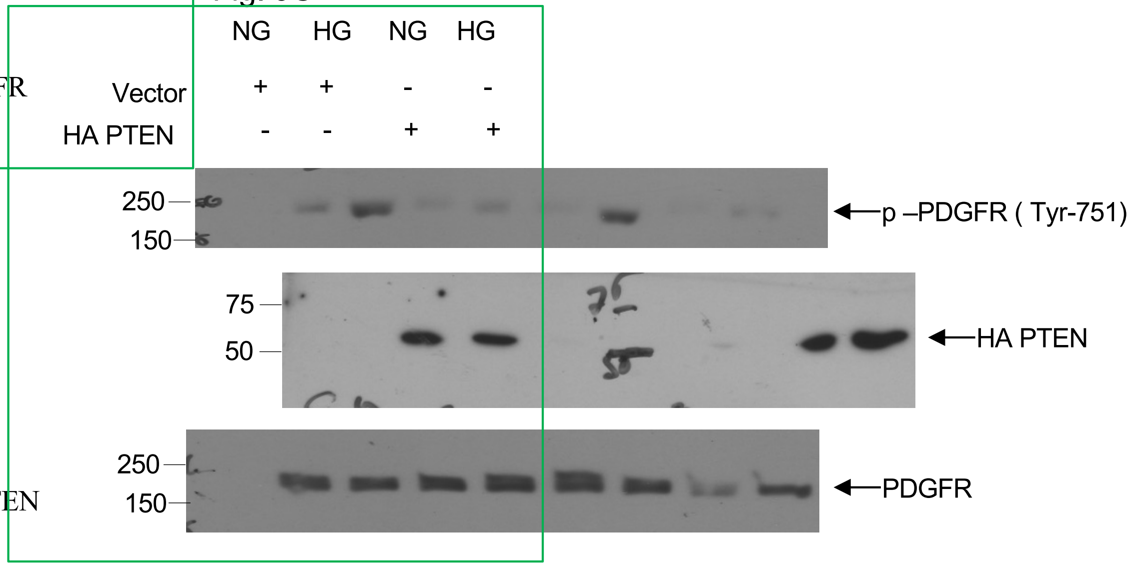

Fig. 9B

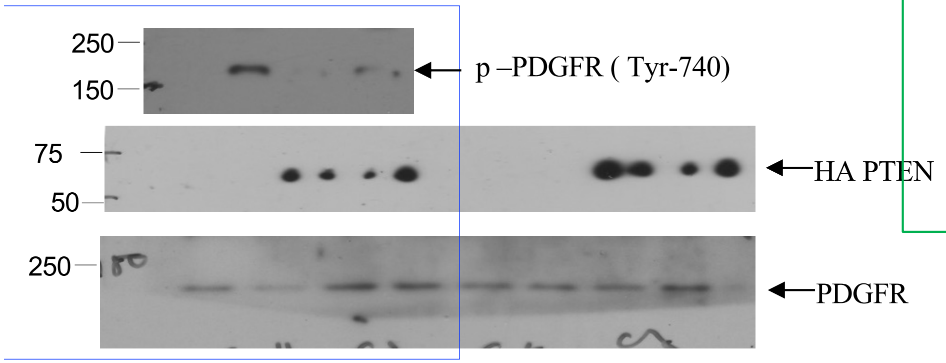

Fig 9D

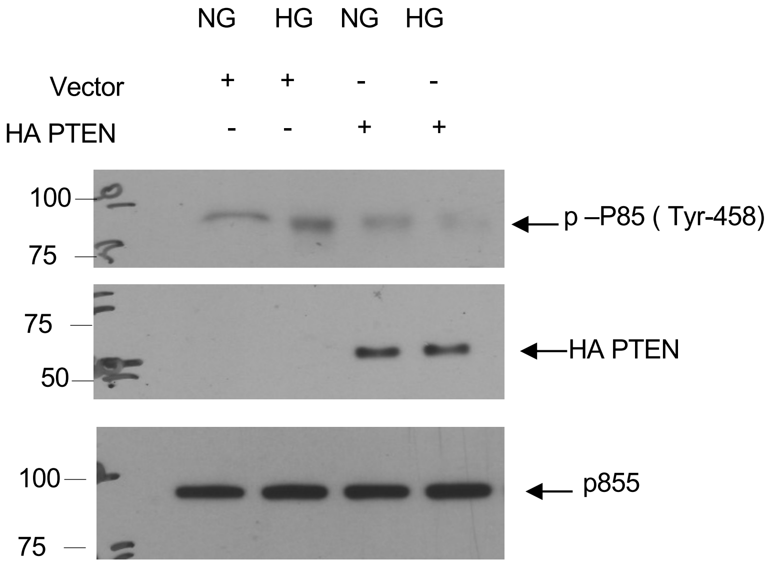
NG HG NG HG
Vector + + - -
HA PTEN - - + +
100
75
p –P85 (Tyr-458)
75
50
HA PTEN
100
75
p855

Fig 9E.

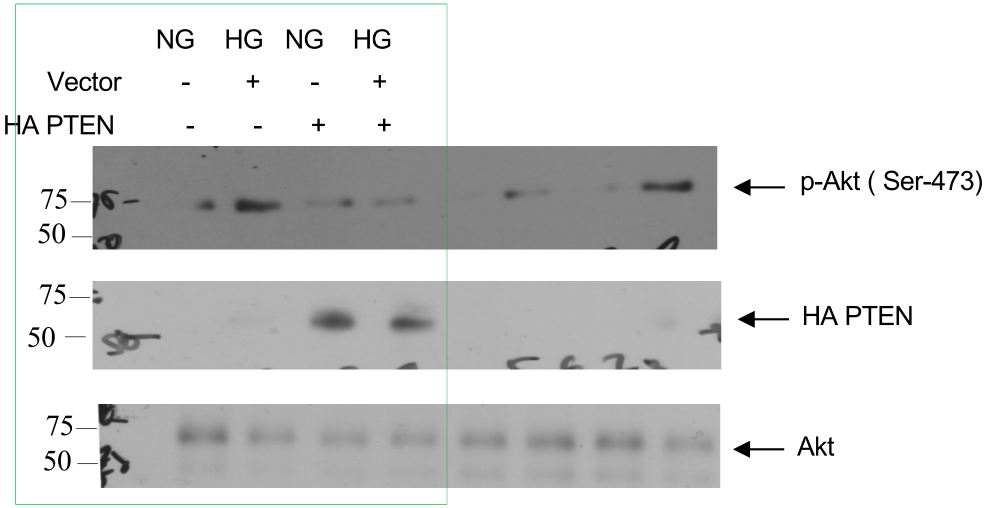
NG HG NG HG
Vector - + - +
HA PTEN - - + +
75
50
p-Akt (Ser-473)
75
50
HA PTEN
75
50
Akt

Raw data for S1A

Mean	100	320.6	420	443.4	421.2
SD	16.26	48.06	29.11	34.53	6.08

0 hr	2 hr	4 hr	6 hr	8 hr
75.78	228.15	477.36	512.46	421.20
93.36	389.61	400.14	407.16	410.67
130.92	343.98	382.59	417.67	431.73

Raw data for S1B

Mean	100	270.4	290.5
SD	16.91	17.74	8.83

0 hr	24 hr	48 hr
77.09	268.15	282.12
45.53	209.49	273.74
120.11	262.56	276.53
125.69	307.26	298.88
134.07	304.46	321.22

Raw data for S1C

Mean	100	472.4	572.6	529.7	546.0
SD	7.08	62.96	108.1	1o3.1	98.73

0 hr	2 hr	4 hr	6 hr	8 hr
104.29	539.87	568.28	525.14	576.68
92.02	460.12	687.11	619.63	625.76
104.29	417.17	472.39	417.17	435.58

Raw data for S1D

100	327.1	360.6
22.42	41.39	46.02

81.456200	279.863500	354.948800
127.417500	364.050000	409.556300
109.215000	359.499400	377.701900
81.911260	304.891900	300.341300

Raw data for S2A

Mean	100	381.6
SD	22.14	78.24
	105.07	463.76
	76.08	307.97
	119.56	373.18

Raw data for S2B

Mean	100	436.2
SD	22.25	63.56
	144.53	472.65
	82.03	523.43
	74.21	312.5

Raw data for S2C

Mean	100	241.1
SD	43.36	29.73
	134.14	263.41
	51.21	207.31
	114.63	252.43

Raw data for S2D

Mean	100	281.9
SD	22.99	40.54
	74.28	245.71
	118.57	325.71
	107.14	274.28

Raw data for S2E

Mean	100	274.3
SD	20.6	17.36
	77.14	265.71
	105.71	262.85
	117.14	294.28

Raw data for S2F

Mean	100	318.8
SD	17.21	12.5
	84.37	306.25
	118.75	318.75
	100.0	331.25

S3A

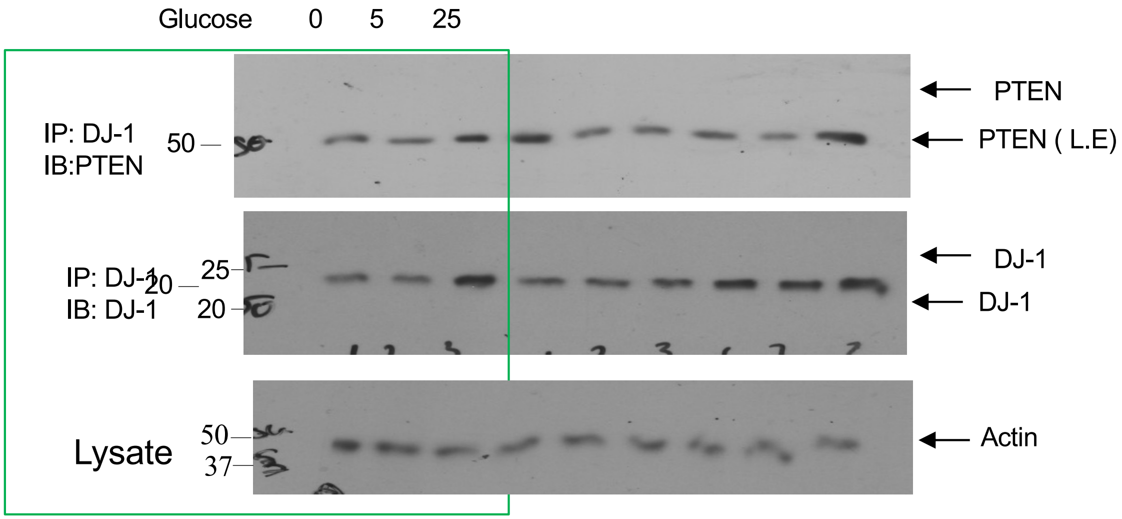

S3B raw data

Mean	100	94.77	181.1
Std. Deviation	11.31	4.57	21.04

71.92	70.19	223.82
111.62	102.99	323.93
116.51	114.49	319.90

S3C

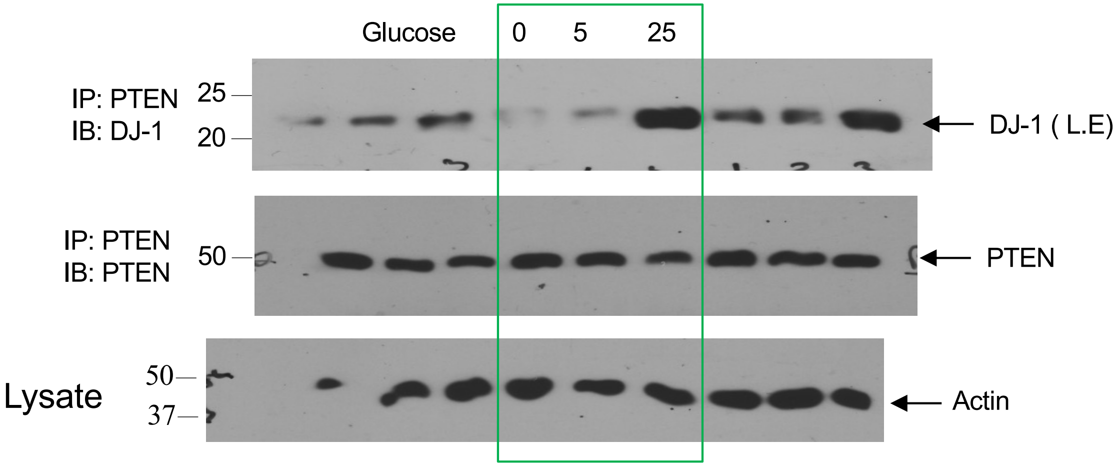

S3D raw data

Mean	100	105.8	273.6
Std. Deviation	29.37	29.85	43.49

121.28	126.76	223.39
66.51	71.59	299.29
112.28	118.93	298.12

Raw data for S4A

Mean	100	314.7	96.53	113.2
SD	31.2	98.21	28.53	50.54
	135..7	367.03	128.75	170.56
	86.4	201.44	86.65	93.87
	77.93	375.74	74.2	75.19

Raw data for S4B

Mean	100	286.2	82.41	137.2
SD	26.35	35.24	24.41	28.97
	70.35	288.59	55.04	128.01
	120.52	249.83	90.22	114.0
	109.44	320.19	101.95	169.7

Raw data for S4C

Mean	100	231.5	81.35	91.22
SD	5.99	6.63	17.99	13.18
	93.25	237.97	62.24	99.32
	102.02	224.71	97.972	100.49
	104.71	231.68	83.82	77.07

Raw data for S4D

Mean	100	350.3	79.48	88.12
SD	40.5	101.6	14.45	21.77
	146.75	46759	95.37	110.18
	75.92	288.42	67.12	87.50
	77.31	294.90	75.92	66.66

Raw data for S4E

Mean	100	307.2	314	290.66
SD	35.29	88.62	94.89	63.62
	140.06	392.05	402.64	355.62
	86.42	314.23	325.49	287.74
	73.50	215.23	213.90	228.47

Raw data for S4F

Mean	102	321.7	318.7	325.4
SD	27.7	38.94	43.6	39.54
	79.15	303.25	270.03	288.92
	131.59	295.43	331.59	319.86
	89.90	366.44	354.39	367.42

Raw data for S4G

Mean	100	392.2	363.4	415
SD	54.8	139.1	128.4	126.4
	161.67	529.92	501.82	558.75
	56.93	394.89	340.16	304.96
	81.38	251.82	248.17	321.16

Raw data for S4H

Mean	100	401.1	409	405.4
SD	20.7	91.59	81.22	92.86
	92.13	491.57	488.76	494.94
	123.03	308.42	326.40	309.55
	85.39	403.37	411.79	411.79

Raw data for S5A

Mean	100	417	83.33	138
SD	27.21	33.72	17.83	90.15

88.42	454.21	77.68	238.42
131.40	409.24	103.30	111.57
80.99	388.21	69.00	64.04

Raw data for S5B

Mean	100	425	82.12	119.1
SD	24.45	65.36	1.591	47.44

83.85	350.00	83.85	85.93
128.12	455.20	80.72	97.91
88.02	469.79	81.77	173.43

Raw data for S5C

Mean	100	326.2	104.1	102.5
SD	70.22	103.9	62.31	61.77

180.84	435.38	172.07	171.10
64.93	228.57	90.58	51.29
54.22	314.61	49.67	85.06

Raw data for S5D

Mean	100	323	308.6	348.4
SD	21.27	100	88.54	89.21

99.78	250.00	243.42	288.15
121.49	282.01	273.02	306.14
78.94	437.06	409.42	450.87

Raw data for S5E

Mean	100	420	362.5	411.4
SD	42.45	71.85	106.9	93.61

78.19	493.31	306.39	447.09
149.12	416.86	485.75	481.97
73.25	349.70	295.34	305.23

Raw data for S5F

Mean	100	380	376.9	435.8
SD	34.22	121.3	139.9	119.7

139.68	519.84	535.71	568.25
78.17	317.46	272.22	335.31
82.93	302.77	322.61	403.96

Raw data for Fig. 5A

Mean	100	269.9	137.4	189.2
SD	19.91	16.3	13.04	19.91

79.53724	285.611	151.8438	209.6891
101.2292	271.1497	126.5365	187.9971
119.3059	253.073	133.7672	169.9205

Raw data for Fig. 5B

Mean	100	269.9	137.4	189.2
SD	19.91	16.3	13.04	19.91

79.53724	285.611	151.8438	209.6891
101.2292	271.1497	126.5365	187.9971
119.3059	253.073	133.7672	169.9205

Raw data for Fig. 5C

Mean	100	194.8	157.7	203.5
SD	16.01	13.24	2.597	19.69

90.75848	180.0764	159.9078	205.287
118.4902	198.8043	154.8657	222.2142
90.75848	205.6472	158.4672	182.9576

Raw data for Fig. 5D

Mean	100	183.6	136.5	181.3
SD	26.01	8.139	17.41	16.79

77.09924	175.5725	145.8015	192.3664
111.4504	177.8626	150.3817	163.3588
80.15267	188.5496	111.4504	170.9924
131.2977	192.3664	138.1679	198.4733

Raw data for S6A

Mean	100	340.9	101.2	128.5
SD	14	94.3	36.08	49.77

86.24	314.42	111.68	128.65
114.23	262.68	61.07	78.60
99.53	445.62	130.91	178.13

Raw data for S6B

Mean	100	316.8	109.1	133.2
SD	16.74	76.08	33.52	51.85

119.27	267.18	100.52	107.03
89.06	278.90	80.72	99.73
91.66	404.42	146.09	192.96

Raw data for S6C

Mean	100	320.4	298.2	324.3
SD	58.98	64.81	38.45	70.41

37.61	250.63	256.40	247.40
107.54	331.87	306.02	385.64
154.85	378.72	332.10	339.71

Raw data for S6D

Mean	100	278.6	293	295.8
SD	38.47	67.08	80.18	68.12

59.76	205.62	202.07	229.58
104.14	292.60	323.37	292.01
136.39	337.57	353.55	365.68

Raw data for Fig. 7A

Mean	100	309.1	73.69	115.8
SD	17.31	49.94	9.558	45.61

81.82601	366.0637	68.90612	140.6833
116.2791	288.5444	67.47057	143.5544
101.9236	272.7534	84.6971	63.16394

Raw data for Fig. 7B

Mean	100	153.7	93.18	108.6
SD	3.067	7.441	4.457	6.27

103.26	144.21	99.70	101.48
101.48	158.45	96.14	101.48
96.14	160.23	87.24	110.38
101.48	156.67	94.36	112.16
96.14	158.45	90.80	117.50
101.48	144.21	90.80	108.60

Raw data for S7A

Mean	100	294.7	98.93	120.2
SD	32.42	63.98	32.56	21.55

72.93	224.63	67.50	88.84
72.16	280.89	76.81	124.92
119.10	293.69	114.06	129.19
135.79	379.43	137.34	137.73

Raw data for S7B

Mean	100	291.7	93.02	120.7
SD	13.76	27.98	4.85	18.16

79.54	289.48	97.51	105.54
105.16	292.54	95.60	131.54
105.92	258.12	86.42	105.16
109.36	326.57	92.54	140.72

Raw data for S7D

Mean	100.	228	94.44	112.3
Std. Deviation	38.34	34.28	40.07	37.87

91	216	78.66667	100.6667
142.3333	266.6667	140	154.6667
67.33333	201.3333	64.66667	81.66667

Raw data for Fig. 7E

Mean	100	245.8	71.92	145
SD	22.56	51.36	14.88	31.61

78.46	281.54	55.38	173.07
98.07	186.92	76.15	110.77
123.46	268.84	84.23	151.16

Raw data for Fig. 7F

Mean	100	165.2	90.07	102.8
SD	8.295	6.949	7.078	9.094

108.51	172.34	85.10	95.74
102.12	168.08	97.87	114.89
110.63	159.57	91.48	89.36
91.48	163.82	95.74	104.25
93.61	155.31	78.72	104.25
93.61	172.34	91.48	108.51

Raw data for Fig. S7E

Mean	100	348	112.2	100.6
SD	21.86	36.59	57.42	32.52

103.46	378.07	90.38	129.23
79.61	371.15	104.23	96.15
129.23	345.38	193.84	120.38
87.69	297.30	60.38	56.53

Raw data for Fig. S7F

Mean	100	500.6	100.6	139.8
SD	15.61	68.3	20.61	58.61

92.07	599.39	81.70	78.65
89.63	454.87	129.26	189.02
95.12	454.87	90.85	190.85
123.17	493.29	100.60	100.60

SupplementaryFig. S8A

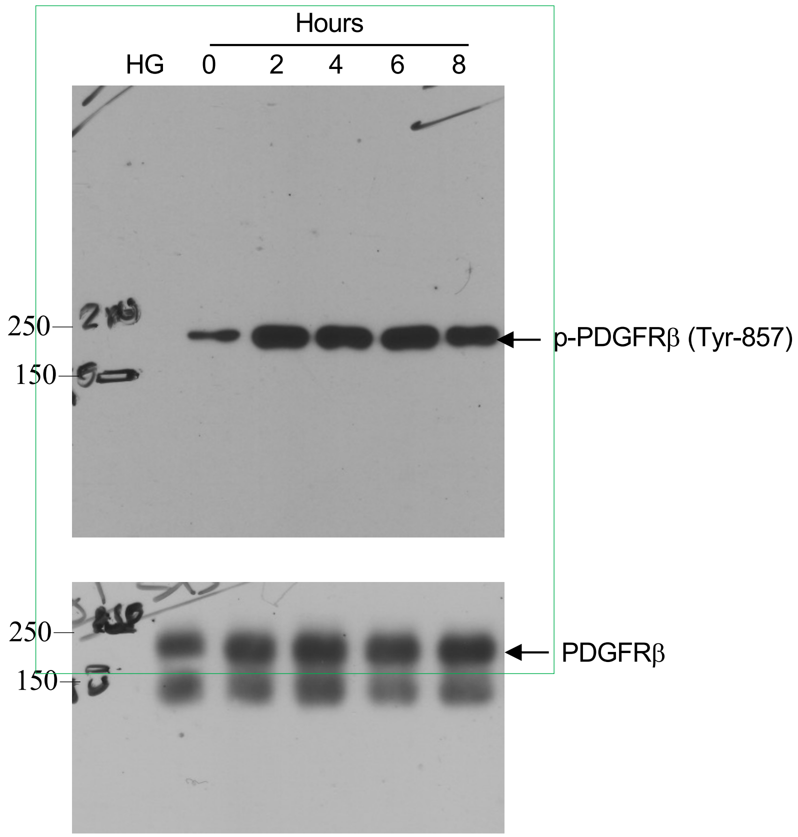

Raw values of S8B

Mean	100	230.5	230.6	260.7	263.1
SD	23.01	73.79	79.39	78.33	64.88

89.00	190.57	176.17	241.62	284.29
130.89	332.98	337.69	375.39	345.54
102.61	232.98	243.45	225.65	212.82
77.48	165.44	164.92	200.18	209.68

SupplementaryFig. S8C

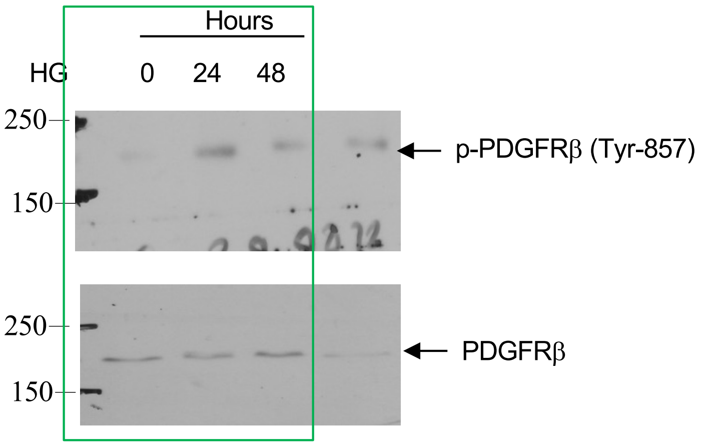

Raw values of S8D

Mean	100	219.3	217.4
SD	32.2	67.51	69.79

54.77	125.56	142.75
102.54	256.37	213.83
129.93	278.51	311.14
112.74	216.75	201.89

Raw values of S9A

Mean	100	374.6	111.2	154.8
SD	16.1	42.74	38.29	45.93

106.77	346.18	152.11	186.44
111.86	353.81	105.08	175.84
81.77	423.72	76.27	102.11

Raw values of S9B

Mean	100	340.8	112.5	121
SD	22.34	22.98	4.07	30.75

115.26	321.80	117.13	150.46
110.59	366.35	110.59	123.36
74.454	334.26	109.65	89.09

Raw values of S9C

Mean	100	295.2	98.66	123.3
SD	16.51	26.81	16.73	10.55

118.73	264.26	113.25	134.29
94.23	309.51	102.30	122.47
87.31	311.81	80.40	113.25

Raw values of S9D

Mean	100	318	83.42	121.8
SD	21.22	83.49	51.06	43.5

98.91	414.40	141.03	170.38
122.01	271.73	65.48	108.69
79.61	267.93	43.75	86.41

Raw values of S9E

Mean	100	327	311.1	327.7
SD	35.91	25.88	24.24	11.77

60.84	341.42	326.53	335.27
107.76	297.08	283.17	311.97
131.39	342.39	323.62	320.71

Raw values of S9F

Mean	100	315.6	288.4	298.3
SD	19.6	42.23	60.13	60.68

81.69	271.86	220.67	234.23
97.62	323.33	308.81	305.76
120.67	355.25	335.59	354.91

Raw values of S9G

Mean	100	274.3	266.8	280.4
SD	16.76	21.94	24.87	25.54

115.18	296.96	288.10	303.79
82.02	253.16	239.49	253.16
102.78	272.65	272.91	284.30

Raw values of S9H

Mean	100	331.2	314.9	326.2
SD	13.44	16.09	9.50	31.08

107.87	320.09	325.83	348.77
84.49	323.84	309.94	290.75
107.65	349.65	308.84	339.06

Raw values of S10A

Mean	100	378.8	98.54	131.9
SD	24.86	19.52	13.31	37.61

123.68	400.43	113.81	157.89
74.122	362.5	89.47	88.81
102.41	373.46	92.32	149.12

Raw values of S10B

Mean	100	340.3	100.9	147.8
SD	46.33	81.64	47.12	54.61

73.29	351.49	67.30	141.68
73.56	253.67	80.65	96.45
153.67	415.80	154.76	205.17

Raw values of S10C

Mean	100	354.9	98.56	103.7
SD	29.75	42.34	23.65	40.5

126	333.33	110.33	132.33
67.66	327.66	71.33	57.33
107	403.66	114	121.33

Raw values of S10D

Mean	100	319.2	109.1	117.9
SD	7.1	87.38	7.37	16.9

108.19	238.68	100.98	136.06
96.06	306.88	115.40	102.62
95.73	412.13	110.81	115.08

Raw values of S10E

Mean	100	270.7	88.55	89.72
SD	22.64	57.98	5.78	2033

82.18	257.18	78.98	67.81
92.53	220.74	87.76	107.97
112.53	334.30	89.89	93.35
